# Rural-to-urban migrants are at high risk of sexually transmitted and viral hepatitis infections in China: a systematic review and meta-analysis

**DOI:** 10.1186/1471-2334-14-490

**Published:** 2014-09-08

**Authors:** Xia Zou, Eric PF Chow, Peizhen Zhao, Yong Xu, Li Ling, Lei Zhang

**Affiliations:** Faculty of Medical Statistics and Epidemiology, School of Public Health, Sun Yat-sen University, #74, Zhongshan Road II, Guangzhou, 510080 P.R. China; Sun Yat-sen Centre for Migrant Health Policy, Sun Yat-sen University, #74, Zhongshan Road II, Guangzhou, 510080 P.R. China; The Kirby Institute, University of New South Wales, Sydney, NSW Australia; Central Clinical School, Faculty of Medicine, Nursing and Health Sciences, Monash University, Melbourne, VIC Australia; Melbourne Sexual Health Centre, Alfred Health, Melbourne, VIC Australia

## Abstract

**Background:**

Rapid economic development in urban China has led to a mass migration of surplus rural residents into urban areas for better employment opportunities. This study aims to identify prevalence levels and risks of sexually transmitted infections (STIs) and hepatitis among the rural-to-urban migrant population in China.

**Methods:**

Chinese and English literature databases were searched for studies reporting prevalence of HIV, STIs and viral hepatitis among rural-to-urban migrants in China between 1990 and 2013. The estimates were summarised through a systematic review and meta-analysis. The risks of infection were compared between migrants and the general Chinese population.

**Results:**

We identified a total of 411 eligible studies. The prevalence of HIV, syphilis, gonorrhea, genital warts and HCV among migrants were 0.23% (0.20-0.27%), 0.69% (0.57-0.84%), 2.18% (1.30-3.64%), 1.54% (0.70-3.36%) and 0.45% (0.31-3.65%), representing 4.0 (3.1-5.2), 1.9 (1.1-3.0), 13.6 (5.8-32.1), 38.5 (15.7-94.5) and 3.8 (1.9-7.3) higher odds of infection than among the general population. Construction workers, long-distance truck drivers and migrant women through marriage were migrant subgroups that were highly susceptible to STIs and hepatitis. HIV prevalence among migrant pregnant women (0.10%, 0.02-0.49%) was significantly higher than that of pregnant women in the general Chinese population (OR = 7.7, 3.4-17.4). However, no significant differences were observed in STIs and hepatitis between overall female sex workers (FSWs), men who have sex with men (MSM) and drug users (DUs), and the corresponding subgroups with a migratory background.

**Conclusions:**

Rural-to-urban migrants have a higher risk of STIs and hepatitis than the general Chinese population, but a migratory background does not increase the infection risks of STIs and hepatitis in FSWs, MSM and DUs.

**Electronic supplementary material:**

The online version of this article (doi:10.1186/1471-2334-14-490) contains supplementary material, which is available to authorized users.

## Background

Worldwide, it’s estimated that more than one million people newly acquire STIs every day [1], mostly consisting of HIV, syphilis, gonorrhea, chlamydia, herpes simplex virus (HSV) and human papillomavirus (HPV). Hepatitis B (HBV) and C (HCV) are two commonly transmitted hepatitis infections [2]. An increasing body of evidence indicates that these hepatitis infections can also be transmitted sexually [3–6]. These STIs and hepatitis cause millions of instances of stillbirth, infertility, liver dysfunction and death every year [7]. As the most populous country in the world, China has witnessed rapidly emerging STIs and hepatitis epidemics in recent decades [8–10]. The total number of reported cases of notifiable STIs, HBV and HCV infections substantially increased from 1.39 million to 1.78 million from 2004 to 2011. The resulting number of deaths increased from 1,647 to 10,062 during the same period [11].

Due to rapid economic development in urban China, many surplus rural residents migrate to urban areas for better employment opportunities [12]. By 2013, the size of the rural-to-urban migrant population had reached 245 million [13], accounting for 20% of the total Chinese population. The large numbers of migrants surging into urban areas have substantially increased urban population density, aggravated the burden of infectious diseases and facilitated their transmission [14]. The majority of the migrant population is male, less educated and works in low-skilled and low-paid jobs [15, 16]. Being away from their spouses, migrants, especially male migrants, are more likely to participate in extramarital sex [17]. Unemployed migrants may also enter the commercial sex industry [18–22]. Approximately 10% of female migrants have participated in sex work during their stay in urban areas and consistent condom use with clients is low (15%) [23]. In addition, more than two-thirds of urban male sex workers have a migratory background [24]. An estimated 2-12% of migrants use illicit drugs [25–27].

The high mobility of migrants has facilitated the transmission of STIs and hepatitis across China [28, 29]. International studies have indicated that migrants are more susceptible to STIs and hepatitis than local residents [29–32]. However, some have disputed this view. Ojeda et al. reported migrant status was not associated with an elevated risk of STI acquisition among female sex workers in Tijuana, Mexico [33]. Others have suggested that socio-economic status and disease burden at place of origin may be stronger determinants of STI status [34–38]. This highlights the importance of the local contexts in mediating infection risks among migrants [39, 40]. Little is known about the prevalence and risk of STIs and hepatitis among Chinese migrants. Although HIV, syphilis, gonorrhea and hepatitis are notifiable under the current infectious disease case reporting system, only an estimated 10% of cases were reported [41]. Despite the establishment of the parallel STI sentinel surveillance system with 1,318 sites by 2009, very few reports on nationwide STI prevalence among migrants have been published [42]. Based on a well-constructed systematic review and meta-analysis, this study aims to: (1) assess the prevalence and risk of common STIs and hepatitis among rural-to-urban migrants in China; (2) identify migrant subgroups with the highest risk of STIs and hepatitis according to their occupations; (3) determine whether migrant status contributes to a higher risk of infection among pregnant women, female sex workers (FSWs), drug users (DUs) and men who have sex with men (MSM).

## Methods

### Definition of rural-to-urban migrants

Rural-to-urban migrants are individuals with rural residence status but who have lived in an urban area for at least six months. We employed a credible classification [43] to characterize the various subgroups of rural-to-urban migrants (Additional file 1: Table S1). These subgroups include: (1) migrants in various occupations including long-distance truck drivers, construction workers, miners, factory workers, and restaurant attendants [44] as well as migrants with unspecified job descriptions. For example, migrant women through marriage are a special subgroup of migrants who move to urban cities by marrying men with local residence. These women are predominately housewives and are classified as an occupational group [45, 46]; (2) migrant pregnant women; (3) most at-risk populations (MARPs, consisting of FSWs, DUs and MSM) with a migratory background.

### Search strategy and selection criteria

**Figure 1:**
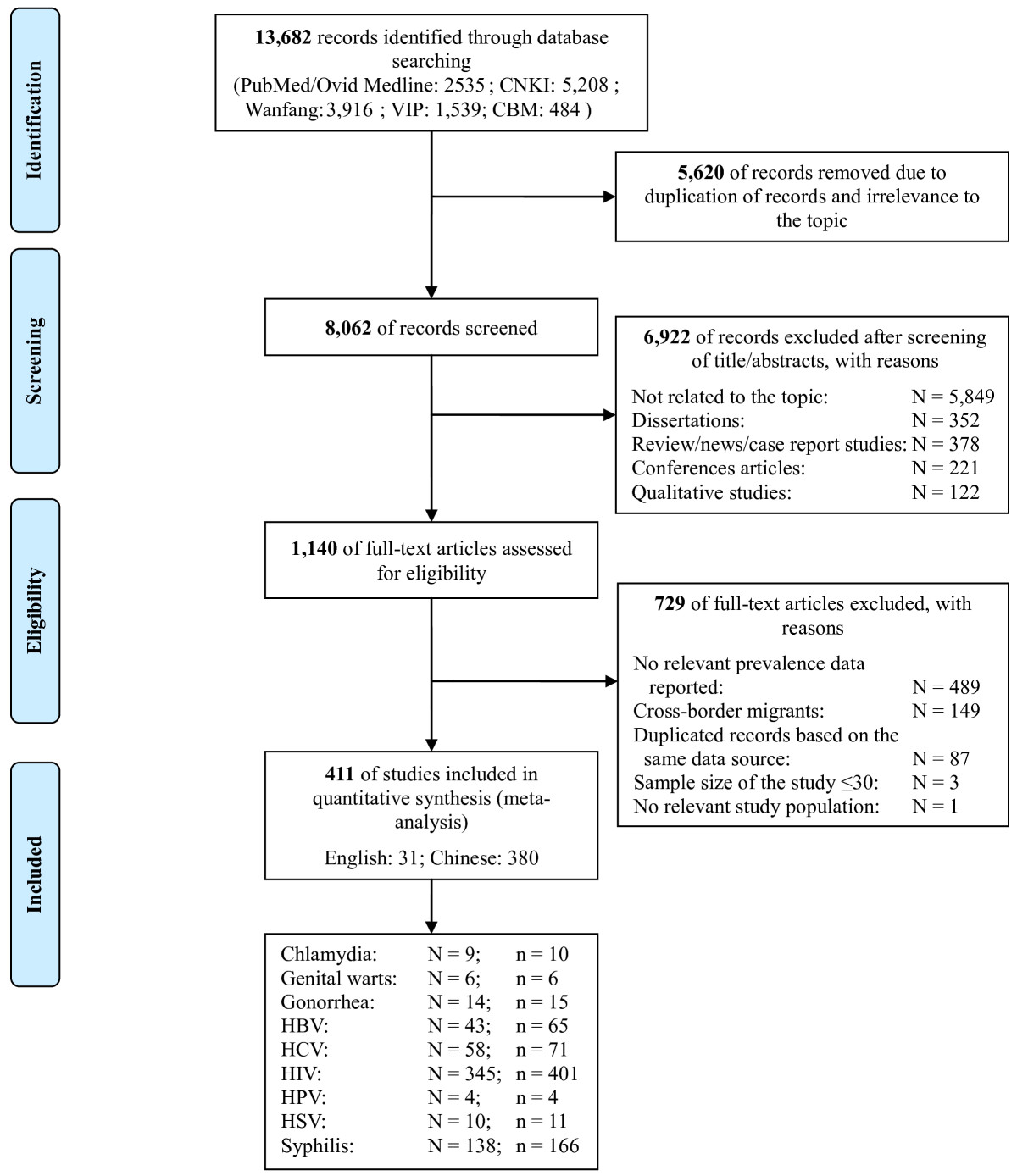
**PRISMA flow chart showing study selection processes for meta-analysis.**

We searched peer-reviewed articles that reported the prevalence of STIs, HBV and HCV among rural-to-urban migrants in six electronic databases: Chinese National Knowledge Infrastructure (CNKI), CQVIP, Wanfang data, Chinese Biomedical Literature Database, PubMed/Medline up to 30th March, 2013 (Additional file 1: Table S2). Five separate searching strategies targeting rural-to-urban migrants, migrant pregnant women, FSWs, MSM and DUs with a migratory background were used (details provided in Additional file 1: Figure S1 and Figure 1). A study was included if it: (1) reported the residence status of the targeted populations; (2) included explicit description of study methods; (3) reported the numbers of infected cases (or prevalence) and the total numbers of individuals tested for one of the following diseases: HIV, syphilis, gonorrhea, chlamydia, genital warts, HPV, HSV, HBV and HCV. Studies were excluded if the sample size was less than 30, infection status was self-reported or the same data were published in multiple publications. Two independent investigators (PZ and YX) reviewed all records to determine eligibility. Disagreements were resolved by further discussion with an additional two authors (XZ and LZ). We also identified the prevalence of all STIs and hepatitis infections among background populations based on a previously published national report and systematic review and meta-analysis studies.

### Data abstraction and quality assessment

Three authors (XZ, PZ and YX) extracted the key information independently. We recorded number of cases and total number of individuals tested for each infection. We also extracted authors, publication years, study periods, study locations, study designs, target populations, names of infections, sample sizes, and participants’ demographics to identify the characteristics of the studies and populations. We used a validated 8-item scale to assess the quality of studies [47] (Additional file 1: Table S3, Additional file 1: Figure S2).

### Statistical analysis

For each infection, studies reporting disease prevalence were pooled using a random-effects meta-analysis model [48]. Odds ratios (OR) with 95% confidence interval (CI) were used to compare risk of infection in migrants (or migrant subgroups) and the background populations. Studies were weighted based on study sample sizes. Heterogeneity was calculated by *I*^2^ statistics and a *p*-value below 0.05 was considered significant. We also conducted a temporal trend test on the prevalence level of each infection using simple linear regression. A nonparametric rank test was conducted to examine the difference between low- and high-quality publications (assessment scores ≤ 4 versus > 4). Both nonparametric rank test and ANOVA were used to identify the potential differences between diseases diagnosed methods. However, no statistical differences were found across different types of the diagnosis tests (Additional file 1: Table S4a-h). A Begg and Mazumdar rank correlation test was used to assess publication bias [49]. All analyses were conducted using the Comprehensive Meta-Analysis (version 2.2, Biostat, Englewood, New Jersey) [48]. This study was reported according to the 2009 PRISMA guideline (Additional file 1: Checklist S1) [50].

## Results

### Literature and population characteristics

A total of 411 studies published between 1997 and 2013 were included in this meta-analysis (31 English and 380 Chinese, Figure 1). These included 2,850,699 rural-to-urban migrants, 1,772,399 migrant pregnant women, 54,406 DUs (migrants: 39.03%), 65,488 FSWs (migrants: 67.38%) and 22,295 MSM (migrants: 40.44%). Migrant pregnant women (mean age 25.5 yrs) and high-risk populations with a migratory background (DUs: 28.8 yrs, FSWs: 25.5 yrs, MSM: 27.1 yrs) were younger than the overall migrant population (30.3 yrs) (Additional file 1: Table S5a-i).

### Prevalence and risk of infections

**Figure 2:**
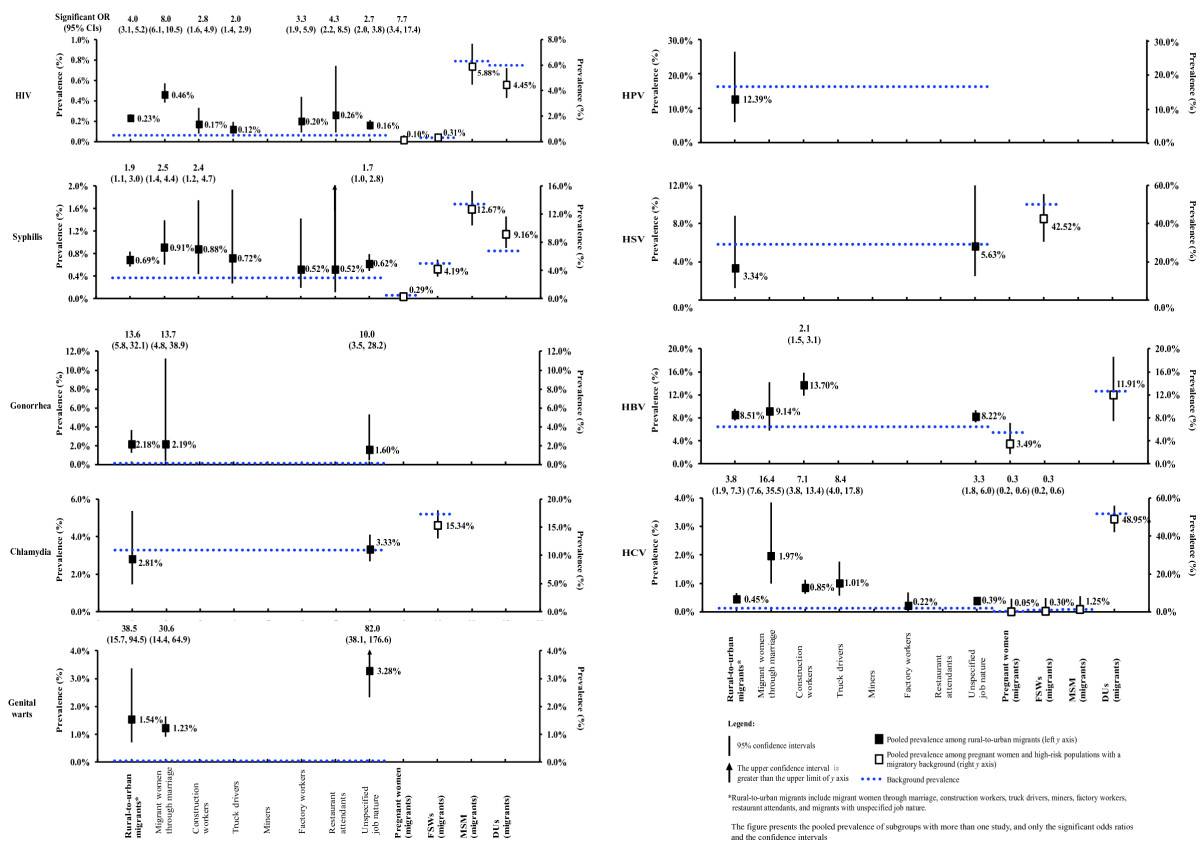
**Prevalence of STIs and viral hepatitis infections among rural-to-urban migrants and significant odds ratios compared to background populations.**

No temporal trends were found in the prevalence of all STIs and hepatitis infections (p > 0.05) (Additional file 1: Table S6). We therefore pooled all the data. The prevalence of STIs and hepatitis among rural-to-urban migrants was significantly higher than among the general adult population. In particular, prevalence of HIV, syphilis, gonorrhea and genital warts among migrants were 0.23% (0.20-0.27%), 0.69% (0.57-0.84%), 2.18% (1.30-3.64%) and 1.54% (0.70-3.36%), representing 4.0 (3.1-5.2), 1.9 (1.1-3.0), 13.6 (5.8-32.1) and 38.5 (15.7-94.5) higher odds of infection than in the general population, respectively. By contrast, 0.45% (0.31-0.65%) migrants were estimated to be infected with HCV, indicating 3.8 (1.9-7.3) higher odds of infection than the general population. The risks of HPV, HSV and HBV infections did not differ between migrants and the general population. (Additional file 1: Table S7, Figure 2).

Among various migrant occupation subgroups, migrant women through marriage consistently bear high risks of STIs and HCV infection. Prevalence of HIV (0.46%, 0.38-0.57%), syphilis (0.91%, 0.60-1.39%), gonorrhea (2.19%, 0.40-11.20%), genital warts (1.23%, 0.91-1.64%) and HCV (1.97%, 1.00-3.85%) among migrant women were significantly higher than those of the general population (HIV: OR = 8.0, 6.1-10.5; syphilis: OR = 2.5, 1.4-4.4, gonorrhea: OR = 13.7, 4.8-38.9, genital warts: OR = 30.6, 14.4-64.9 and HCV: OR = 16.4, 7.6-35.5). Construction workers also demonstrated significantly higher prevalence levels in HIV (0.17%, 0.08-0.33%; OR = 2.8, 1.6-4.9), syphilis (0.88%, 0.44-1.74%; OR = 2.4, 1.2-4.7), HBV (13.70%, 11.84-15.80%, OR = 2.1, 1.5-3.1) and HCV (0.85%, 0.64-1.13%; OR = 7.1, 3.8-13.4) than the general population. Further, long-distance truck drivers showed significantly higher prevalence of HIV (0.12%, 0.08-0.19%; OR = 2.0, 1.4-2.9) and HCV (1.01%, 0.57-1.76%; OR = 8.4, 4.0-17.8) only. Restaurant attendants, factory workers and those with unspecified job descriptions also showed higher prevalence of HIV, but not in other STIs or hepatitis.

HIV prevalence among migrant pregnant women (0.10%, 0.02-0.49%) was significantly higher than pregnant women in the general Chinese population (OR = 7.7, 3.4-17.4). However, no significant differences were observed in STIs and hepatitis between background population FSWs, MSM and DUs and the corresponding subgroups with a migratory background.

### Heterogeneities and publication bias

High heterogeneities were observed in most of the meta-analysis of STIs, HBV and HCV (Additional file 1: Figure S3). Begg’s test revealed potential publication biases in studies on HIV, syphilis and HSV prevalence among migrants (HIV: p < 0.01, syphilis: p = 0.02, HSV: p < 0.05) and HIV among migrant FSWs (p < 0.01), studies on HCV among migrants with unspecified job descriptions (p = 0.01) and MSM with a migratory background (p < 0.01). The averaged study quality score was five (Additional file 1: Figure S2). The prevalence levels between low- and high-quality score groups (≤4 vs. >4) showed no statistical differences (p > 0.05) (Additional file 1: Table S8).

## Discussion

Our findings provide evidence that rural-to-urban migrants are subjected to a higher risk of STIs and hepatitis than the Chinese population as a whole. Of these, migrant women, construction workers and long-distance truck drivers are migrant subgroups that are the most susceptible to STIs, HBV and HCV infections. Migrant pregnant women demonstrate significantly higher HIV prevalence than pregnant women in the general Chinese population, but the risks of STIs and hepatitis do not differ between MARPs and their corresponding migrant subpopulations.

The risks of STIs and hepatitis are probably related to demographic characteristics and sexual behavior. Migrants who are construction workers and long-distance truck drivers are predominantly male and more likely to solicit commercial sex workers than migrants of other occupations [51]. Frequent sex encounters as well as low perception of risk, low STI knowledge and low rates of condom use were reported among these occupational groups. In particular, consistent with previous findings [29, 30], a high level of STIs and HCV infection has been reported in this analysis, The migratory status and risky sexual behaviors among long-distance truck drivers may both contribute to the greater risk of STIs [52, 53] In addition, long-distance truck drivers are found to be more likely to engage in illicit drug use than the general population which may lead to higher risks of HCV infection [54, 55]. By contrast, women who migrated due to marriage are a specific migrant subgroup. Demographic studies have suggested that most of these women come from a less-developed Southwest and Northwest China which geographically overlap with areas of high STIs and HCV transmission [56–59]. These women may have been already infected prior to their migration [57]. Prevention of the further spread of these infections to their serodiscordant husbands should be a priority for this migrant subgroup.

The high levels of HIV infection among migrant pregnant women are alarming. Prevention of mother-to-child transmission (PMTCT) of HIV has been initiated since 2003 under the China’s ‘Four Free One Care’ policy and the program was further expanded to cover syphilis and HBV infection in 2009 [60]. The PMTCT program has been shown to be highly effective in reducing vertical transmission of HIV by 60-80% among participants in care [61–63]. However, high mobility, low socio-economic status and poor perception about the transmission of STIs and hepatitis [64] among migrant pregnant women remain as major barriers to timely PMTCT services [65].

Migratory background plays only a small role in the most at-risk populations. The proportion of DUs (39.03%) and MSM (40.44%) who are migrants is comparable to the actual proportion of rural-to-urban migrants in urban China (20.00-37.42%) [66, 67]. By contrast, as the majority of FSWs (67.38%) already have a migratory background, the comparison of infection risks between FSWs and its migrant subgroup may not be meaningful, as the expected differences are insignificant. Among DUs and MSM, having multiple sexual partners and unprotected sex, and using unsafe intravenous needle are the major risk factors for STIs and hepatitis [68–71]. These risks likely dominate the risks from occasional casual and commercial activities associated with migrants. Nevertheless, the high proportion of FSWs with a migratory background indicates that intervention programs targeting migrant FSWs could have extended their impact over the overall FSWs population in China.

A number of limitations to this study should be noted. First, despite a thorough search and literature review, very few studies provide matching disease status among non-migrants as a reference for direct prevalence comparison with migrants. We used the overall general Chinese population for comparison. Second, heterogeneities across studies remain significant despite of detailed subgroup categorization and analyses. We did not find strong evidence for significant temporal variation of disease prevalence (consistent with a previous study [72]), but we acknowledge that the differences in specificity and sensitivity of diagnosis tests may have contributed to the heterogeneities in prevalence levels. Other factors, such as study location and sampling method may also have contributed to the heterogeneities. Third, publication bias exists in some sub-group analyses. Regions that are more developed or have higher disease burdens may have been more likely to contribute to our data.

This study indicates disproportionately high disease burdens of STIs and hepatitis among rural-to-urban migrants in China. With its growing population size, timely prevention and treatment of these infections for migrants remains a high priority. Surveillance and prevention efforts need to be sensitive to migrants’ demographic backgrounds and occupations. Further expansion of the PMTCT program is necessary to provide sufficient access to migrant pregnant women regardless of their residence status.

## Conclusions

Overall, rural-to-urban migrants have higher risks of STIs and hepatitis than the Chinese general population, but migratory background does not increase the risks of STIs and hepatitis infections among FSWs, MSM and DUs.

## Electronic supplementary material

Additional file 1: Figure S1: Search strategies for literature reporting prevalence of STIs and viral hepatitis among rural-to-urban migrants, migrant pregnant women, and the most at-risk populations with a migratory background. **Figure S2.** Total score of quality assessment for meta-analysis. **Figure S3.** Forest plots of meta-analysis of STI and hepatitis prevalence among rural-to-urban migrants in China. **Table S1.** Classification and definitions of subgroup migrants included in meta-analysis. **Table S2.** Introduction and descriptions of four Chinese databases used in meta-analysis. **Table S3.** Quality assessment checklist for meta-analysis. **Table S4.** nonparametric test and ANOVA test for combinations of HIV tests. **Table S5.** Characteristics description of studies included in meta-analysis of HIV prevalence. **Table S6.** Time trend test for studies included in meta-analysis. **Table S7.** Prevalence and odds ratios of sexually transmitted viral infections among rural-to-urban migrants, migrant pregnant women and high-risk groups with a migratory background compared with national prevalence or systematic review studies among background population. **Table S8.** Comparison of prevalence levels of infections of low-risk subgroup population between low- and high-quality groups. **Checklist S1.** PRISMA Checklist for meta-analysis. (DOC 17 MB)

Below are the links to the authors’ original submitted files for images.

Authors’ original file for figure 1

Authors’ original file for figure 2

## References

[1] Sexually transmitted infections (STIs): the importance of a renewed commitment to STI prevention and control in achieving global sexual and productive health. http://www.who.int/reproductivehealth/publications/rtis/rhr13_02/en/index.html,

[2] Viral hepatitis. http://www.who.int/hiv/topics/hepatitis/hepatitisinfo/en/,

[3] Barua P, Laskar N, Medhi GK, Apum B, Mahanta J (2008). Sexual Transmission of Hepatitis C Virus Among Female Sex Workers in India. Int J Infect Dis.

[4] Kim AY, Chung RT (2007). Sex, drugs, and hepatitis c virus in men who have sex with men: evidence for permucosal transmission. Gastroenterology.

[5] Prevention CfDCa: 2011, 945-950. Sexual transmission of hepatitis C virus among HIV-infected men who have sex with men--New York City, 2005–2010, Morbidity and Mortality Weekly Report (MMWR), Volume 60 21775948

[6] de Paula CN, de la Rosa A, Elagin S, Tengan FM, Barone AA (2010). Hepatitis C virus: molecular and epidemiological evidence of male-to-female transmission. Braz J Infect Dis.

[7] Sexually transmitted infections. http://www.who.int/mediacentre/factsheets/fs110/en/,

[8] Zhang L, Wilson DP (2012). Trends in notifiable infectious diseases in China: implications for surveillance and population health policy. PLoS One.

[9] Chow EP, Tucker JD, Wong FY, Nehl EJ, Wang Y, Zhuang X, Zhang L (2014). Disparities and risks of sexually transmissible infections among men who have sex with men in China: a meta-analysis and data synthesis. Plos ONE.

[10] Hajarizadeh B, Grebely J, Dore GJ (2013). Epidemiology and natural history of HCV infection. Nat Rev Gastroenterol Hepatol.

[11] Prevention CCfDCa (2013). National report of notifiable diseases in China (2007–2013). National Report of Notifiable Diseases in China.

[12] Cui X, Rockett IR, Yang T, Cao R (2012). Work stress, life stress, and smoking among rural–urban migrant workers in China. BMC Public Health.

[13] The Department Services and Management of Migrant Population of National Population and Family Planning Commission of China (2012). Report on China's migrant population development.

[14] Hui BB, Gray RT, Wilson DP, Ward JS, Smith AM, Philip DJ, Law MG, Hocking JS, Regan DG (2013). Population movement can sustain STI prevalence in remote Australian indigenous communities. BMC Infect Dis.

[15] Hesketh T, Jun YX, Lu L, Mei WH (2008). Health status and access to health care of migrant workers in China. Public Health Rep.

[16] Han K, Huang C-C, Han W (2011). Social mobility of migrant peasant workers in China. Sociol Mind.

[17] Wang W, Wei C, Buchholz ME, Martin MC, Smith BD, Huang ZJ, Wong FY (2010). Prevalence and risks for sexually transmitted infections among a national sample of migrants versus non-migrants in China. Int J STD AIDS.

[18] Rogers SJ, Ying L, Xin YT, Fung K, Kaufman J (2002). Reaching and identifying the STD/HIV risk of sex workers in Beijing. AIDS Educ Prev.

[19] van den Hoek A, Yuliang F, Dukers NH, Zhiheng C, Jiangting F, Lina Z, Xiuxing Z (2001). High prevalence of syphilis and other sexually transmitted diseases among sex workers in China: potential for fast spread of HIV. AIDS.

[20] Qian HZ, Vermund SH, Wang N (2005). Risk of HIV/AIDS in China: subpopulations of special importance. Sex Transm Infect.

[21] Zhu TF, Wang CH, Lin P, He N (2005). High risk populations and HIV-1 infection in China. Cell Res.

[22] Zheng L, Zhu J, Tian P, Chen Y, Song Y, Chen Y (2000). Sexual behaviors and associated factors among unmarried female migratory workers in Guangzhou. Chin J Fam Plann.

[23] Yang H, Li X, Stanton B, Fang X, Lin D, Mao R, Liu H, Chen X, Severson R (2005). Workplace and HIV-related sexual behaviours and perceptions among female migrant workers. AIDS Care.

[24] Chow EPF, Iu KI, Fu X, Wilson DP, Zhang L (2012). HIV and sexually transmissible infections among money boys in China: a data synthesis and meta-analysis. PLoS One.

[25] Pan X, Zhu Y, Wang Q, Zheng H, Chen X, Su J, Peng Z, Yu R, Wang N (2013). Prevalence of HIV, syphilis, HCV and their high risk behaviors among migrant workers in eastern China. PLoS One.

[26] He N, Wong FY, Huang ZJ, Thompson EE, Fu C (2007). Substance use and HIV risks among male heterosexual and 'money boy' migrants in Shanghai, China. AIDS Care.

[27] Wong FY, He N, Huang ZJ, Young D, O'Conor C, Ding YY, Fu C, Arayasirikul S (2010). Migration and illicit drug use among two types of male migrants in Shanghai, China. J Psychoactive Drugs.

[28] Liu Y, Li X, Zhang L, Li S, Jiang S, Stanton B (2012). Correlates of consistent condom use among young migrant men who have sex with men (MSM) in Beijing, China. Eur J Contracept Reprod Health Care.

[29] Zhang L, Chow EP, Jahn HJ, Kraemer A, Wilson DP (2013). High HIV prevalence and risk of infection among rural-to-urban migrants in various migration stages in China: a systematic review and meta-analysis. Sex Transm Dis.

[30] Zhang X, Chow EP, Wilson DP, Sun X, Zhao R, Zhang J, Jing J, Zhang L (2013). Prevalence of HIV and syphilis infections among long-distance truck drivers in China: a data synthesis and meta-analysis. Int J Infect Dis.

[31] Wong WC, Yim YL, Lynn H (2011). Sexually transmitted infections among female sex workers in Hong Kong: the role of migration status. J Travel Med.

[32] Mmbaga EJ, Leyna GH, Hussain A, Mnyika KS, Sam NE, Klepp K-I (2008). The role of in-migrants in the increasing rural HIV-1 epidemic: results from a village population survey in the Kilimanjaro region of Tanzania. Int J Infect Dis.

[33] Ojeda VD, Strathdee SA, Lozada R, Rusch ML, Fraga M, Orozovich P, Magis-Rodriguez C, De La Torre A, Amaro H, Cornelius W, Patterson TL (2009). Associations between migrant status and sexually transmitted infections among female sex workers in Tijuana, Mexico. Sex Transm Infect.

[34] Organista KC, Carrillo H, Ayala G (2004). HIV prevention with Mexican migrants: review, critique, and recommendations. J Acquir Immune Defic Syndr.

[35] Barnett ED, Walker PF (2008). Role of immigrants and migrants in emerging infectious diseases. Med Clin N Am.

[36] Folch C, Esteve A, Sanclemente C, Martro E, Lugo R, Molinos S, Gonzalez V, Ausina V, Casabona J (2008). Prevalence of human immunodeficiency virus, Chlamydia trachomatis, and Neisseria gonorrhoeae and risk factors for sexually transmitted infections among immigrant female sex workers in Catalonia. Spain Sex Transm Dis.

[37] Xiridou M, van Veen M, Coutinho R, Prins M (2010). Can migrants from high-endemic countries cause new HIV outbreaks among heterosexuals in low-endemic countries?. AIDS.

[38] Xiridou M, van Veen M, Prins M, Coutinho R (2011). How patterns of migration can influence the heterosexual transmission of HIV in The Netherlands. Sex Transm Infect.

[39] Dave SS, Copas A, Richens J, White RG, Kosambiya JK, Desai VK, Stephenson JM (2012). HIV and STI prevalence and determinants among male migrant workers in India. PLoS One.

[40] Platt L, Grenfell P, Fletcher A, Sorhaindo A, Jolley E, Rhodes T, Bonell C (2013). Systematic review examining differences in HIV, sexually transmitted infections and health-related harms between migrant and non-migrant female sex workers. Sex Transm Infect.

[41] Lin CC, Gao X, Chen XS, Chen Q, Cohen MS (2006). China's syphilis epidemic: a systematic review of seroprevalence studies. Sex Transm Dis.

[42] Meng X, Wang L, Chan S, Reilly KH, Peng Z, Guo W, Ding G, Ding Z, Qin Q (2011). Estimation and projection of the HIV epidemic trend among the migrant population in China. Biomed Environ Sci.

[43] Chan KW (2012). “Internal Labor Migration in China: Trends, Geography and Policies”. United Nations Population Division, Population Distribution, Urbanization, Internal Migration and Development: An International Perspective.

[44] He N (2007). Sociodemographic characteristics, sexual behavior, and HIV risks of rural-to-urban migrants in China. Biosci Trends.

[45] Pan XH, Yang JZ, Chen L, Xu Y (2010). [Analysis of epidemiological characteristics of HIV infections among immigrant marriage women in rural areas in Zhejiang province]. Zhonghua Yu Fang Yi Xue Za Zhi.

[46] Fu J, Zhang Q, Lv F, Liu X, Zhang X (2006). [Demographic characteristics of immigrant women in a rural district of Shandong province]. Chin J Aids STD.

[47] Szatmari P (1998). Guidelines for evaluating prevalence studies. Evid Based Ment Health.

[48] Borenstein M, Hedges LV, Higgins JPT, Rothstein HR (2009). Introduction to Meta-Analysis.

[49] Begg CB, Mazumdar M (1994). Operating characteristics of a rank correlation test for publication bias. Biometrics.

[50] Moher D, Liberati A, Tetzlaff J, Altman DG (2010). Preferred reporting items for systematic reviews and meta-analyses: the PRISMA statement. Int J Surg.

[51] Jiang X (2008). Exploratory study influencing condom on HIV related sexual risk behaviors and factors influencing condom use behaviour change among floating population.

[52] Wong WC, Tam SM, Leung PW (2007). Cross-border truck drivers in Hong Kong: their psychological health, sexual dysfunctions and sexual risk behaviors. J Travel Med.

[53] Zhuang X, Wu Z, Poundstone K, Yang C, Zhong Y, Jiang S (2012). HIV-related high-risk behaviors among Chinese migrant construction laborers in Nantong. Jiangsu PLoS One.

[54] Freitas N, Teles S, Matos M, Lopes C, Reis N, Espírito-Santo M, Lampe E, Martins R (2010). Hepatitis C virus infection in Brazilian long-distance truck drivers. Virol J.

[55] Changhe F, Wei H, Desen Y (2004). The third national epidemiological survey on illicit drug use at six high prevalence areas in China (part III: social demographics of illicit drug use). Chin J Drug Depend.

[56] Fan X (2009). Evaluation on effectiveness of HIV/AIDS health education among the immigrant women in the rual areas.

[57] Xie Z (2007). The current status of HIV/AIDS epidemic in Jinan city and the effect of immigrant women on its epidemic.

[58] Xia X, Luo J, Bai J, Yu R (2008). Epidemiology of hepatitis C virus infection among injection drug users in China: systematic review and meta-analysis. Publ Health.

[59] Bao YP, Liu ZM (2009). Systematic review of HIV and HCV infection among drug users in China. Int J STD AIDS.

[60] Health M (2011). AIDS Prevention Mother to Child Transmission of Syphilis and Hepatitis B Work Plan.

[61] Peng Y, Zhu Q, Hu J, Sun D, Wang Q, Wang C, Wang W, Jiang Y (2008). Evaluation on a model of HIV/AIDS prevention of mother-to-child transmission in Henan Province. Matern Child Health Care Chin.

[62] Li B, Zhao Q, Zhang X, Wu L, Chen T, Liang Z, Xu L, Yu S (2013). Effectiveness of a prevention of mother-to-child HIV transmission program in Guangdong province from 2007 to 2010. BMC Public Health.

[63] Cheng W, Zi X, Zhang L (2009). Evaluation of effectiveness on prevention of HIV mother-to-child transmission. Modern Prev Med.

[64] Qian HZ, Schumacher JE, Chen HT, Ruan YH (2006). Injection drug use and HIV/AIDS in China: review of current situation, prevention and policy implications. Harm Reduct J.

[65] Qin M, Qian L, Cheng L, Jia Y, Cai Q (2012). HIV management among migrant pregnant women in Kunming: policy implications. Chin J Publ Health Manag.

[66] China Statistical Yearbook. 2011, http://www.stats.gov.cn/tjsj/ndsj/2012/indexch.htm,

[67] Bao C (2007). Analysis on the characteristic of migrant population in 286 cities in China. Popul Res.

[68] Chow EP, Wilson DP, Zhang L (2011). What is the potential for bisexual men in China to act as a bridge of HIV transmission to the female population? Behavioural evidence from a systematic review and meta-analysis. BMC Infect Dis.

[69] Chow EP, Wilson DP, Zhang L (2012). Patterns of condom use among men who have sex with men in China: a systematic review and meta-analysis. AIDS Behav.

[70] Zhuang X, Wang Y, Chow EP, Liang Y, Wilson DP, Zhang L (2012). Risk factors associated with HIV/HCV infection among entrants in methadone maintenance treatment clinics in China: a systematic review and meta-analysis. Drug Alcohol Depend.

[71] Zhang L, Chow EP, Wilson DP (2012). Distributions and trends in sexual behaviors and HIV incidence among men who have sex with men in China. BMC Public Health.

[72] Cai R, Richardus JH, Looman CW, de Vlas SJ (2013). Trends in high-risk sexual behaviors among general population groups in China: a systematic review. PLoS One.

[CR73] The pre-publication history for this paper can be accessed here:http://www.biomedcentral.com/1471-2334/14/490/prepub

