# Supplementary material for: Rural-to-urban migrants are at high risk of sexually transmitted and viral hepatitis infections in China: a systematic review and meta-analysis
Source: BMC Infect Dis. 2014 Sep 8;14:490. doi: 10.1186/1471-2334-14-490 (PMC4169821; doi:10.1186/1471-2334-14-490)
Supplement: Supplementary file 1 — Additional file 1: Figure S1: Search strategies for literature reporting prevalence of STIs and viral hepatitis among rural-to-urban migrants, migrant pregnant women, and the most at-risk populations with a migratory background. Figure S2. Total score of quality assessment for meta-analysis. Figure S3. Forest plots of meta-analysis of STI and hepatitis prevalence among rural-to-urban migrants in China. Table S1. Classification and definitions of subgroup migrants included in meta-analysis. Table S2. Introduction and descriptions of four Chinese databases used in meta-analysis. Table S3. Quality assessment checklist for meta-analysis. Table S4. nonparametric test and ANOVA test for combinations of HIV tests. Table S5. Characteristics description of studies included in meta-analysis of HIV prevalence. Table S6. Time trend test for studies included in meta-analysis. Table S7. Prevalence and odds ratios of sexually transmitted viral infections among rural-to-urban migrants, migrant pregnant women and high-risk groups with a migratory background compared with national prevalence or systematic review studies among background population. Table S8. Comparison of prevalence levels of infections of low-risk subgroup population between low- and high-quality groups. Checklist S1. PRISMA Checklist for meta-analysis. (DOC 17 MB) [file 12879_2014_3800_MOESM1_ESM.doc]

**Additional file 1**

**Table S1 Classification and definitions of subgroup migrants included in meta-analysis**

| Risks | Subgroups | Classification* | Definitions |
| --- | --- | --- | --- |
| Low-risk | Construction workers | Series F | This series refers to what is literally known as "rural migrant labor" (*nonmingong*). This group includes only the working population with rural-*hukou* but not local residency at destination. |
|  | Miners | Series F |  |
|  | Factory workers | Series F |  |
|  | Restaurant attendants | Series F |  |
|  | Migrants with unspecified job natures | Series F |  |
|  | Migrant women through marriage | Series A | This series refers to migrants who are formally granted *hukou* status in the destination (city, town and township) each year, excluding those moves within a city. A portion of *hukou* migrations is involved with migration through marriage. |
|  | Long-distance truck drivers | Series B | Series B refers to the broadest and most widely used definition of the floating population, which includes anyone without local *hukou* staying in the destination, regardless of the length of the stay in a place. The stay can be as short as overnight or for several years.  Series C refers to anyone staying in places other than his/her household registration for three days or more and is registered with the police and have applied for a *zanzhuzheng* ("temporary resident permit")  Series D and E: These series stipulate a far longer minimum residence requirement (6 months or one year) than Series B and C and excluded the most temporary, such as tourists, and short-term migrant workers. These series refers to as the most commonly known *liudongrenkou* (“floating population”). |
|  | Migrant pregnant women | Series B/C/D/E |  |
| High-risk | FSWs | Series B |  |
|  | MSM | Series B |  |
|  | DUs | Series B |  |

*Classification reference: Chan, Kam Wing, “China, Internal Migration,” in Immanuel Ness and Peter Bellwood, eds. The Encyclopedia of Global Migration, Blackwell Publishing, 2012b

**Table S2 Introduction and descriptions of four Chinese databases used in m**eta-analysis

| **Items** | **CQVIP** | **CNKI** | **Wanfang** | **CBM** |
| --- | --- | --- | --- | --- |
| Full name | Chong Qing VIP Chinese scientific journal database | Chinese National Knowledge Infrastructure | Wanfang Data | Chinese Biomedical Literature Database |
| Scope of research | Health and medicine, social science, natural science, engineering, agricultural science, economic management, educational science, library and information science etc. | Science engineering, agriculture, health and medicine, philosophy, economic and political, law, education and social science, electronic and information science etc. | Philosophy and political science, social science, economic and financial science, education and culture, preclinical medicine, health and medicine, agriculture science, industrial technology etc. | Preclinical medicine, clinical medicine, preventive medicine, pharmacology, traditional Chinese medicine etc. |
| No. of periodicals covered | ~ 12000 | ~ 9800 | ~ 7000 | ~ 1800 |
| Year | 1989- | 1994- | 1998- | 1978- |
| No. of literatures (2012)[^1^](#_ENREF_1) | 30 millions | 37.2 millions | 20 millions | 7 millions |
| Coverage of all periodicals[^2^](#_ENREF_2) | 58.89% | 56.36% | 52.69% | —— |
| Update frequencies[^1^](#_ENREF_1) | 1.8 million times per year | everyday | twice a week | once a month |
| Coverage of all Chinese core journals[^3^](#_ENREF_3) | 91% | 100% | 98% | —— |

**Reference:**

**1.** Tian J, Yang K, Song G, Wei Z. Comparative Study of Four Medical and Health Databases in China. *Library and Information.* 2008(5):64-67.

**2.** Du Y, Lei C, Chen J, et al. A Comparative Study of the Literature Included in the Five Authoritative Domestic Databses of Chinese Biomedical Journals. *J Prev MedInf.* 2006;22(2):162-166.

**3.** Cao K. Comparative Study of the Quality of Data in Three Chinese Medical Journal Database. *Library Theory and Practice.* 2008(3):24-28.

**Figure S1: Searching strategies for meta-analysis of STDs, HBV and HCV among rural-to-urban migrants, migrant pregnant women, most at-risk populations with a migratory background**

AND

AND

AND

AND

AND

AND

AND

**Keywords:** ‘**China**’ OR ‘**Chinese**’

AND

AND

AND

**Keywords on rural-to-urban migrants:**

‘**rural-to-urban**’ OR

‘**peasant worker(s)**’ OR

‘**domestic migrant(s)**’ OR

‘**mobile population**’ OR

‘**floating population**’ OR

‘**migrant worker(s)**’ OR

‘**peasant labor(s)**’ OR

‘**migrant(s)**’ OR

‘**miner(s)**’ OR

‘**immigrant women**’ OR

‘**Non Min Gong**’ (Chinese term means migrant workers) OR

‘**Wai Lai Wu Gong Ren Yuan**’ (Chinese term means migrant workers) OR

‘**Wai Chu Wu Gong Ren Yuan**’ (Chinese term means migrant workers)

**Keywords on pregnant women:**

‘**pregnant**’ OR

‘**pregnancy**’ OR

‘**prenatal**’ OR

‘**vertical transmission**’ OR

‘**mother-to-child transmission**’ OR

‘**maternal-neonatal**’ OR ‘**mother-child**’ OR

‘**perinatal transmission**’ OR

‘**post-natal transmission**’ OR

‘**maternal-fetal**’ OR

‘**Yun Chan Fu** (Chinese Term meaning Pregnant women)

**Keywords on FSWs:**

‘**female sex worker**’ OR

‘**FSW**’ OR

‘**commercial sex worker**’ OR

‘**CSW**’ OR

‘**An Chang**’ (Chinese term means female sex workers) OR

‘**Xiao Jie**’ (Chinese term means female sex workers)

**Keywords on MSM:**

‘**homosexual**’ OR

‘**gay**’ OR

‘**bisexual**’ OR

‘**men who have sex with men**’ OR

‘**MSM**’ OR

‘**Tong Zhi**’ (Chinese term means men who have sex with men)

**Keywords on MSM:**

‘**drug users**’ OR

‘**heroin addicts**’ OR

‘**Xi Du**’ (Chinese term means drug use)

**Keywords on STDs and hepatitis:**

‘**sexually transmitted infection(s)**’ OR **‘STD(s)**’ OR ‘**sexually transmitted disease(s)**’ OR ‘**STI(s)**’ OR ‘**venereal disease(s)**’ OR ‘**syphilis**’ OR ‘**treponema**’ OR ‘**gonorrhea**’ OR ‘**chlamydia**’ OR ‘**genital warts**’ OR ‘**HPV**’ OR ‘**papilloma**’ OR ‘**HSV**’ OR ‘**herpes simplex virus**’ OR ‘**HBV**’ OR ‘**HCV**’ OR ‘**hepatitis B**’ OR ‘**hepatitis C**’

**Table S3** Quality assessment checklist for meta-analysis

| **ID** | **Questions for Evaluating Prevalence Studies** | **Decision Criteria** | **Score** |
| --- | --- | --- | --- |
| Q1 | Was the population from which the sample was drawn clearly defined? | Yes: Background information, including: (i) study duration (i.e. month and year) and (ii) recruitment locations (e.g. restaurant, factories) were identified clearly. | 1 |
|  |  | No: Background information, neither: (i) study duration or recruitment locations (e.g. restaurant, factories) were not identified. | 0 |
| Q2 | Was the sampling method representative of the population intended to the study? | Yes: Probability sampling (including: simple random, systematic, stratified, cluster, two-stage and multi-stage sampling) was adopted | 1 |
|  |  | No: Non-probability sampling (including: purpose, quota, convenience, and snowball sampling) was adopted. | 0 |
| Q3 | Did the characteristics of respondents match the target population? | Yes: Inclusion/exclusion criteria in sampling selection were reported | 1 |
|  |  | No: Inclusion/exclusion criteria in sample selection were not reported. | 0 |
| Q4 | Was the response rate adequate? | Yes: Response rate was≥80 (i.e. rejection rate<20) | 1 |
|  |  | No: Response rate was not reported or <80 | 0 |
| Q5 | Were the data collection methods standardized? | Yes: Identical methods of assessment and data collection were used to all respondents | 1 |
|  |  | No: Methods of assessment and data collection to all respondents were not identical | 0 |
| Q6 | Were measures shown to be reliable? | Yes: Study clearly reported the (i) survey instrument: survey was piloted, adopted/adapted from other study (with reference) or tested by Cronbach's alpha; (ii) biomarkers and laboratory tests used for HIV/STIs diagnosis was identified clearly. | 1 |
|  |  | No: Study did not report the survey instrument, biomarkers and laboratory tests used for HIV/STIs diagnosis. | 0 |
| Q7 | Were measures shown to be valid? | Yes: The method/procedure of screening and/or confirmation test was reported. | 1 |
|  |  | No: The method/procedure of screening and/or confirmation test was not reported. | 0 |
| Q8 | Were the statistical methods appropriate? | Yes: Correct statistical tests were used. Confident intervals or standard deviation/variance were given for prevalence rate | 1 |
|  |  | No: Incorrect statistical tests were used. Only the prevalence rate was reported (confident intervals or standard deviation was not calculated) | 0 |

**Figure S2 Total score of quality assessment for meta-analysis**


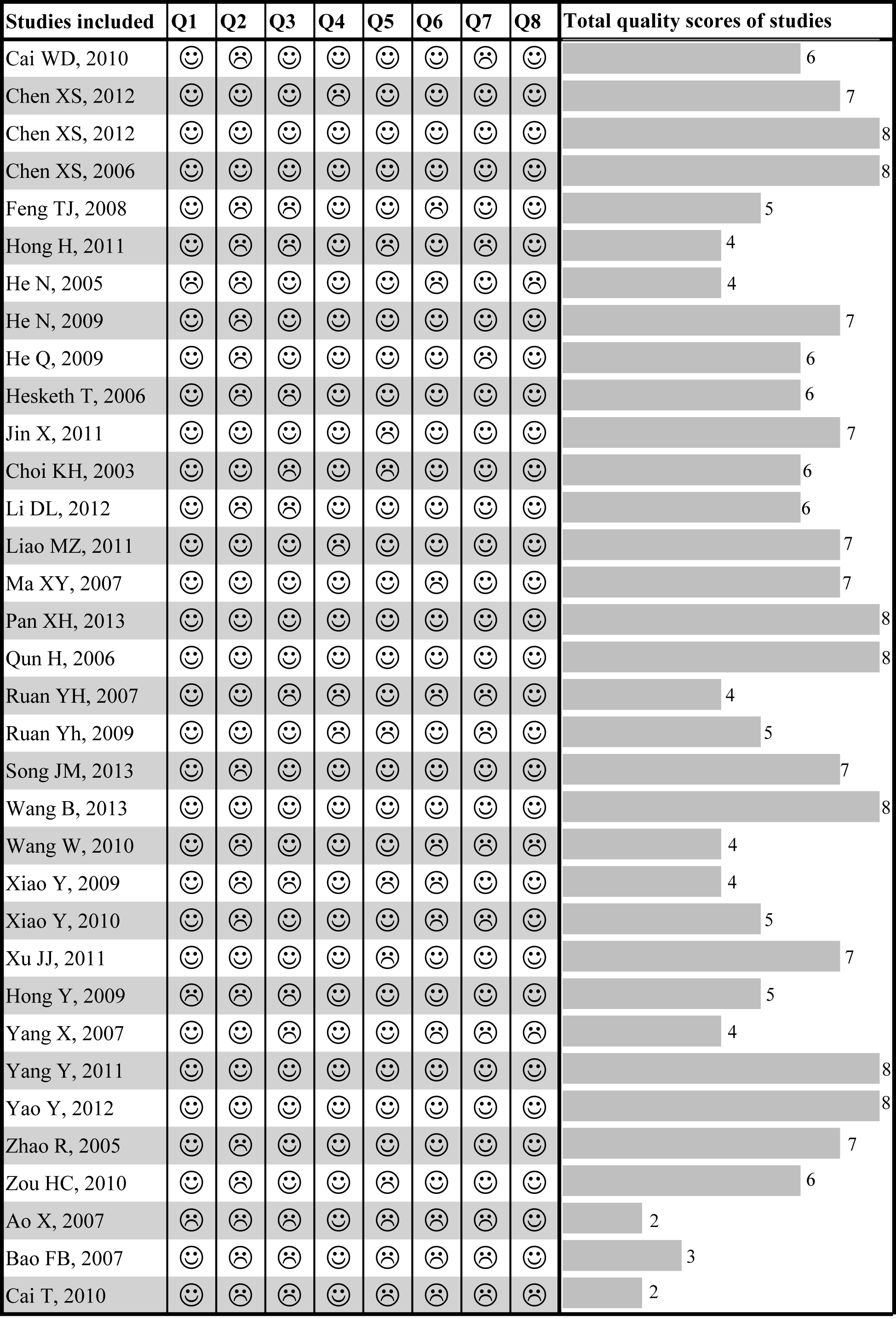
 **Figure S2 Total score of quality assessment for meta-analysis (Cont’d)**


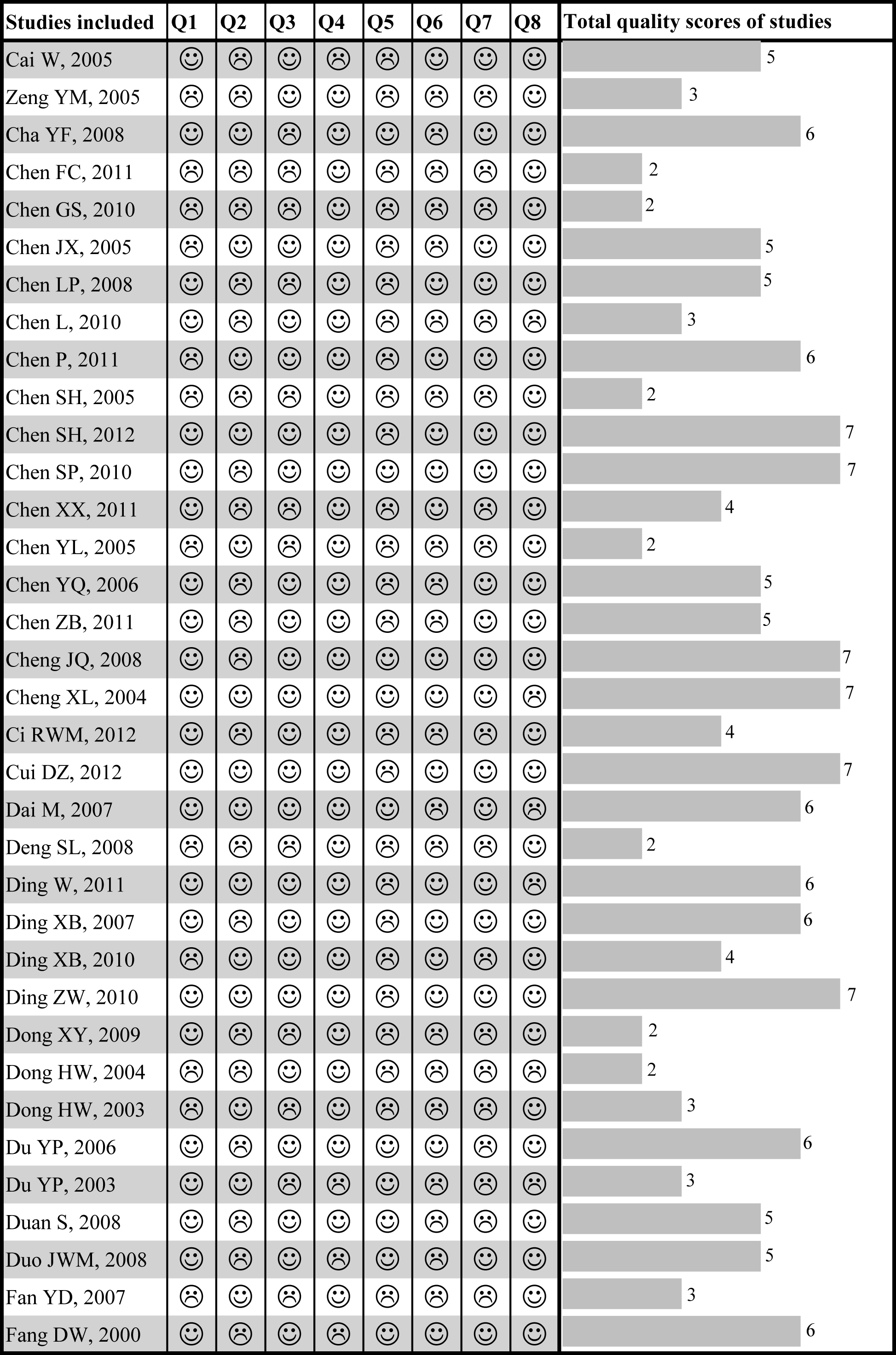


**Figure S2 Total score of quality assessment for meta-analysis (Cont’d)**


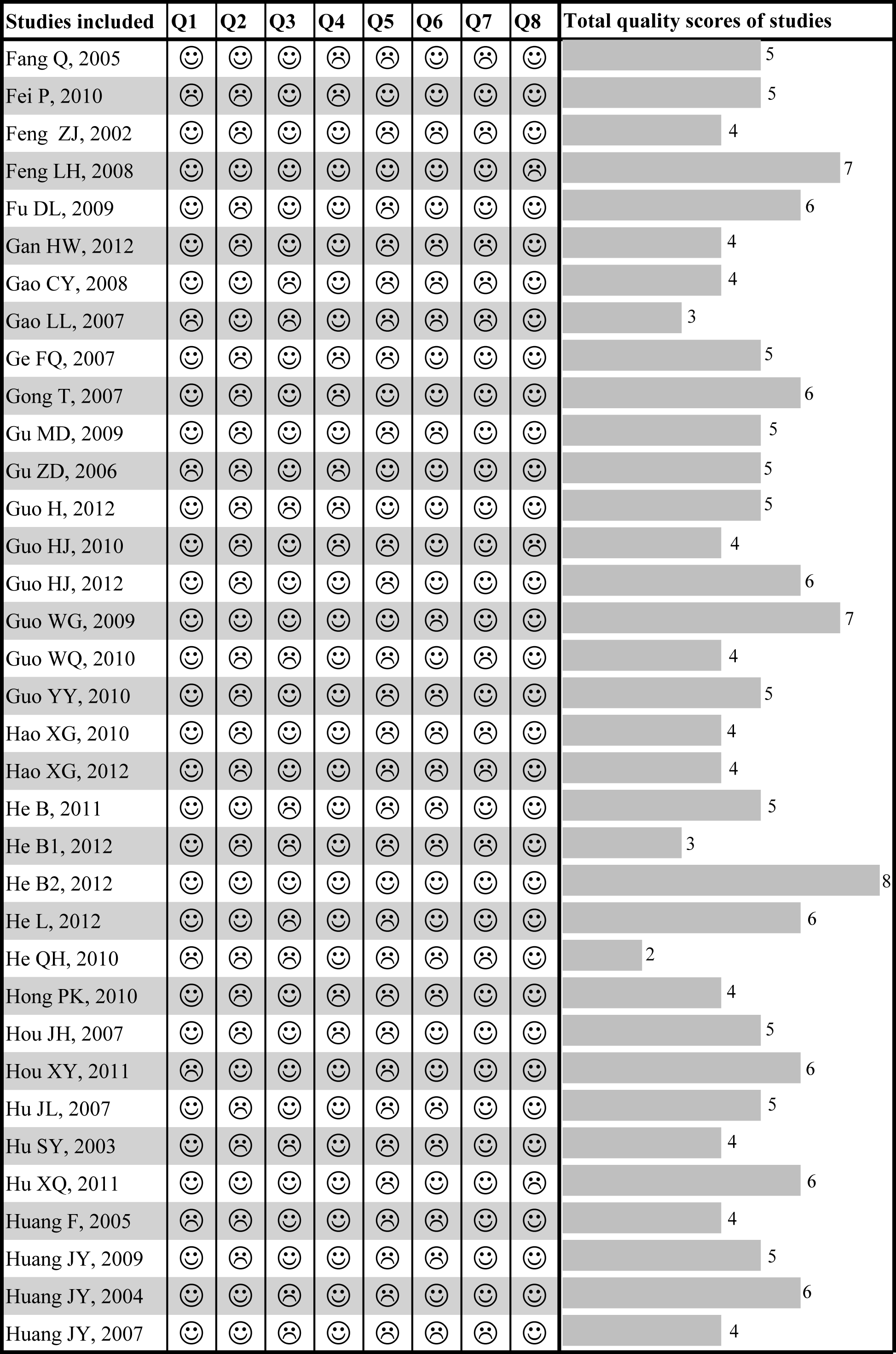


**Figure S2 Total score of quality assessment for meta-analysis (Cont’d)**


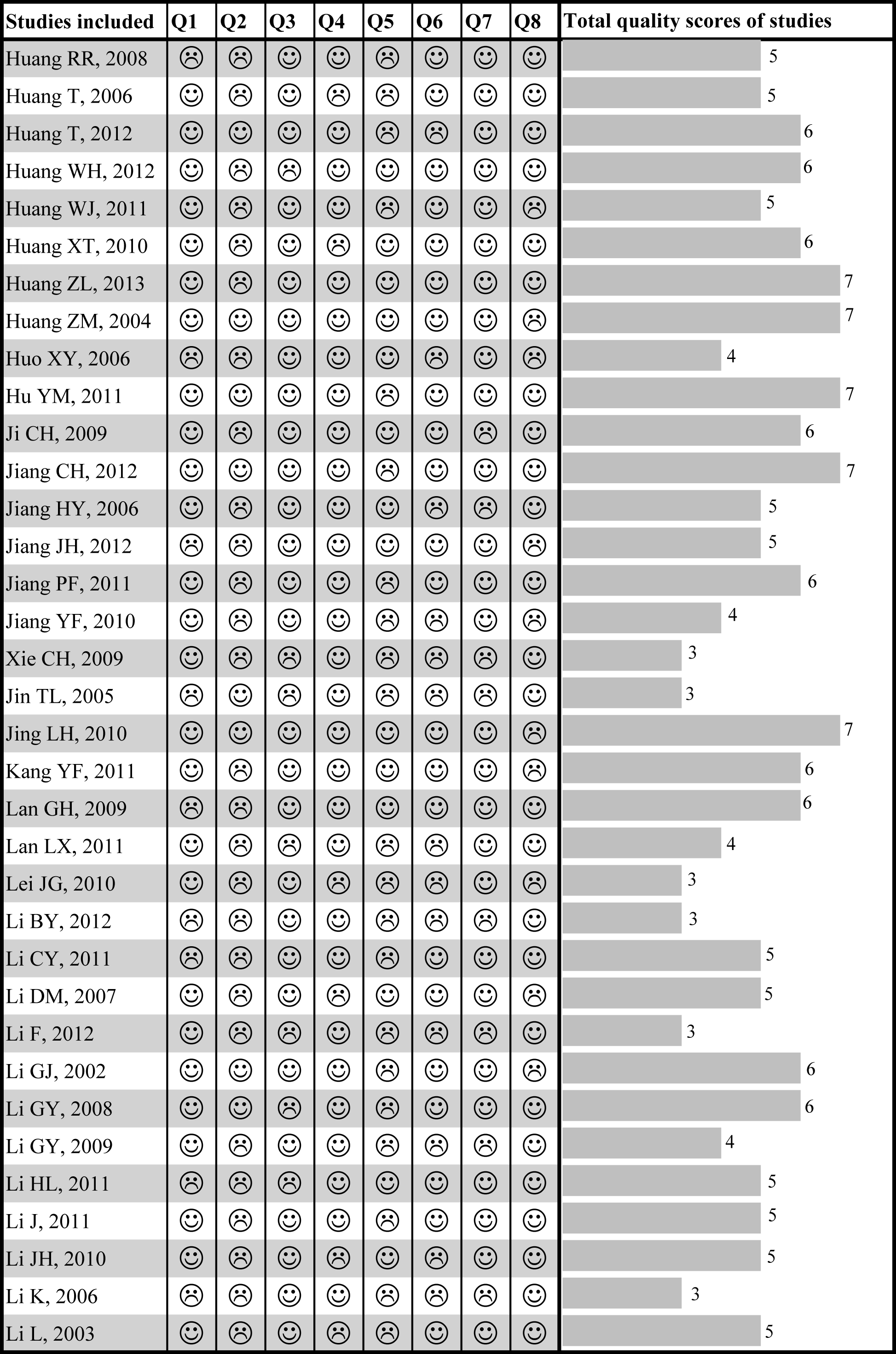


**Figure S2 Total score of quality assessment for meta-analysis (Cont’d)**


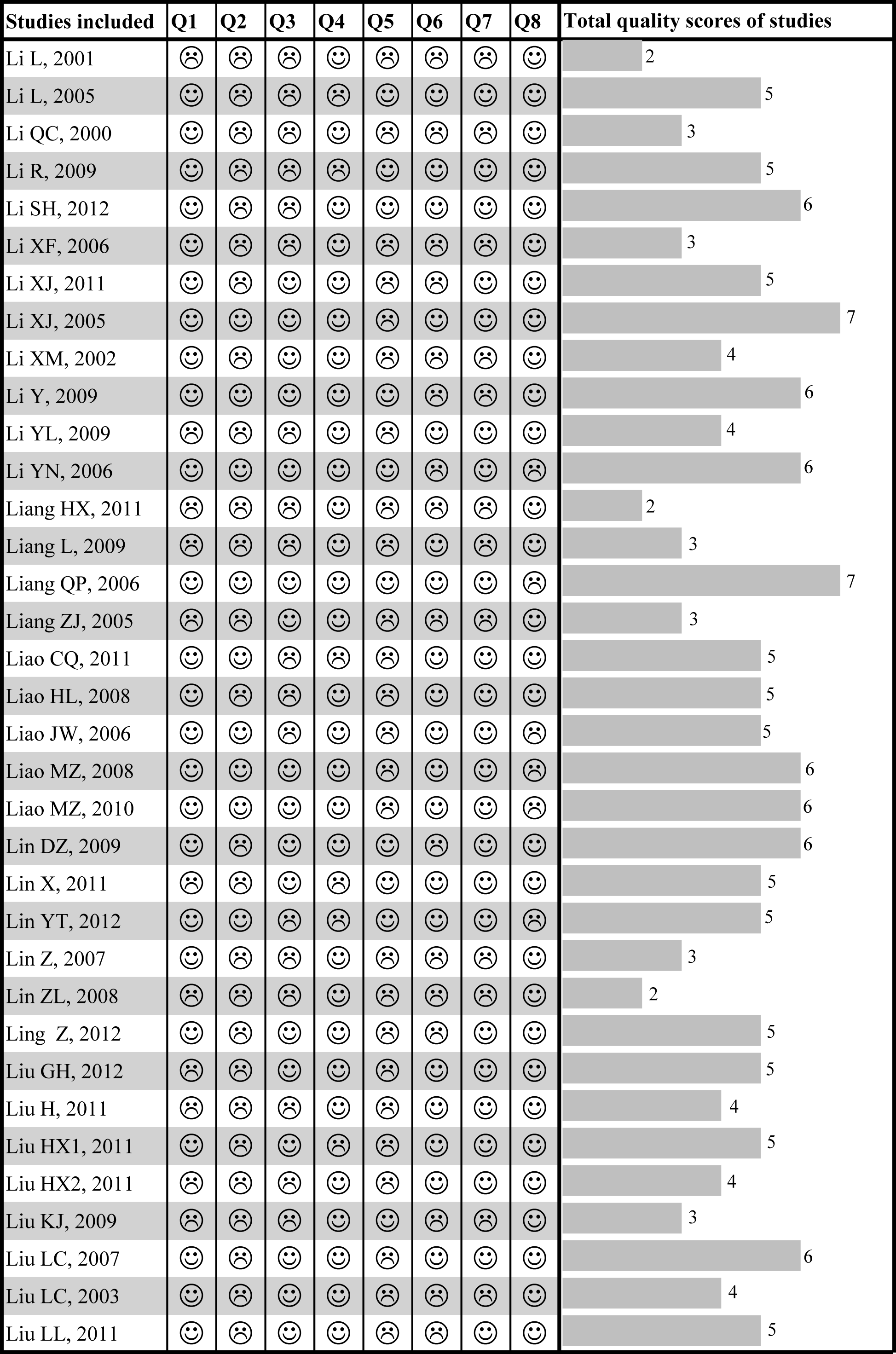


**Figure S2 Total score of quality assessment for meta-analysis (Cont’d)**


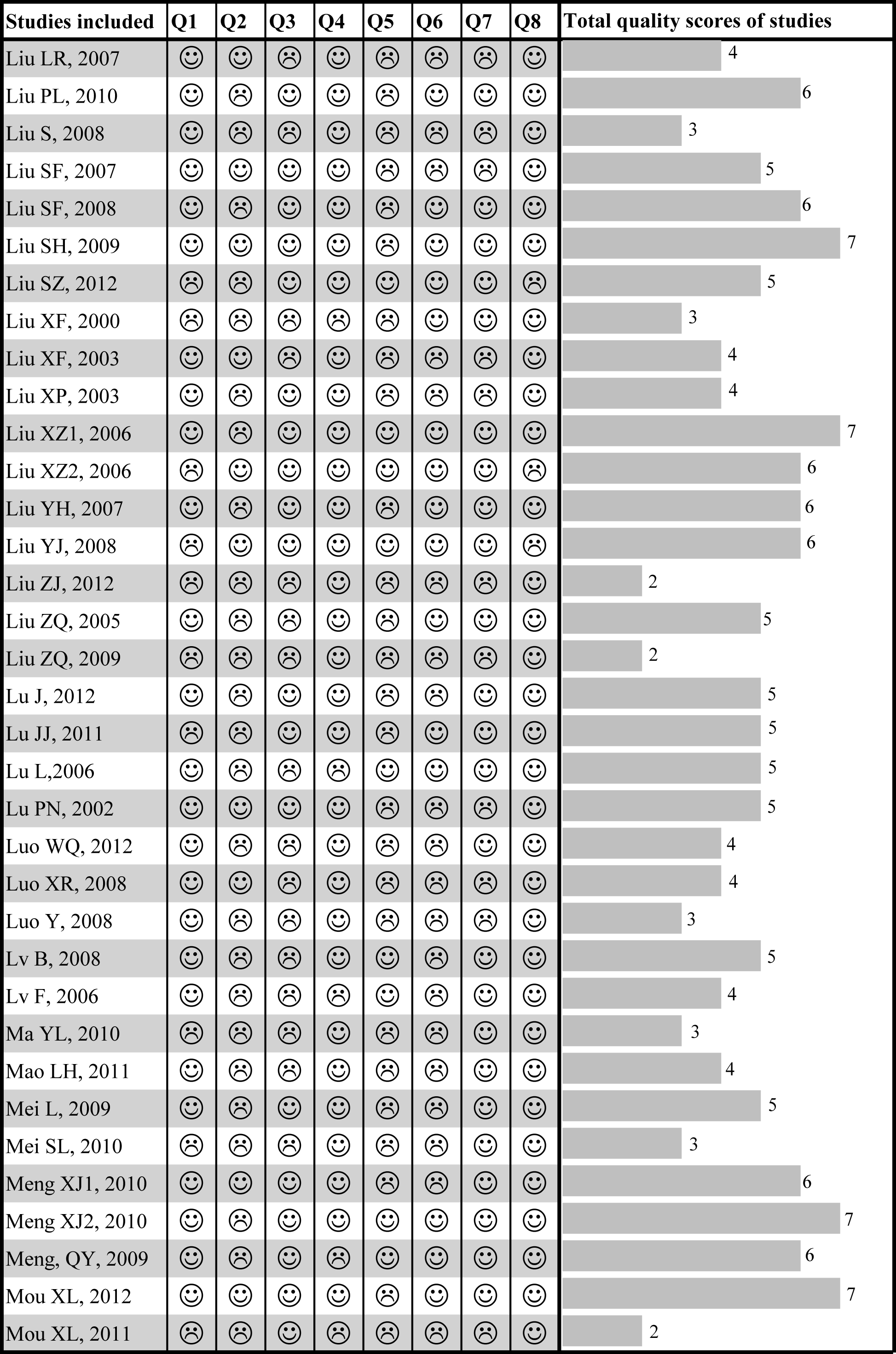


**Figure S2 Total score of quality assessment for meta-analysis (Cont’d)**


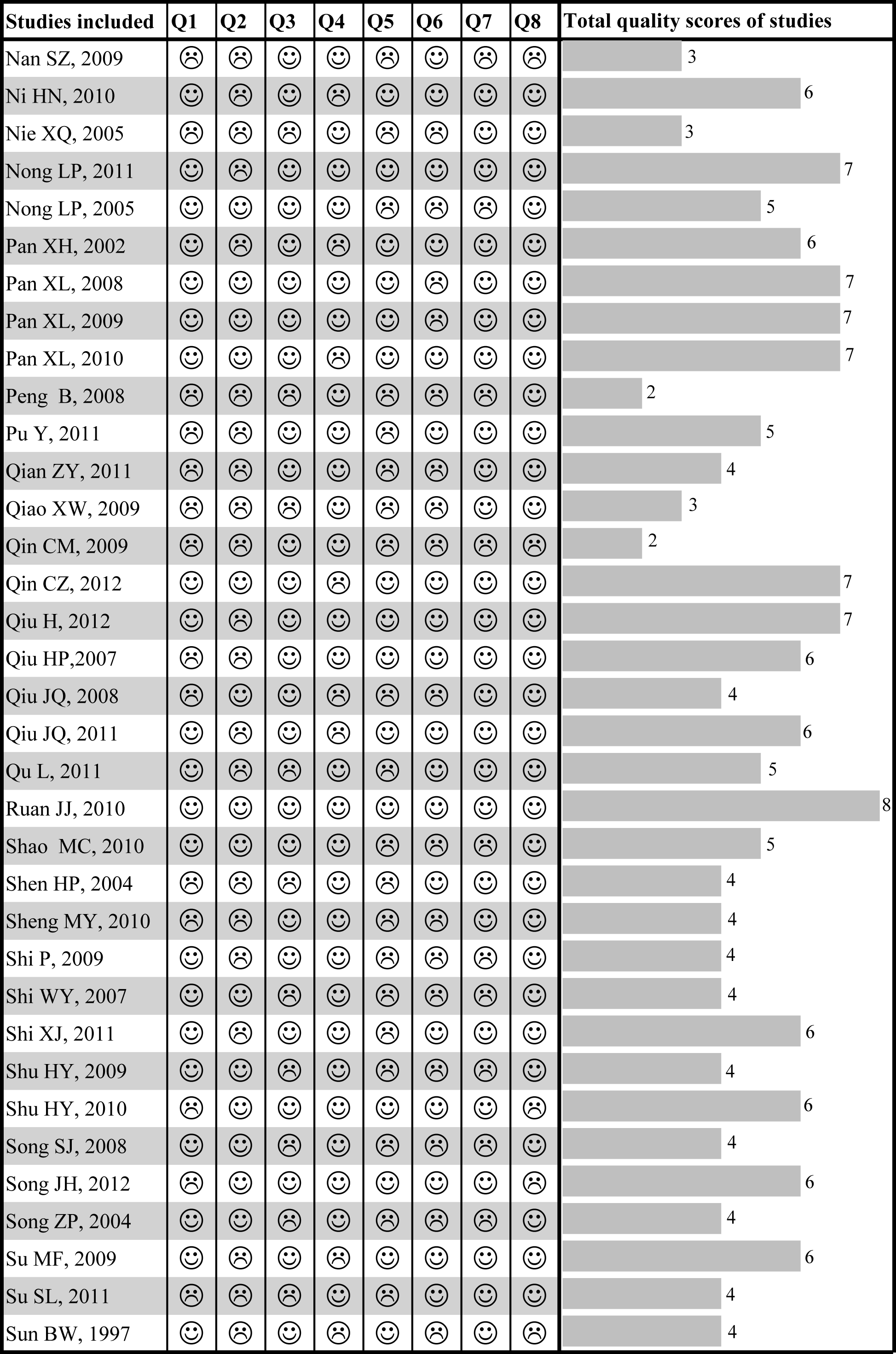


**Figure S2 Total score of quality assessment for meta-analysis (Cont’d)**


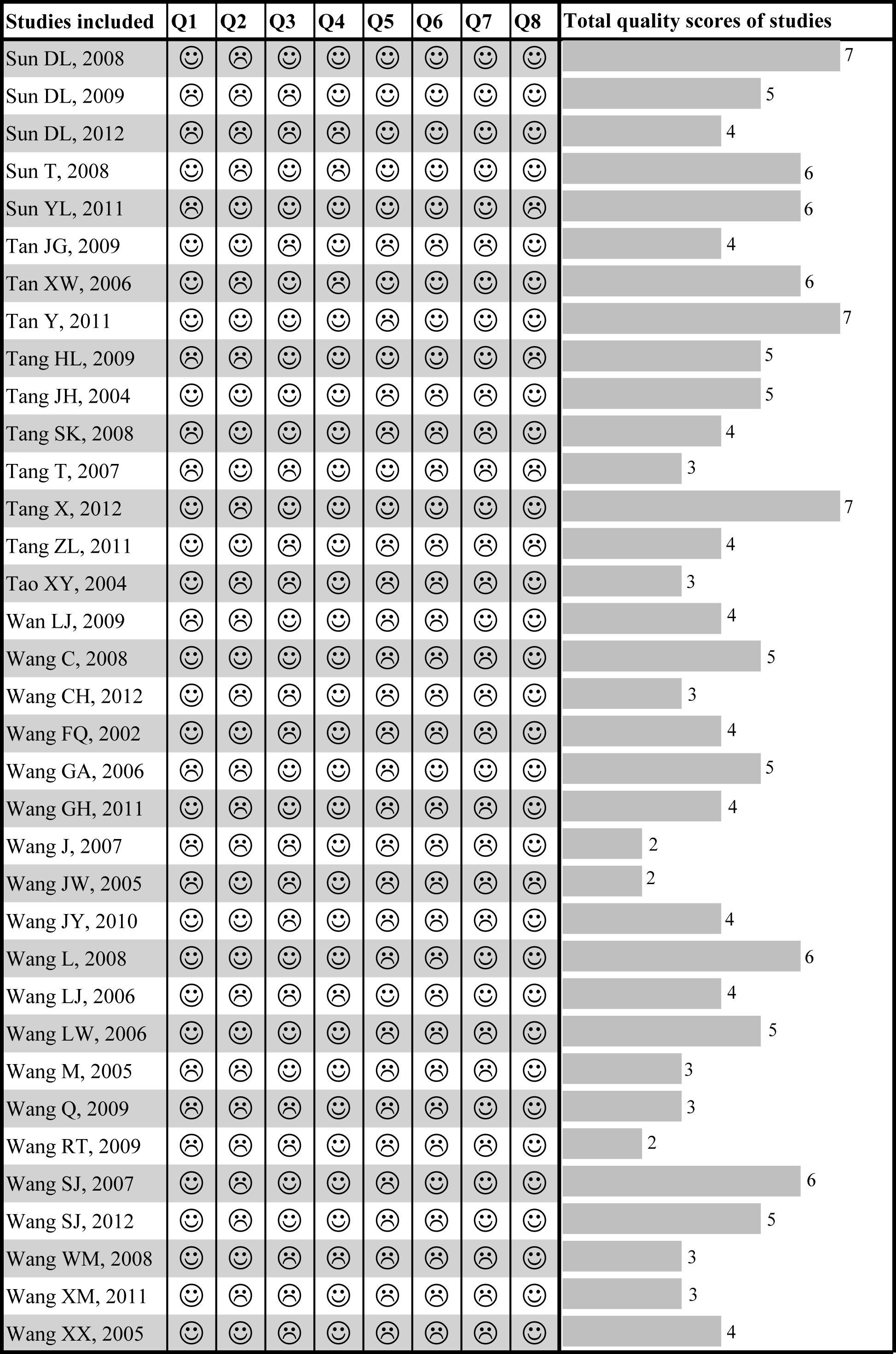


**Table S1 Total score of quality assessment for meta-analysis (Cont’d)**


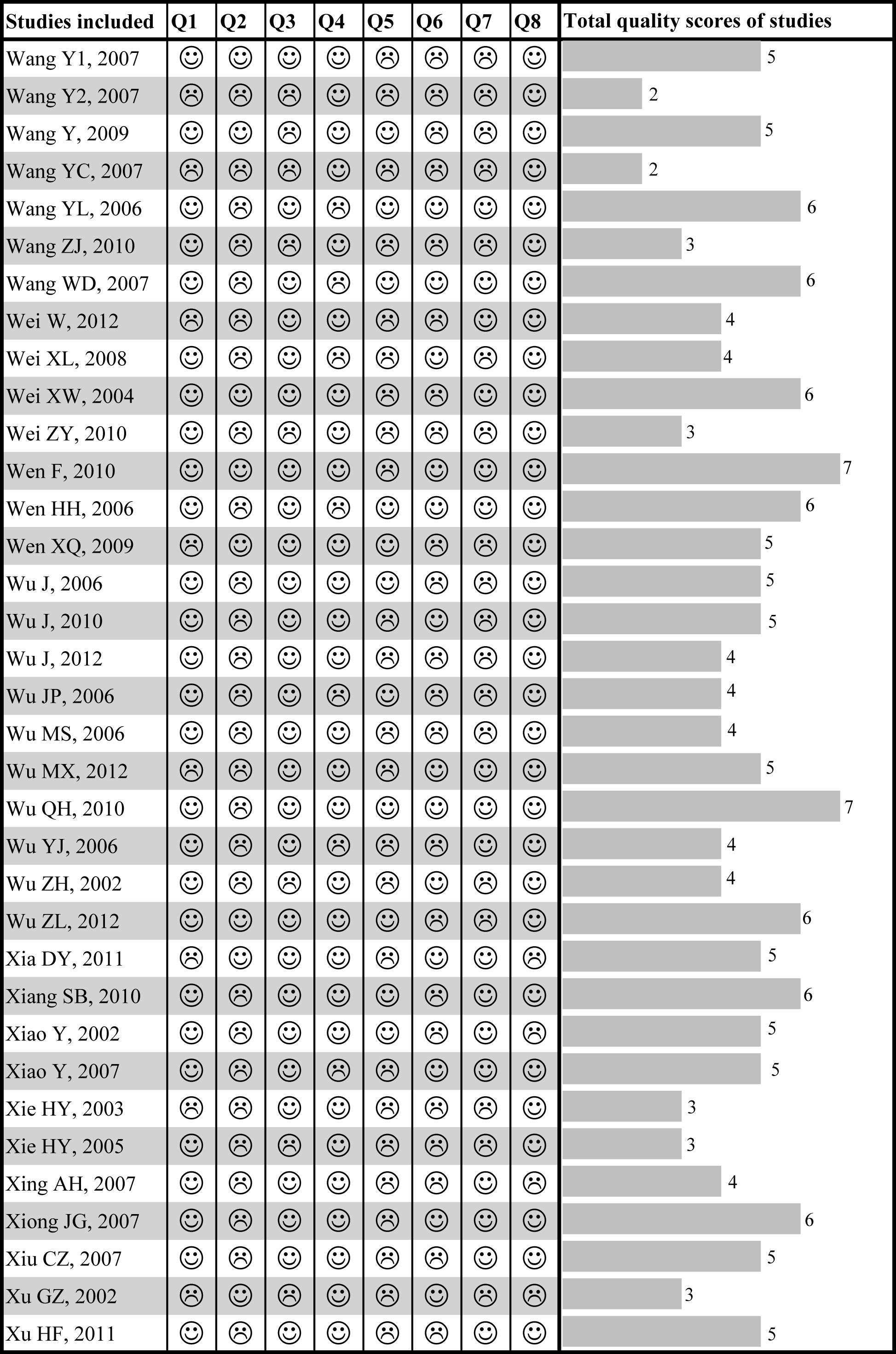


**Figure S2 Total score of quality assessment for meta-analysis (Cont’d)**


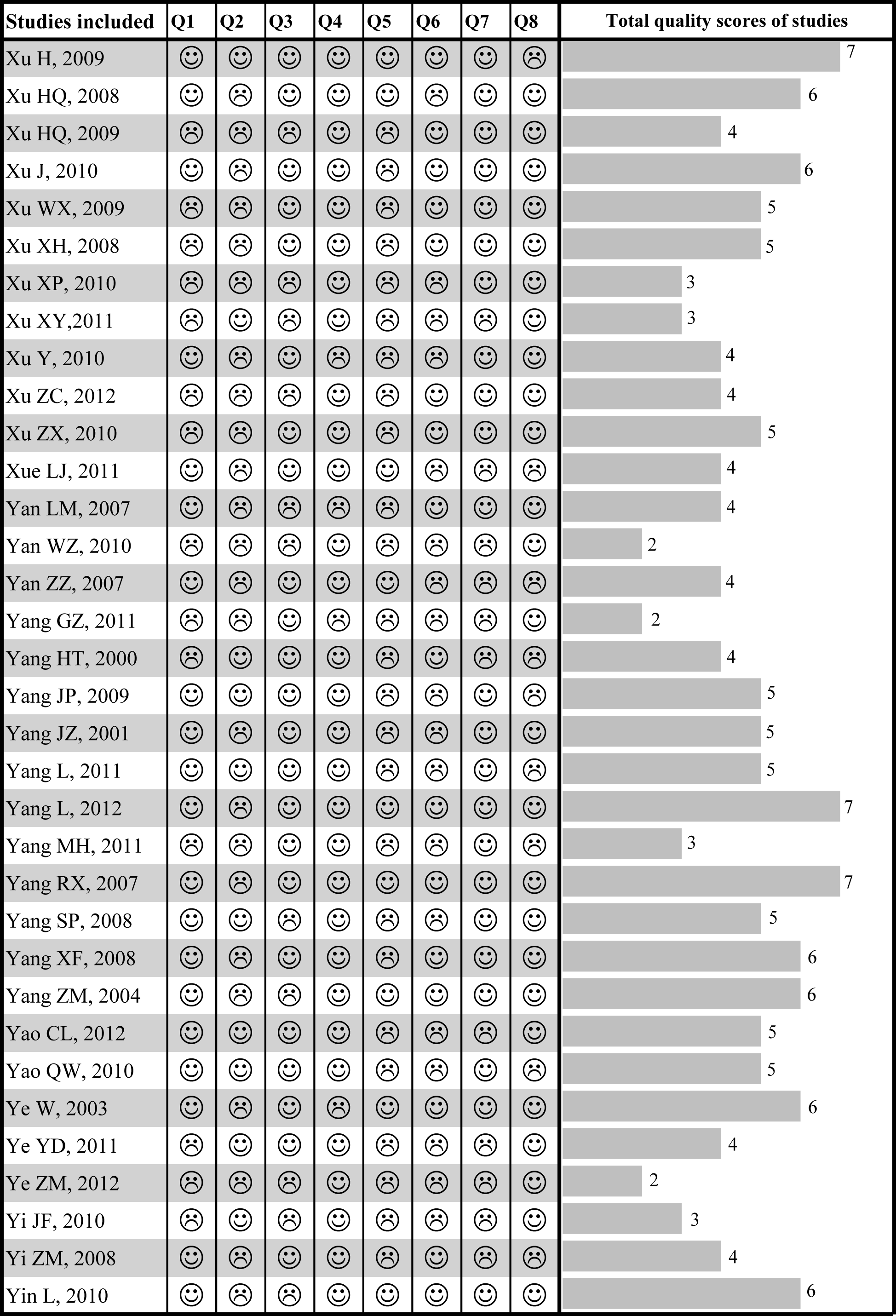


**Figure S2 Total score of quality assessment for meta-analysis (Cont’d)**


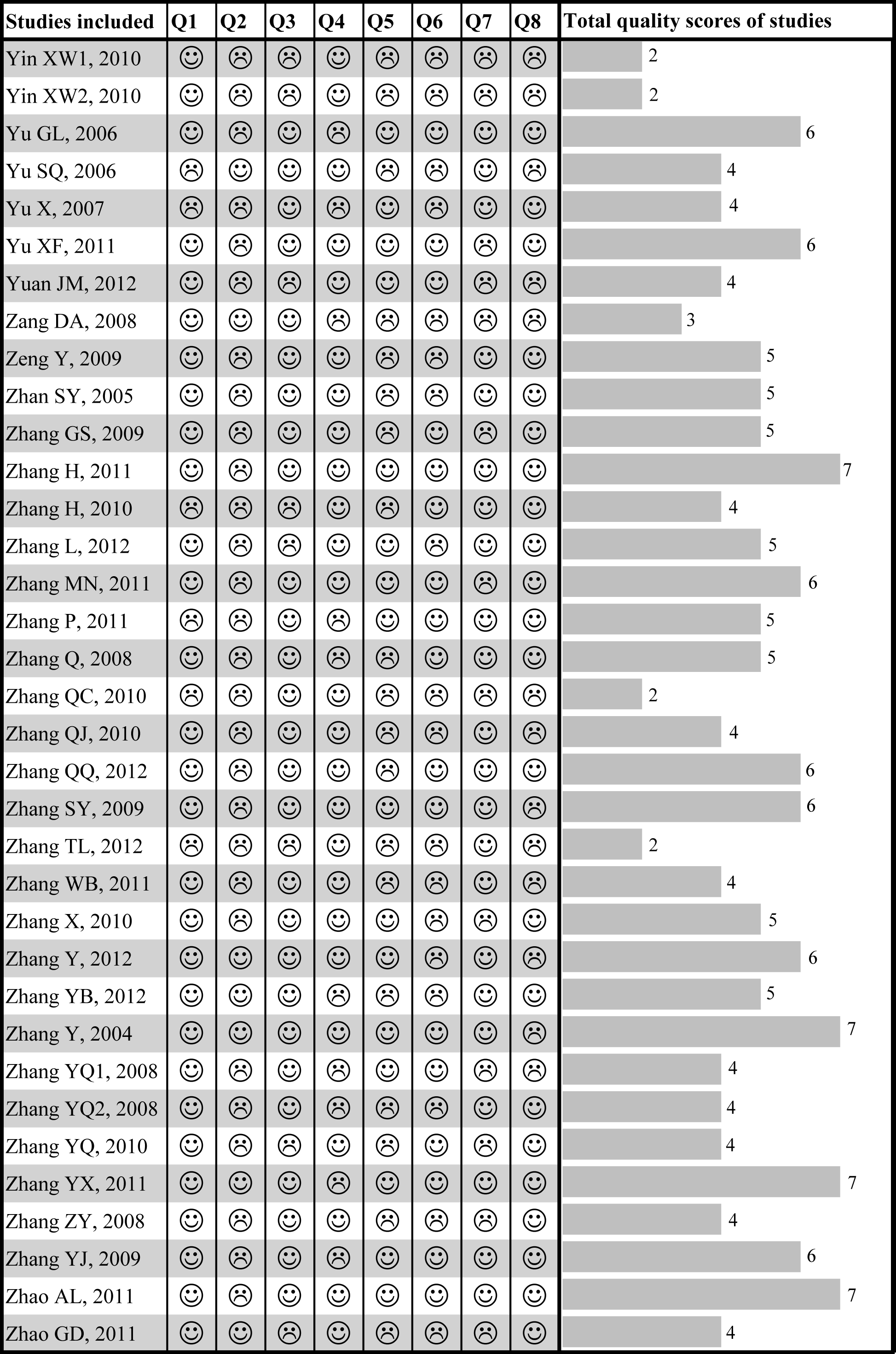


**Figure S2 Total score of quality assessment for meta-analysis (Cont’d)**


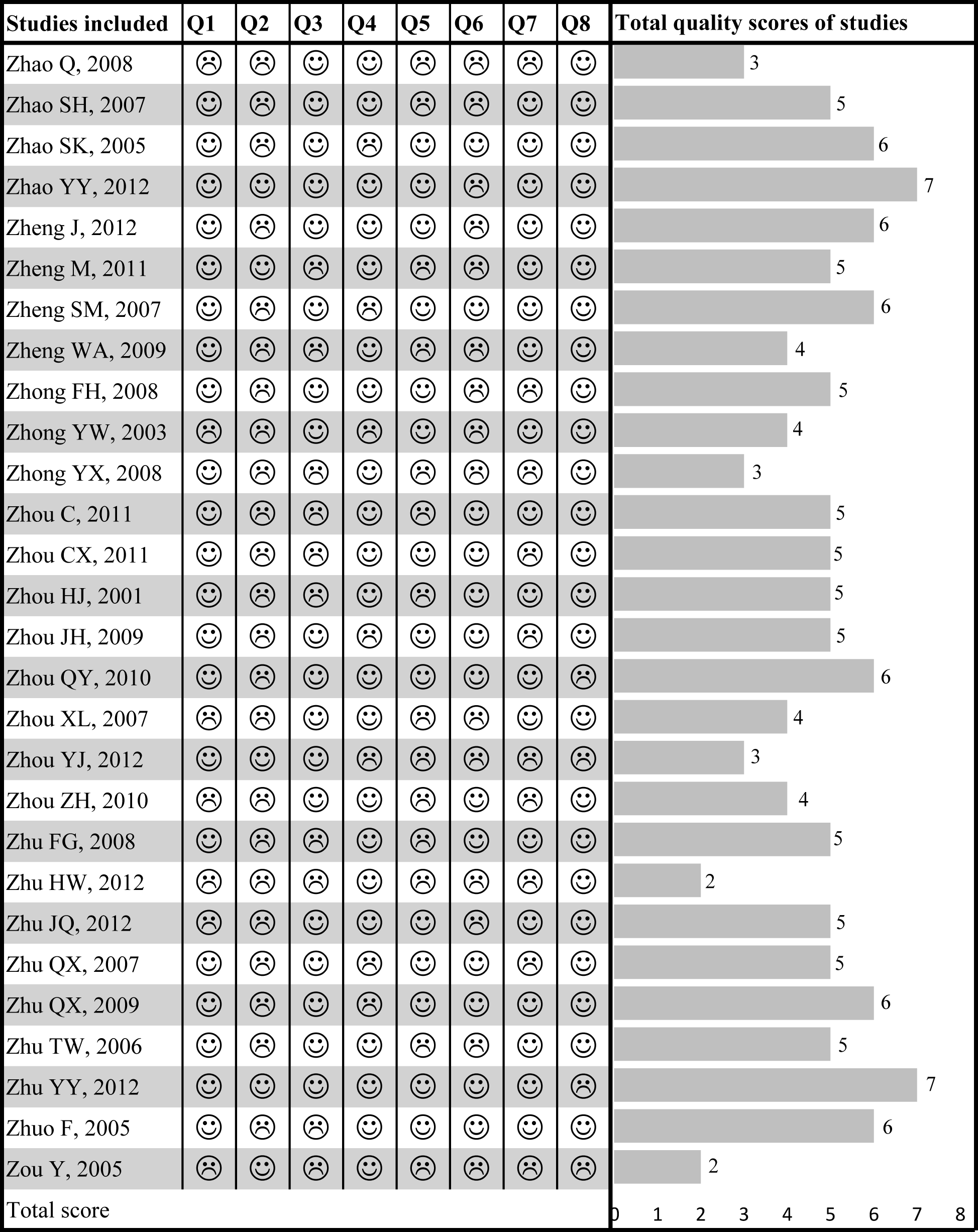


*The smile faces in the figure means the article match the criteria in table S1; the cry faces means the article dose not match the criteria in Table S1 for each question.

**Table S4a nonparametric test and ANOVA test for combinations of HIV** tests

|  |  |  | **Nonparametric** | |  | **ANOVA** | |
| --- | --- | --- | --- | --- | --- | --- | --- |
| **Group** | **N** | **Median (%)** | ***χ^2^*** | ***P*** |  | ***F*** | ***P*** |
| ELISA+WB | 40 | 0.00 | 0.859 | 0.651 |  | 0.622 | 0.539 |
| ELISA+ ELISA | 19 | 0.00 |  |  |  |  |  |
| Unspecified | 46 | 0.00 |  |  |  |  |  |
| Total | 105 | 0.00 |  |  |  |  |  |

Unspecified include: unspecified+ELISA, unspecified+WB, unspecified+ unspecified , ELISA+ unspecified

**Table S4b nonparametric test and ANOVA test for combinations of syphilis tests**

|  |  |  |  | **Nonparametric** | |  | **ANOVA** | |
| --- | --- | --- | --- | --- | --- | --- | --- | --- |
| **Screen** | **Confirm** | **N** | **Median (%)** | ***χ^2^*** | ***P*** |  | ***F*** | ***P*** |
| Troponemal | Troponemal | 47 | 0.48 | 3.109 | 0.375 |  | 0.020 | 0.996 |
| Troponemal | Nontroponemal | 13 | 0.78 |  |  |  |  |  |
| Non-troponemal | Tropomemal | 35 | 1.52 |  |  |  |  |  |
| Unspecified |  | 71 | 1.11 |  |  |  |  |  |
| Total |  | 166 | 0.92 |  |  |  |  |  |

Troponemal test include: ELISA, TPHA, FTA, CMIA, TPPA, TRUST, TPI, Abbott Determine syphilis test

Nontroponemal test include: VDRL, RPR, USR

Unspecified include: ELISA+unspecified, RPR+unspecified, TPPA+unspecified, TRUST+unspecified, unspecified+ELISA, unspecified+RPR, unspecified+TPHA, unspecified+unspecified

**Table S4c nonparametric test and ANOVA test for combinations of gonorrhea tests**

|  |  |  | **Nonparametric** | |  | **ANOVA** | |
| --- | --- | --- | --- | --- | --- | --- | --- |
| **Group** | **N** | **Median (%)** | ***χ^2^*** | ***P*** |  | ***F*** | ***P*** |
| Smear test | 3 | 1.35 | 1.620 | 0.805 |  | 0.569 | 0.691 |
| Antigen test | 1 | 0. 80 |  |  |  |  |  |
| Culture | 2 | 6.53 |  |  |  |  |  |
| Nucleic Acid Test | 6 | 0.63 |  |  |  |  |  |
| Unspecified | 3 | 3.12 |  |  |  |  |  |
| Total | 15 | 1.35 |  |  |  |  |  |

Smear test include: papanicolaou stain, gram staining

Antigen test include: colloid gold-labeled immune spot filtration method

Culture include: oxidase, glucolysis test, standard culture procedure

Nucleic Acid Test include: Ligase Chain Reaction, PCR, In vivo multiplex diagnostic test

Unspecified include: unspecified+ELISA, unspecified+RPR, unspecified+TPHA, unspecified+ unspecified, RPR+ unspecified, TPPA+ unspecified, ELISA+ unspecified

**Table S4d nonparametric test and ANOVA test for combinations of chlamydia tests**

|  |  |  | **Nonparametric** | |  | **ANOVA** | |
| --- | --- | --- | --- | --- | --- | --- | --- |
| **Group** | **N** | **Median (%)** | ***χ^2^*** | ***P*** |  | ***F*** | ***P*** |
| Antigen test | 2 | 8.58 | 1.149 | 0.563 |  | 0.230 | 0.800 |
| Nucleic Acid Test | 5 | 3.55 |  |  |  |  |  |
| Unspecified | 3 | 3.37 |  |  |  |  |  |
| Total | 10 | 3.55 |  |  |  |  |  |

Antigen test include: Colloidal gold-labeled immune spot filtration method, Rapid antigen test

Nucleic Acid Test include: Ligase Chain Reaction, PCR, In vivo multiplex diagnostic test

Unspecified: the testing method is unknown

**Table S4e nonparametric test and ANOVA test for combinations of HPV tests**

|  |  |  | **Nonparametric** | |  | **ANOVA** | |
| --- | --- | --- | --- | --- | --- | --- | --- |
| **Group** | **N** | **Median (%)** | ***χ^2^*** | ***P*** |  | ***F*** | ***P*** |
| Gene | 2 | 38.38 | 0.300 | 0.861 |  | 0.119 | 0.899 |
| PCR | 1 | 16.36 |  |  |  |  |  |
| Unspecified | 1 | 17.00 |  |  |  |  |  |
| Total | 4 | 16.68 |  |  |  |  |  |

**Table S4f nonparametric test and ANOVA test for combinations of HSV tests**

| **Screen** | **Confirm** | **N** | **Median (%)** | **Nonparametric** | |  | **ANOVA** | |
| --- | --- | --- | --- | --- | --- | --- | --- | --- |
|  |  |  |  | ***χ^2^*** | ***P*** |  | ***F*** | ***P*** |
| ELISA | ELISA | 1 | 14.67 | 1.434 | 0.488 |  | 0.229 | 0.800 |
| ELISA | WB | 1 | 26.48 |  |  |  |  |  |
| Unspecified |  | 9 | 4.40 |  |  |  |  |  |
| Total |  | 11 | 5.47 |  |  |  |  |  |

Unspecified include: ELISA+unspecified, unspecified+unspecified

**Table S4g nonparametric test and ANOVA test for combinations of HCV tests**

| **Screen** | **N** | **Median (%)** | **Nonparametric** | |  | **ANOVA** | |
| --- | --- | --- | --- | --- | --- | --- | --- |
|  |  |  | ***Z*** | ***P*** |  | ***F*** | ***P*** |
| ELISA | 48 | 0.31 | -1.507 | 0.132 |  | 1.321 | 0.254 |
| Unspecified | 24 | 0. 45 |  |  |  |  |  |
| Total | 72 | 0.38 |  |  |  |  |  |

**Table S4h nonparametric test and ANOVA test for combinations of HBV tests**

| **Screen** | **N** | **Median (%)** | **Nonparametric** | |  | **ANOVA** | |
| --- | --- | --- | --- | --- | --- | --- | --- |
|  |  |  | ***Z*** | ***P*** |  | ***F*** | ***P*** |
| Specified | 49 | 7.36 | 2.182 | 0.140 |  | 1.254 | 0.267 |
| Unspecified | 16 | 12.18 |  |  |  |  |  |
| Total | 65 | 8.09 |  |  |  |  |  |

Specified: chemiluminescence immunoassay ( N=4) , EIA (N=2), ELISA (N=43)

Unspecified: the tests was not specified

**Checklist S1**. PRISMA Checklist

| **Section/**  **topic** | **#** | **Checklist item** | **Reported section #** |
| --- | --- | --- | --- |
| **TITLE** | | |  |
| Title | 1 | Identify the report as a systematic review, meta-analysis, or both. | Title |
| **ABSTRACT** | | |  |
| Structured summary | 2 | Provide a structured summary including, as applicable: background; objectives; data sources; study eligibility criteria, participants, and interventions; study appraisal and synthesis methods; results; limitations; conclusions and implications of key findings; systematic review registration number. | Abstract |
| **INTRODUCTION** | | |  |
| Rationale | 3 | Describe the rationale for the review in the context of what is already known. | Introduction – 1^st^ to 2^nd^ paragraphs |
| Objectives | 4 | Provide an explicit statement of questions being addressed with reference to participants, interventions, comparisons, outcomes, and study design (PICOS). | Introduction – 3^rd^ paragraph |
| **METHODS** | | |  |
| Protocol and registration | 5 | Indicate if a review protocol exists, if and where it can be accessed (e.g., Web address), and, if available, provide registration information including registration number. | Method –Conduct of Systematic Review and Meta-Analysis |
| Eligibility criteria | 6 | Specify study characteristics (e.g., PICOS, length of follow-up) and report characteristics (e.g., years considered, language, publication status) used as criteria for eligibility, giving rationale. | Method – Selection criteria for considering studies for this review |
| Information sources | 7 | Describe all information sources (e.g., databases with dates of coverage, contact with study authors to identify additional studies) in the search and date last searched. | Method – Search strategy for identification of studies |
| Search | 8 | Present full electronic search strategy for at least one database, including any limits used, such that it could be repeated. | Method – Search strategy for identification of studies |
| Study selection | 9 | State the process for selecting studies (i.e., screening, eligibility, included in systematic review, and, if applicable, included in the meta-analysis). | Method – Selection Criteria for considering studies for this review |
| Data collection process | 10 | Describe method of data extraction from reports (e.g., piloted forms, independently, in duplicate) and any processes for obtaining and confirming data from investigators. | Method – Data Extraction and Management |
| Data items | 11 | List and define all variables for which data were sought (e.g., PICOS, funding sources) and any assumptions and simplifications made. | Method – Definition of Outcomes in Data Extraction |
| Risk of bias in individual studies | 12 | Describe methods used for assessing risk of bias of individual studies (including specification of whether this was done at the study or outcome level), and how this information is to be used in any data synthesis. | Method –Quality Assessment  Table S1 |
| Summary measures | 13 | State the principal summary measures (e.g., risk ratio, difference in means). | Method – Definition of Outcomes |
| Synthesis of results | 14 | Describe the methods of handling data and combining results of studies, if done, including measures of consistency (e.g., I^2^) for each meta-analysis. | Method – Statistical analysis |
| Risk of bias across studies | 15 | Specify any assessment of risk of bias that may affect the cumulative evidence (e.g., publication bias, selective reporting within studies). | Method – Statistical analysis |
| Additional analyses | 16 | Describe methods of additional analyses (e.g., sensitivity or subgroup analyses, meta-regression), if done, indicating which were pre-specified. | Method – Statistical analysis |
| **RESULTS** | | |  |
| Study selection | 17 | Give numbers of studies screened, assessed for eligibility, and included in the review, with reasons for exclusions at each stage, ideally with a flow diagram. | Figure 1 |
| Study characteristics | 18 | For each study, present characteristics for which data were extracted (e.g., study size, PICOS, follow-up period) and provide the citations. | Results - Characteristics of Included Studies  Table S2 |
| Risk of bias within studies | 19 | Present data on risk of bias of each study and, if available, any outcome level assessment (see item 12). | Results - Risk of bias within and across studies Table 1 and Figure S1 |
| Results of individual studies | 20 | For all outcomes considered (benefits or harms), present, for each study: (a) simple summary data for each intervention group (b) effect estimates and confidence intervals, ideally with a forest plot. | Results, Table 1 and Figure 1 |
| Synthesis of results | 21 | Present results of each meta-analysis done, including confidence intervals and measures of consistency. | Table 1 and Figure S1 |
| Risk of bias across studies | 22 | Present results of any assessment of risk of bias across studies (see Item 15). | Results, Table 1, Figure S1 |
| Additional analysis | 23 | Give results of additional analyses, if done (e.g., sensitivity or subgroup analyses, meta-regression [see Item 16]). | Table 1 |
| **DISCUSSION** | | |  |
| Summary of evidence | 24 | Summarize the main findings including the strength of evidence for each main outcome; consider their relevance to key groups (e.g., healthcare providers, users, and policy makers). | Discussion – 1^st^ to 4^th^ paragraphs |
| Limitations | 25 | Discuss limitations at study and outcome level (e.g., risk of bias), and at review-level (e.g., incomplete retrieval of identified research, reporting bias). | Discussion – 5^h^ paragraph |
| Conclusions | 26 | Provide a general interpretation of the results in the context of other evidence, and implications for future research. | Discussion – 6^h^ paragraph |
| **FUNDING** | | |  |
| Funding | 27 | Describe sources of funding for the systematic review and other support (e.g., supply of data); role of funders for the systematic review. | Source of support |

| **Table S5a Characteristics description of studies included in meta-analysis of HIV prevalence** | | | | | | |  |  |  |
| --- | --- | --- | --- | --- | --- | --- | --- | --- | --- |
| **First Author &** | **Study** | **Study Location** |  | **Subgroup** | **Sample** | **Presumptive** | **Confirmatory** | **No. of** | **No. of people** |
| **Published Year** | **Period** | **City/county** | **Province** | **Population** | **Size** | **test(s)** | **test(s)*** | **infection** | **tested** |
| HIV |  |  |  |  |  |  |  |  |  |
| Ding W, 2011[^1^](#_ENREF_1) | 2006-2009 | Qingzhou | Shandong | Migrant women | 3386 | ELISA | WB | 12 | 3386 |
| Ding ZW, 2010[^2^](#_ENREF_2) | 2008 | Jilin | Jilin | Migrant women | 1530 | ELISA | WB | 2 | 838 |
| Huo XY, 2006[^3^](#_ENREF_3) | 1992-2005 | Weifang | Shandong | Migrant women | 6092 | ELISA | WB | 39 | 6092 |
| Li JH, 2010[^4^](#_ENREF_4) | 2009 | Tonghua | Jilin | Migrant women | 892 | ELISA | — | 5 | 892 |
| Li YL, 2009[^5^](#_ENREF_5) | 2005-2006 | Tai'an | Shandong | Migrant women | 2224 | ELISA-1/ELISA-2 | WB | 11 | 2224 |
| Liu H, 2011[^6^](#_ENREF_6) | — | Zhoushan | Zhejiang | Migrant women | 518 | YES | YES | 2 | 518 |
| Liu KJ, 2009[^7^](#_ENREF_7) | 2006-2008 | Zoucheng | Shandong | Migrant women | 2698 | ELISA | ELISA | 8 | 2698 |
| Liu YJ, 2008[^8^](#_ENREF_8) | 2006-2007 | Tai'an | Shandong | Migrant women | 1615 | ELISA | WB | 10 | 1615 |
| Lv F, 2006a[^9^](#_ENREF_9) | 2003-2006 | Shandong | Shandong | Migrant women | 37829 | — | — | 244 | 37829 |
| Lv F, 2006b[^9^](#_ENREF_9) | 2005-2006 | Shanxi | Shanxi | Migrant women | 2881 | — | — | 33 | 2881 |
| Mei SL, 2010[^10^](#_ENREF_10) | 2007-2009 | Lishui | Zhejiang | Migrant women | 9560 | ELISA | WB | 15 | 9560 |
| Nan SZ, 2009[^11^](#_ENREF_11) | 2006 | Antu | Jilin | Migrant women | 434 | ELISA | YES | 9 | 434 |
| Ni HN, 2010[^12^](#_ENREF_12) | 2008 | Xianju | Zhejiang | Migrant women | 1033 | ELISA | WB | 5 | 1033 |
| Shen HP, 2004[^13^](#_ENREF_13) | 2001-2002 | Nanhui | Shanghai | Migrant women | 958 | ELISA | YES | 0 | 958 |
| Shu HY, 2010[^14^](#_ENREF_14) | 2005-2008 | Zoucheng | Shandong | Migrant women | 3091 | ELISA | ELISA/WB | 11 | 3091 |
| Su SL, 2011[^15^](#_ENREF_15) | 2008 | Shandong | Shandong | Migrant women | 7102 | ELISA | WB | 46 | 7102 |
| Sun DL, 2009[^16^](#_ENREF_16) | 2006-2007 | Lianyungang | Jiangsu | Migrant women | 7423 | ELISA-1,ELISA-2 | YES | 10 | 3562 |
| Sun DL, 2012[^17^](#_ENREF_17) | 2006-2009 | Lianyungang | Jiangsu | Migrant women | 8833 | ELISA-1,ELISA-2 | YES | 21 | 8833 |
| Wang GA, 2006[^18^](#_ENREF_18) | 2004-2005 | Weifang | Shandong | Migrant women | 6053 | ELISA | WB | 45 | 6053 |
| Wang Q, 2010[^19^](#_ENREF_19) | 2009 | Qidong | Hunan | Migrant women | 2709 | YES | YES | 2 | 95 |
| Wu MX, 2012[^20^](#_ENREF_20) | 1996-2010 | Wuyi | Zhejiang | Migrant women | 1507 | ELISA | WB | 5 | 1507 |
| Wu YJ, 2006[^21^](#_ENREF_21) | — | Jinxiang | Jiangsu | Migrant women | 446 | ELISA | YES | 0 | 446 |
| Xiu CZ, 2007[^22^](#_ENREF_22) | 2005 | Qingdao | Shandong | Migrant women | 1854 | ELISA | WB | 15 | 1854 |
| Xu HQ, 2009[^23^](#_ENREF_23) | 2007 | Jiaxin | Zhejiang | Migrant women | 899 | ELISA | WB | 0 | 896 |
| **Table S5a Characteristics description of studies included in meta-analysis of HIV prevalence (Cont’d)** | | | | | | |  |  |  |
| **First Author &** | **Study** | **Study Location** |  | **Subgroup** | **Sample** | **Presumptive** | **Confirmatory** | **No. of** | **No. of people** |
| **Published Year** | **Period** | **City/county** | **Province** | **Population** | **Size** | **test(s)** | **test(s)*** | **infection** | **tested** |
| HIV(Cont’d) |  |  |  |  |  |  |  |  |  |
| Xu WX, 2009[^24^](#_ENREF_24) | 2004-2007 | Jiaxing | Zhejiang | Migrant women | 404 | ELISA | WB | 0 | 791 |
| Xu XH, 2008[^25^](#_ENREF_25) | 2007 | Jinning | Zhejiang | Migrant women | 930 | YES | YES | 2 | 930 |
| Xu ZC, 2012[^26^](#_ENREF_26) | 2007-2011 | Zhejiang | Zhejiang | Migrant women | 5096 | ELISA | YES | 6 | 4240 |
| Xu ZX, 2010[^27^](#_ENREF_27) | 2000- | Xintai | Hebei | Migrant women | 478 | ELISA-1,ELISA-2 | WB | 6 | 478 |
| Yin L, 2010[^28^](#_ENREF_28) | 2005-2008 | Zhangqiu,Jinan | Shandong | Migrant women | 421 | ELISA | WB | 4 | 421 |
| Yin XW, 2010a[^29^](#_ENREF_29) | 2005-2007 | Ningyang | Shandong | Migrant women | 489 | YES | YES | 1 | 185 |
| Yin XW, 2010b[^29^](#_ENREF_29) | 2007-2009 | Ningyang | Shandong | Migrant women | 891 | YES | YES | 6 | 891 |
| Yu SQ, 2006[^30^](#_ENREF_30) | 1990-2004 | Weifang | Shandong | Migrant women | 1437 | ELISA | WB | 7 | 1437 |
| Zhang SY, 2009[^31^](#_ENREF_31) | 2007 | Ju county | Shandong | Migrant women | 742 | ELISA | WB | 1 | 742 |
| Zhang WB, 2011[^32^](#_ENREF_32) | 2009-2009 | Pingyao County | Shanxi | Migrant women | 2305 | YES | YES | 14 | 2305 |
| Zhang X, 2010[^33^](#_ENREF_33) | 2006-2008 | Chuzhou, Huai'an | Jiangsu | Migrant women | 3911 | ELISA | YES | 13 | 3911 |
| Zhang YQ, 2008[^34^](#_ENREF_34) | 2005-2007 | Zoucheng | Shandong | Migrant women | 2698 | ELISA | WB | 8 | 2698 |
| Zhang YQ, 2010[^35^](#_ENREF_35) | 2005-2009 | Zoucheng | Shandong | Migrant women | 3235 | ELISA | WB | 5 | 3235 |
| Chen XS, 2006[^36^](#_ENREF_36) | 2000 | Tongling | Anhui | Truck drivers | 550 | ELISA | WB | 0 | 550 |
| Feng LH, 2008[^37^](#_ENREF_37) | 1997-2006 | Geermu | Qinghai | Truck drivers | 4193 | ELISA | WB | 3 | 4193 |
| Guo WQ, 2010[^38^](#_ENREF_38) | 2006 | Lanzhou | Gansu | Truck drivers | 400 | — | — | 0 | 400 |
| Li L, 2001[^39^](#_ENREF_39) | 1995-2000 | Zhuzhou | Hunan | Truck drivers | 2455 | ELISA | YES | 0 | 2455 |
| Liang QP,2006[^40^](#_ENREF_40) | 1995-2004 | Enshi | Hubei | Truck drivers | 3313 | ELISA | WB | 1 | 3313 |
| Liao JW, 2006[^41^](#_ENREF_41) | 1995-2005 | Yulin | Guangxi | Truck drivers | 1354 | YES | YES | 1 | 1354 |
| Liao MZ, 2008[^42^](#_ENREF_42) | 2007 | Jinan | Shandong | Truck drivers | 377 | ELISA | ELISA | 0 | 377 |
| Liao MZ, 2010[^43^](#_ENREF_43) | 2009 | Jinan | Shandong | Truck drivers | 425 | ELISA | ELISA | 0 | 425 |
| Liu XZ , 2006[^44^](#_ENREF_44) | 2004 | Shandong | Shandong | Truck drivers | 720 | ELISA | ELISA | 0 | 720 |
| Liu XZ, 2006a[^45^](#_ENREF_45) | 2002 | Zhengzhou | Henan | Truck drivers | 500 | ELISA | WB | 0 | 500 |
| **Table S5a Characteristics description of studies included in meta-analysis of HIV prevalence (Cont’d)** | | | | | | |  |  |  |
| **First Author &** | **Study** | **Study Location** |  | **Subgroup** | **Sample** | **Presumptive** | **Confirmatory** | **No. of** | **No. of people** |
| **Published Year** | **Period** | **City/county** | **Province** | **Population** | **Size** | **test(s)** | **test(s)*** | **infection** | **tested** |
| HIV(Cont’d) |  |  |  |  |  |  |  |  |  |
| Liu XZ, 2006b[^45^](#_ENREF_45) | 2003 | Zhengzhou | Henan | Truck drivers | 761 | ELISA | WB | 1 | 761 |
| Liu XZ, 2006c[^45^](#_ENREF_45) | 2004-2006 | Zhengzhou | Henan | Truck drivers | 1193 | ELISA | WB | 4 | 1193 |
| Nong LP, 2005[^46^](#_ENREF_46) | — | — | — | Truck drivers | 368 | ELISA | YES | 0 | 368 |
| Wang YC, 2007[^47^](#_ENREF_47) | 2006 | Dangyang | Hubei | Truck drivers | 305 | — | — | 0 | 305 |
| Wu ZH, 2002[^48^](#_ENREF_48) | 1995-2001 | Huaihua | Hunan | Truck drivers | 1200 | — | — | 0 | 1200 |
| Xie HY, 2003[^49^](#_ENREF_49) | — | Quanzhou | Fujian | Truck drivers | 76 | ELISA | WB | 0 | 76 |
| Yang HT, 2000[^50^](#_ENREF_50) | 1995-1999 | Chengzhou, Changshu | Jiangsu | Truck drivers | 200 | — | — | 0 | 200 |
| Yang JP, 2009a[^51^](#_ENREF_51) | 2005 | Anqing | Anhui | Truck drivers | 94 | ELISA | WB | 0 | 94 |
| Yang JP, 2009b[^51^](#_ENREF_51) | 2006 | Anqing | Anhui | Truck drivers | 411 | ELISA | WB | 0 | 411 |
| Yang JP, 2009c[^51^](#_ENREF_51) | 2007 | Anqing | Anhui | Truck drivers | 423 | ELISA | WB | 0 | 423 |
| Yi JF, 2010[^52^](#_ENREF_52) | 2009 | Yichun | Jiangsu | Truck drivers | 400 | — | — | 0 | 400 |
| Zhang TL, 2012[^53^](#_ENREF_53) | — | Shandong | Shandong | Truck drivers | 326 | ELISA-1 | ELISA-2 | 0 | 326 |
| Zhang Y, 2004[^54^](#_ENREF_54) | 2003 | Hubei | Hubei | Truck drivers | 2309 | ELISA | WB | 0 | 2309 |
| Ge FQ, 2007[^55^](#_ENREF_55) | 2005 | Sichuan | Sichuan | Construction workers | 599 | — | — | 0 | 571 |
| He B,2012[^56^](#_ENREF_56) | 2011 | Pinxiang | Guangxi | Construction workers | 405 | ELISA | WB | 2 | 405 |
| He L, 2012[^57^](#_ENREF_57) | 2010 | Shenzhen | Guangdong | Construction workers | 101 | ELISA | — | 0 | 101 |
| Hu XQ, 2011[^58^](#_ENREF_58) | 2010 | Baoji | Shaanxi | Construction workers | 400 | ELISA | WB | 0 | 400 |
| Liu HX, 2011[^59^](#_ENREF_59) | 2010 | Changping | Beijing | Construction workers | 449 | — | — | 0 | 449 |
| Mou XL, 2012[^60^](#_ENREF_60) | 2010 | Wuxi | Jiangsu | Construction workers | 1703 | ELISA | WB | 0 | 481 |
| Sheng MY, 2010[^61^](#_ENREF_61) | 2008 | Haining | Zhejiang | Construction workers | 231 | — | — | 0 | 231 |
| Song JH, 2012[^62^](#_ENREF_62) | 2005-2010 | Beijing | Beijing | Construction workers | 206 | ELISA | WB | 0 | 206 |
| Wang CH, 2012[^63^](#_ENREF_63) | 2011 | Huanggang | Hubei | Construction workers | 332 | ELISA |  | 0 | 301 |
| Xia DY, 2011[^64^](#_ENREF_64) | 2009 | Beijing | Beijing | Construction workers | 3161 | ELISA | WB | 0 | 3161 |
| **Table S5a Characteristics description of studies included in meta-analysis of HIV prevalence (Cont’d)** | | | | | | |  |  |  |
| **First Author &** | **Study** | **Study Location** |  | **Subgroup** | **Sample** | **Presumptive** | **Confirmatory** | **No. of** | **No. of people** |
| **Published Year** | **Period** | **City/county** | **Province** | **Population** | **Size** | **test(s)** | **test(s)*** | **infection** | **tested** |
| HIV(Cont’d) |  |  |  |  |  |  |  |  |  |
| Xu XP, 2009[^65^](#_ENREF_65) | 2007 | Jiande | Zhejiang | Construction workers | 196 | — | — | 0 | 529 |
| Zhang P, 2011[^66^](#_ENREF_66) | — | Kuancheng, Changchun | Jilin | Construction workers | 400 | ELISA | ELISA | 0 | 400 |
| Zhang Y, 2012[^67^](#_ENREF_67) | 2010 | Ningbo | Zhejiang | Construction workers | 925 | ELISA-1 | ELISA-2 | 0 | 925 |
| Zhao R, 2005[^68^](#_ENREF_68) | — | Gejiu City | Yunnan | Miners | 232 | ELISA | WB | 1 | 182 |
| He B, 2012[^56^](#_ENREF_56) | 2011 | Pinxiang | Guangxi | Factory workers | 403 | ELISA | WB | 0 | 403 |
| Huang RR, 2008[^69^](#_ENREF_69) | — | Jinshan District | Shanghai | Factory workers | 310 | ELISA | — | 0 | 310 |
| Mou XL, 2012[^60^](#_ENREF_60) | 2010 | Wuxi | Jiangsu | Factory workers | 1703 | ELISA | WB | 0 | 399 |
| Pu Y, 2011[^70^](#_ENREF_70) | 2010-2011 | Jingjiang | Jiangsu | Factory workers | 469 | — | — | 0 | 469 |
| Wei W, 2012[^71^](#_ENREF_71) | 2012 | Shanghai | Shanghai | Factory workers | 404 | — | — | 0 | 404 |
| Xia DY, 2011[^64^](#_ENREF_64) | 2009 | Beijing | Beijing | Factory workers | 59 | ELISA | WB | 0 | 59 |
| Zhang QC, 2010[^72^](#_ENREF_72) | 2007-2008 | Daishan | Zhejiang | Factory workers | 830 | — | — | 0 | 830 |
| Zhang Y, 2012[^67^](#_ENREF_67) | 2010 | Ningbo | Zhejiang | Factory workers | 1029 | ELISA-1 | ELISA-2 | 3 | 1029 |
| Mou XL, 2012[^60^](#_ENREF_60) | 2010 | Wuxi | Jiangsu | Restaurant attendants | 1703 | ELISA | WB | 0 | 400 |
| Zhang Y, 2012[^67^](#_ENREF_67) | 2010 | Ningbo | Zhejiang | Restaurant attendants | 1025 | ELISA-1 | ELISA-2 | 3 | 1025 |
| Zhang Y, 2012[^67^](#_ENREF_67) | 2010 | Ningbo | Zhejiang | Unspecified job nature | 1023 | ELISA-1 | ELISA-2 | 0 | 1023 |
| Cai T, 2010[^73^](#_ENREF_73) | — | Dalian | Liaoning | Unspecified job nature | 60 | ELISA | — | 0 | 60 |
| Cai W, 2005[^74^](#_ENREF_74) | 2003-2004 | Heze | Shandong | Unspecified job nature | 2137 | ELISA | WB | 0 | 2137 |
| Chen P, 2011[^75^](#_ENREF_75) | 2010 | Anxiang | Hunan | Unspecified job nature | 260 | ELISA | WB | 0 | 260 |
| Chen SH, 2012[^76^](#_ENREF_76) | 2010 | Foshan | Guangdong | Unspecified job nature | 1611 | — | — | 2 | 1611 |
| Cui DZ, 2012[^77^](#_ENREF_77) | 2012 | Lanzhou | Gansu | Unspecified job nature | 400 | ELISA | — | 0 | 400 |
| Ding XB, 2007[^78^](#_ENREF_78) | 2006 | Yuzhong, | Chongqing | Unspecified job nature | 1215 | ELISA | WB | 0 | 1215 |
| Fang DW, 2000[^79^](#_ENREF_79) | 1995-1998 | Dongxing | Guangxi | Unspecified job nature | 952 | ELISA | WB | 2 | 952 |
| Gu MD, 2009[^80^](#_ENREF_80) | 2007 | Pingjiang | Hunan | Unspecified job nature | 576 | ELISA | WB | 3 | 576 |
| **Table S5a Characteristics description of studies included in meta-analysis of HIV prevalence (Cont’d)** | | | | | | |  |  |  |
| **First Author &** | **Study** | **Study Location** |  | **Subgroup** | **Sample** | **Presumptive** | **Confirmatory** | **No. of** | **No. of people** |
| **Published Year** | **Period** | **City/county** | **Province** | **Population** | **Size** | **test(s)** | **test(s)*** | **infection** | **tested** |
| HIV(Cont’d) |  |  |  |  |  |  |  |  |  |
| Guo HJ, 2010a[^81^](#_ENREF_81) | 2008-2009 | Ningyang | Shandong | Unspecified job nature | 567 | — | YES | 0 | 567 |
| Guo HJ, 2010b[^81^](#_ENREF_81) | 2007-2008 | Ningyang | Shandong | Unspecified job nature | 226 | — | — | 0 | 226 |
| Guo HJ, 2010c[^81^](#_ENREF_81) | 2008-2009 | Ningyang | Shandong | Unspecified job nature | 108 | — | — | 2 | 108 |
| Guo HJ, 2010d[^81^](#_ENREF_81) | 2008-2009 | Ningyang | Shandong | Unspecified job nature | 502 | — | — | 1 | 502 |
| Guo WG, 2009[^82^](#_ENREF_82) | 2008 | Beihai | Guangxi | Unspecified job nature | 255 | YES | YES | 1 | 255 |
| He N, 2005[^83^](#_ENREF_83) | — | Shanghai | Shanghai | Unspecified job nature | 1086 | ELISA | WB | 0 | 986 |
| He QH, 2010[^84^](#_ENREF_84) | 2009 | Fushan | Guangdong | Unspecified job nature | 300 | ELISA | YES | 0 | 300 |
| Hesketh T, 2006[^85^](#_ENREF_85) | 2004 | Hangzhou | Zhejiang | Unspecified job nature | 4148 | PA | — | 0 | 4148 |
| Hong PK, 2010[^86^](#_ENREF_86) | 2010 | Jinjiang | Fujian | Unspecified job nature | 400 | — | — | 0 | 400 |
| Hu SY, 2003[^87^](#_ENREF_87) | — | Guiyang | Guizhou | Unspecified job nature | 1324 | — | — | 0 | 505 |
| Huang T, 2012[^88^](#_ENREF_88) | 2009 | Xichang | Sichuan | Unspecified job nature | 173 | ELISA | WB | 6 | 173 |
| Huang WJ, 2011[^89^](#_ENREF_89) | 2009 | Jiujiang | Jiangxi | Unspecified job nature | 600 | ELISA | WB | 0 | 600 |
| Huang XT, 2010[^90^](#_ENREF_90) | 2009 | Shantou | Guangdong | Unspecified job nature | 516 | YES | YES | 0 | 516 |
| Huang ZL, 2013[^91^](#_ENREF_91) | 2011 | Changsha | Hunan | Unspecified job nature | 1093 | ELISA | WB | 0 | 1093 |
| Jiang CH, 2012a[^92^](#_ENREF_92) | 2009 | Jinshan District | Shanghai | Unspecified job nature | 400 | ELISA | WB | 0 | 400 |
| Jiang CH, 2012b[^92^](#_ENREF_92) | 2010 | Jinshan District | Shanghai | Unspecified job nature | 405 | ELISA | WB | 0 | 405 |
| Jiang CH, 2012c[^92^](#_ENREF_92) | 2011 | Jinshan District | Shanghai | Unspecified job nature | 400 | ELISA | WB | 1 | 400 |
| Jiang JH, 2012[^93^](#_ENREF_93) | 2012 | Jiangyin | Jiangsu | Unspecified job nature | 400 | ELISA-1 | ELISA-2 | 0 | 400 |
| Jiang YF, 2010[^94^](#_ENREF_94) | 2009 | Hangzhou | Zhejiang | Unspecified job nature | 445 | ELISA-1 | ELISA-2 | 0 | 441 |
| Jing LH, 2010[^95^](#_ENREF_95) | 2009 | Yangquan | Shanxi | Unspecified job nature | 409 | ELISA | WB | 0 | 409 |
| Kang YF, 2011[^96^](#_ENREF_96) | 2008-2010 | Yangquan | Shanxi | Unspecified job nature | 813 | ELISA | WB | 0 | 813 |
| Lan LX, 2011[^97^](#_ENREF_97) | 2008-2010 | Nanning | Guangxi | Unspecified job nature | 1000 | Colloidal Gold | WB | 15 | 1000 |
| Li CY, 2011[^98^](#_ENREF_98) | 2011 | Daxin | Guangxi | Unspecified job nature | 4240 | — | — | 9 | 4240 |
| Li DM, 2007[^99^](#_ENREF_99) | 2006 | Houma | Shanxi | Unspecified job nature | 1190 | ELISA | — | 0 | 1125 |
| **Table S5a Characteristics description of studies included in meta-analysis of HIV prevalence (Cont’d)** | | | | | | |  |  |  |
| **First Author &** | **Study** | **Study Location** |  | **Subgroup** | **Sample** | **Presumptive** | **Confirmatory** | **No. of** | **No. of people** |
| **Published Year** | **Period** | **City/county** | **Province** | **Population** | **Size** | **test(s)** | **test(s)*** | **infection** | **tested** |
| HIV(Cont’d) |  |  |  |  |  |  |  |  |  |
| Li HL, 2011a[^100^](#_ENREF_100) | 2008 | Pu'er | Yunnan | Unspecified job nature | 1070 | ELISA | WB | 1 | 247 |
| Li HL, 2011b[^100^](#_ENREF_100) | 2009 | Pu'er | Yunnan | Unspecified job nature | 1070 | ELISA | WB | 2 | 416 |
| Li HL, 2011c[^100^](#_ENREF_100) | 2010 | Pu'er | Yunnan | Unspecified job nature | 1070 | ELISA | WB | 1 | 407 |
| Li R, 2009a[^101^](#_ENREF_101) | 2007 | Dalian | Liaoning | Unspecified job nature | 400 | ELISA | ELISA | 0 | 400 |
| Li R, 2009b[^101^](#_ENREF_101) | 2008 | Dalian | Liaoning | Unspecified job nature | 400 | ELISA | ELISA | 1 | 400 |
| Li XJ, 2011[^102^](#_ENREF_102) | 2009 | Hefei | Anhui | Unspecified job nature | 1657 | — | — | 0 | 1657 |
| Liao MZ, 2008[^42^](#_ENREF_42) | 2007 | Jinan | Shandong | Unspecified job nature | 818 | ELISA | ELISA | 0 | 818 |
| Liao MZ, 2010[^43^](#_ENREF_43) | 2009 | Jinan | Shandong | Unspecified job nature | 1128 | ELISA | ELISA | 3 | 1128 |
| Lin X, 2011[^103^](#_ENREF_103) | 2010 | Fujian | Fujian | Unspecified job nature | 1705 | ELISA-1 | ELISA-2 | 0 | 1705 |
| Lin YT, 2012[^104^](#_ENREF_104) | 2010 | Changle,Jinjiang,Meilie | Fujian | Unspecified job nature | 4167 | ELISA | ELISA | 1 | 4098 |
| Liu GH, 2012[^105^](#_ENREF_105) | 2010 | Xingan | Jiangxi | Unspecified job nature | 1793 | ELISA | WB | 6 | 1793 |
| Liu SZ, 2012[^106^](#_ENREF_106) | 2010 | Xuanhan | Sichuan | Unspecified job nature | 400 | ELISA | YES | 1 | 400 |
| Liu XZ, 2006[^44^](#_ENREF_44) | 2004 | Shandong | Shandong | Unspecified job nature | 832 | ELISA | ELISA | 0 | 832 |
| Lu JJ, 2011[^107^](#_ENREF_107) | 2011 | Jiangyin | Jiangsu | Unspecified job nature | 400 | ELISA-1 | ELISA-2 | 0 | 400 |
| Lu L, 2006[^108^](#_ENREF_108) | 1989-2005 | Yunnan | Yunnan | Unspecified job nature | 1624 | ELISA | WB | 9 | 1624 |
| Lv B, 2008a[^109^](#_ENREF_109) | 2006 | TongXiang | Zhejiang | Unspecified job nature | 899 | ELISA | WB | 0 | 899 |
| Lv B, 2008b[^109^](#_ENREF_109) | 2007 | TongXiang | Zhejiang | Unspecified job nature | 918 | ELISA | WB | 0 | 918 |
| Meng XJ, 2010[^110^](#_ENREF_110) | 2008 | Jilin,Baicheng,Tonghua,Baishan | Jilin | Unspecified job nature | 2113 | ELISA | WB | 1 | 1110 |
| Meng XJ, 2010[^111^](#_ENREF_111) | — | Hunan | Hunan | Unspecified job nature | 2233 | ELISA | WB | 7 | 2233 |
| Mou XL, 2012[^60^](#_ENREF_60) | 2010 | Wuxi | Jiangsu | Unspecified job nature | 1703 | ELISA | WB | 0 | 1688 |
| Mou XL, 2012[^60^](#_ENREF_60) | 2010 | Wuxi | Jiangsu | Unspecified job nature | 1703 | ELISA | WB | 0 | 407 |
| Mou XL, 2011[^112^](#_ENREF_112) | 2010 | Wuxi | Jiangsu | Unspecified job nature | 400 | ELISA-1 | ELISA-2 | 0 | 400 |
| **Table S5a Characteristics description of studies included in meta-analysis of HIV prevalence (Cont’d)** | | | | | | |  |  |  |
| **First Author &** | **Study** | **Study Location** |  | **Subgroup** | **Sample** | **Presumptive** | **Confirmatory** | **No. of** | **No. of people** |
| **Published Year** | **Period** | **City/county** | **Province** | **Population** | **Size** | **test(s)** | **test(s)*** | **infection** | **tested** |
| HIV (Cont’d) |  |  |  |  |  |  |  |  |  |
| Nan SZ, 2009[^11^](#_ENREF_11) | 2007 | Antu | Jilin | Unspecified job nature | 396 | ELISA | YES | 0 | 396 |
| Pan XH, 2002[^113^](#_ENREF_113) | 2001 | Hainan | Hainan | Unspecified job nature | 251 | ELISA | WB | 0 | 251 |
| Pan XH, 2013[^114^](#_ENREF_114) | 2010 | Nanjing | Zhejiang | Unspecified job nature | 17377 | ELISA | WB | 3 | 17377 |
| Qian ZY, 2011[^115^](#_ENREF_115) | 2010 | Xinghua | Jiangsu | Unspecified job nature | 511 | YES | WB | 1 | 511 |
| Qiu JQ, 2008[^116^](#_ENREF_116) | 2004-2006 | Kaihua | Zhejiang | Unspecified job nature | 1557 | — | — | 0 | 1557 |
| Qiu JQ, 2011[^117^](#_ENREF_117) | 2010 | Kaihua | Zhejiang | Unspecified job nature | 873 | ELISA | — | 0 | 873 |
| Ruan JJ, 2010[^118^](#_ENREF_118) | 2009 | Yiwu | Zhejiang | Unspecified job nature | 402 | ELISA | YES | 0 | 400 |
| Shi XJ, 2011[^119^](#_ENREF_119) | 2010 | Ningbo | Zhejiang | Unspecified job nature | 800 | ELISA-1 | ELISA-2 | 0 | 793 |
| Sun YL, 2011[^120^](#_ENREF_120) | 2003-2011 | Longkou | Shandong | Unspecified job nature | 48036 | ELISA | WB | 16 | 48036 |
| Tan Y, 2011[^121^](#_ENREF_121) | 2010 | Zhongshan | Guangdong | Unspecified job nature | 1600 | ELISA-1,ELISA-2 | WB | 2 | 1600 |
| Tang HL, 2009[^122^](#_ENREF_122) | — | Dongyang | Zhejiang | Unspecified job nature | 620 | — | — | 0 | 620 |
| Tang T, 2007[^123^](#_ENREF_123) | — | Jining | Shandong | Unspecified job nature | 1954 | PA | WB | 6 | 1954 |
| Wan LJ, 2009[^124^](#_ENREF_124) | 2007 | Shangyu | Zhejiang | Unspecified job nature | 403 | — | — | 0 | 403 |
| Wang LJ, 2006[^125^](#_ENREF_125) | — | Chongwen | Beijing | Unspecified job nature | 380 | — | — | 0 | 380 |
| Wang RT, 2009[^126^](#_ENREF_126) | 2006-2008 | Yancheng | Jiangsu | Unspecified job nature | 3218 | — | — | 1 | 3218 |
| Wang YL, 2006[^127^](#_ENREF_127) | 2006 | Yuzhong, Jiulongpo | Chongqing | Unspecified job nature | 2047 | ELISA | WB | 3 | 2047 |
| Wu ZH, 2002[^48^](#_ENREF_48) | 1995-2001 | Huaihua | Hunan | Unspecified job nature | 235 | ELISA | WB | 3 | 235 |
| Xia DY, 2011[^64^](#_ENREF_64) | 2009 | Beijing | Beijing | Unspecified job nature | 4187 | ELISA | WB | 0 | 4187 |
| Xia DY, 2011[^64^](#_ENREF_64) | 2009 | Beijing | Beijing | Unspecified job nature | 967 | ELISA | WB | 0 | 967 |
| Xiao Y, 2007[^128^](#_ENREF_128) | 2006 | Jiujiang | Jiangxi | Unspecified job nature | 563 | ELISA | WB | 0 | 563 |
| Xing AH, 2007[^129^](#_ENREF_129) | 2006 | Shaanxi | Shaanxi | Unspecified job nature | 2753 | ELISA | WB | 2 | 2753 |
| Xu H, 2009[^130^](#_ENREF_130) | 2007-2008 | Yandu | Jiangsu | Unspecified job nature | 2819 | ELISA | WB | 2 | 2819 |
| Xu Y, 2010[^131^](#_ENREF_131) | 2009 | Changxing | Shanghai | Unspecified job nature | 400 | — | — | 0 | 400 |
| **Table S5a Characteristics description of studies included in meta-analysis of HIV prevalence (Cont’d)** | | | | | | |  |  |  |
| **First Author &** | **Study** | **Study Location** |  | **Subgroup** | **Sample** | **Presumptive** | **Confirmatory** | **No. of** | **No. of people** |
| **Published Year** | **Period** | **City/county** | **Province** | **Population** | **Size** | **test(s)** | **test(s)*** | **infection** | **tested** |
| HIV(Cont’d) |  |  |  |  |  |  |  |  |  |
| Xue LJ, 2011[^132^](#_ENREF_132) | 2010 | Kunshan | Jiangsu | Unspecified job nature | 400 | — | — | 0 | 400 |
| Yan ZZ, 2007[^133^](#_ENREF_133) | 2006 | Chongyang | Hubei | Unspecified job nature | 400 | ELISA |  | 0 | 400 |
| Yang L, 2011[^134^](#_ENREF_134) | 2009-2011 | Dehong | Yunnan | Unspecified job nature | 2260 | yes | WB | 20 | 2260 |
| Yang MH, 2011[^135^](#_ENREF_135) | 2010 | Sanming | Fujian | Unspecified job nature | 402 | ELISA-1 | ELISA-2 | 0 | 402 |
| Yang X, 2007[^136^](#_ENREF_136) | 2003 | — | — | Unspecified job nature | 5499 | — | — | 11 | 5499 |
| Yao QW, 2010[^137^](#_ENREF_137) | 2009-2010 | Kunshan | Jiangsu | Unspecified job nature | 602 | ELISA-1 | ELISA-2 | 0 | 602 |
| Ye W, 2003[^138^](#_ENREF_138) | 2002 | Shanghai | Shanghai | Unspecified job nature | 1128 | ELISA | WB | 0 | 1041 |
| Yi ZM, 2008[^139^](#_ENREF_139) | 2006-2007 | Hunan | Hunan | Unspecified job nature | 6481 | ESISA-1 | ESISA2 | 12 | 6481 |
| Yin XW, 2010a[^140^](#_ENREF_140) | 2007-2008 | Ningyang | Shandong | Unspecified job nature | 226 | — | — | 0 | 226 |
| Yin XW, 2010b[^140^](#_ENREF_140) | 2008-2009 | Ningyang | Shandong | Unspecified job nature | 502 | — | — | 1 | 502 |
| Yin XW, 2010c[^140^](#_ENREF_140) | 2008-2009 | Ningyang | Shandong | Unspecified job nature | 567 | — | — | 0 | 567 |
| Yin XW, 2010d[^140^](#_ENREF_140) | 2008-2009 | Ningyang | Shandong | Unspecified job nature | 108 | — | — | 2 | 108 |
| Yu XF, 2011a[^141^](#_ENREF_141) | 2006 | Chongqing | Chongqing | Unspecified job nature | 604 | — | — | 0 | 604 |
| Yu XF, 2011b[^141^](#_ENREF_141) | 2007 | Chongqing | Chongqing | Unspecified job nature | 606 | — | — | 3 | 606 |
| Yu XF, 2011c[^141^](#_ENREF_141) | 2008 | Chongqing | Chongqing | Unspecified job nature | 718 | — | — | 1 | 718 |
| Yu XF, 2011d[^141^](#_ENREF_141) | 2009 | Chongqing | Chongqing | Unspecified job nature | 410 | — | — | 0 | 410 |
| Zang DA, 2008[^142^](#_ENREF_142) | 2006-2007 | Sheyang | Jiangsu | Unspecified job nature | 3554 | ELISA | YES | 1 | 3554 |
| Zhan SY, 2005[^143^](#_ENREF_143) | 2001-2003 | Yichun | Jiangxi | Unspecified job nature | 1565 | ELISA | WB | 5 | 341 |
| Zhang TL, 2012[^53^](#_ENREF_53) | — | Shanndong | Shandong | Unspecified job nature | 938 | ELISA-1 | ELISA-2 | 8 | 938 |
| Zhang WB, 2011[^32^](#_ENREF_32) | 2009 | Pingyao County | Shanxi | Unspecified job nature | 1000 | YES | YES | 5 | 1000 |
| Zhang Y, 2012[^67^](#_ENREF_67) | 2010 | Ningbo | Zhejiang | Unspecified job nature | 4002 | ELISA-1 | ELISA-2 | 6 | 4002 |
| Zhang Y, 2004[^54^](#_ENREF_54) | 2003-2003 | Hubei | Hubei | Unspecified job nature | 10520 | ELISA | WB | 0 | 10520 |
| Zhang YQ, 2008[^144^](#_ENREF_144) | 2007 | Zoucheng | Shandong | Unspecified job nature | 2006 | ELISA | YES | 1 | 1979 |
| Zhao YY, 2012[^145^](#_ENREF_145) | 2010-2011 | Zhuhai | Guangdong | Unspecified job nature | 823 | ELISA | — | 0 | 823 |
| **Table S5a Characteristics description of studies included in meta-analysis of HIV prevalence (Cont’d)** | | | | | | |  |  |  |
| **First Author &** | **Study** | **Study Location** |  | **Subgroup** | **Sample** | **Presumptive** | **Confirmatory** | **No. of** | **No. of people** |
| **Published Year** | **Period** | **City/county** | **Province** | **Population** | **Size** | **test(s)** | **test(s)*** | **infection** | **tested** |
| HIV(Cont’d) |  |  |  |  |  |  |  |  |  |
| Zhu JQ, 2012[^146^](#_ENREF_146) | 2010 | Jinzhou | Zhejiang | Unspecified job nature | 403 | ELISA | YES | 1 | 403 |
| Zhu YY, 2012[^147^](#_ENREF_147) | 2009 | Beijing | Beijing | Unspecified job nature | 4187 | ELISA | WB | 0 | 4187 |
| Chen JX, 2005[^148^](#_ENREF_148) | 2002-2003 | Shenzhen | Guangdong | Pregnant women | 4365 | ELISA | YES | 1 | 315 |
| Duan S, 2008[^149^](#_ENREF_149) | 2006 | Yunnan | Yunnan | Pregnant women | 1978 | ELISA | YES | 2 | 37 |
| Hong H, 2011[^150^](#_ENREF_150) | 2010 | Ningbo | Zhejiang | Pregnant women | 187 | — | — | 0 | 187 |
| Huang WH, 2012[^151^](#_ENREF_151) | 2010-2012 | Quanzhou | Fujian | Pregnant women | 2198 | ELISA | WB | 1 | 787 |
| Li SH, 2012a[^152^](#_ENREF_152) | 2007 | Beijing | Beijing | Pregnant women | 26819 | YES | — | 1 | 6730 |
| Li SH, 2012b[^152^](#_ENREF_152) | 2008 | Beijing | Beijing | Pregnant women | 26819 | YES | — | 0 | 6869 |
| Li SH, 2012c[^152^](#_ENREF_152) | 2009 | Beijing | Beijing | Pregnant women | 26819 | YES | — | 1 | 7076 |
| Li SH, 2012d[^152^](#_ENREF_152) | 2010 | Beijing | Beijing | Pregnant women | 26819 | YES | — | 1 | 6144 |
| Lu J, 2012[^153^](#_ENREF_153) | 2010 | Xinjiang | Xinjiang | Pregnant women | 4187 | ELISA-1/ELISA-2 | YES | 0 | 672 |
| Nong LP, 2011[^154^](#_ENREF_154) | 2009-2010 | Pingxiang | Guangxi | Pregnant women | 800 | ELISA | YES | 0 | 117 |
| Song JM, 2013[^155^](#_ENREF_155) | 2000-2010 | Shenzhen | Guangdong | Pregnant women | 1530577 | Colloidal Gold | WB | 210 | 1530577 |
| Yang L, 2012[^156^](#_ENREF_156) | 2005-2010 | Dehong | Yunnan | Pregnant women | 12742 | YES | WB | 8 | 760 |
| Bao FB, 2007[^157^](#_ENREF_157) | 2004 | Huhehaote | Inner Mongolia | FSWs | 624 | ELISA | YES | 0 | 519 |
| Cha YF, 2008a[^158^](#_ENREF_158) | 2006-2007 | Songjiang | Shanghai | FSWs | 529 | ELISA | — | 0 | 366 |
| Cha YF, 2008b[^158^](#_ENREF_158) | 2006-2007 | Songjiang | Shanghai | FSWs | 529 | ELISA | — | 0 | 405 |
| Chen FC, 2011[^159^](#_ENREF_159) | 2010 | Dangyang | Hubei | FSWs | 400 | ELISA | — | 0 | 49 |
| Chen GS, 2010[^160^](#_ENREF_160) | 2009 | — | Jiangxi | FSWs | 401 | ELISA | YES | 0 | 396 |
| Chen L, 2010[^161^](#_ENREF_161) | 2009 | Shenzhen | Guangdong | FSWs | 426 | YES | — | 0 | 404 |
| Chen SH, 2005[^162^](#_ENREF_162) | — | Foushan | Guangdong | FSWs | 271 | ELISA | — | 0 | 265 |
| Chen SP, 2010[^163^](#_ENREF_163) | 2007 | Jiujiang | Jiangxi | FSWs | 360 | ELISA | YES | 0 | 168 |
| Chen XS, 2012[^164^](#_ENREF_164) | 2009 | 6 cities | 3 Provinces | FSWs | 5322 | ELISA | WB | 12 | 3898 |
| Chen YL, 2005[^165^](#_ENREF_165) | 2004 | Longyan | Fujian | FSWs | 247 | YES | — | 0 | 135 |
| **Table S5a Characteristics description of studies included in meta-analysis of HIV prevalence (Cont’d)** | | | | | | |  |  |  |
| **First Author &** | **Study** | **Study Location** |  | **Subgroup** | **Sample** | **Presumptive** | **Confirmatory** | **No. of** | **No. of people** |
| **Published Year** | **Period** | **City/county** | **Province** | **Population** | **Size** | **test(s)** | **test(s)*** | **infection** | **tested** |
| HIV(Cont’d) |  |  |  |  |  |  |  |  |  |
| Chen ZB, 2011[^166^](#_ENREF_166) | 2007-2007 | Lianzhou | Guangdong | FSWs | 200 | ELISA-1/ELISA-2 | WB | 0 | 173 |
| Ci RWM, 2012[^167^](#_ENREF_167) | 2010 | Lasa | Tibet | FSWs | 442 | ELISA | WB | 0 | 393 |
| Dong XY, 2009[^168^](#_ENREF_168) | 2007 | Tianjin | Tianjin | FSWs | 178 | ELISA | YES | 0 | 170 |
| Dong HW, 2003[^169^](#_ENREF_169) | 2000 | Zhoushan | Zhejiang | FSWs | 113 | ELISA | — | 1 | 98 |
| Du YP, 2006[^170^](#_ENREF_170) | 2004 | Jinjiang | Jiangsu | FSWs | 369 | — | — | 0 | 240 |
| Du YP, 2003[^171^](#_ENREF_171) | 2001 | — | Jiangsu | FSWs | 401 | ELISA | YES | 0 | 260 |
| Duo JWM, 2008[^172^](#_ENREF_172) | 2006 | Lasa | Tibet | FSWs | 508 | ELISA | WB | 0 | 406 |
| Fang Q, 2005[^173^](#_ENREF_173) | — | Shenzhen | Guangdong | FSWs | 55 | — | — | 0 | 55 |
| Gan HW, 2012[^174^](#_ENREF_174) | 2009-2011 | Jinshan | Shanghai | FSWs | 1240 | ELISA | YES | 0 | 1230 |
| Gao CY, 2008[^175^](#_ENREF_175) | 2007 | Hegang | Heilongjiang | FSWs | 203 | ELISA | ELISA | 0 | 106 |
| Guo H, 2012[^176^](#_ENREF_176) | 2010-2011 | Longnan | Gansu | FSWs | 820 | ELISA | — | 0 | 104 |
| Guo HJ, 2012[^177^](#_ENREF_177) | 2010 | — | Guizhou | FSWs | 235 | ELISA | WB | 0 | 52 |
| Guo YY, 2010[^178^](#_ENREF_178) | 2001-2007 | Hangzhou | Zhejiang | FSWs | 1673 | ELISA | WB | 0 | 1582 |
| Hao XG, 2010[^179^](#_ENREF_179) | 2009 | Kecheng | Zhejiang | FSWs | 254 | — | — | 0 | 108 |
| Hao XG, 2012[^180^](#_ENREF_180) | 2011 | Quzhou | Zhejiang | FSWs | 400 | YES | YES | 0 | 176 |
| Hu JL, 2007[^181^](#_ENREF_181) | 2006 | Huaian | Jiangsu | FSWs | 256 | ELISA | WB | 0 | 106 |
| Huang JY, 2009[^182^](#_ENREF_182) | 2005-2008 | Qingyuan | Guangdong | FSWs | 227 | ELISA | WB | 0 | 168 |
| Huang T, 2006[^183^](#_ENREF_183) | 2003 | Shandong | Shandong | FSWs | 839 | ELISA | YES | 3 | 438 |
| Ji CH, 2009[^184^](#_ENREF_184) | 2004-2006 | Shanxi | Shanxi | FSWs | 904 | ELISA | — | 0 | 644 |
| Jiang HY, 2006[^185^](#_ENREF_185) | — | Shanxi | Shanxi | FSWs | 402 | ELISA | — | 0 | 242 |
| Jin TL, 2005[^186^](#_ENREF_186) | 2004 | Lishui | Zhejiang | FSWs | 140 | YES | — | 0 | 118 |
| Jin X, 2011[^187^](#_ENREF_187) | 2008 | Beijing | Beijing | FSWs | 568 | ELISA | YES | 78 | 395 |
| Li BY, 2012[^188^](#_ENREF_188) | 2010 | Jinnan | Tianjin | FSWs | 407 | ELISA | — | 0 | 382 |
| Li F, 2012[^189^](#_ENREF_189) | 2011 | Changping | Beijing | FSWs | 290 | ELISA | YES | 0 | 285 |
| **Table S5a Characteristics description of studies included in meta-analysis of HIV prevalence (Cont’d)** | | | | | | |  |  |  |
| **First Author &** | **Study** | **Study Location** |  | **Subgroup** | **Sample** | **Presumptive** | **Confirmatory** | **No. of** | **No. of people** |
| **Published Year** | **Period** | **City/county** | **Province** | **Population** | **Size** | **test(s)** | **test(s)*** | **infection** | **tested** |
| HIV(Cont’d) |  |  |  |  |  |  |  |  |  |
| Li GY, 2008[^190^](#_ENREF_190) | — | Beijing | Beijing | FSWs | 177 | YES | — | 0 | 173 |
| Li L, 2003[^191^](#_ENREF_191) | 2001 | Jiangsu | Jiangsu | FSWs | 385 | — | — | 0 | 187 |
| Li XF, 2006[^192^](#_ENREF_192) | 2003 | Qingdao | Shandong | FSWs | 466 | ELISA | YES | 0 | 233 |
| Li XJ, 2005[^193^](#_ENREF_193) | 2004 | Hefei | Anhui | FSWs | 364 | ELISA | YES | 0 | 104 |
| Li Y, 2009[^194^](#_ENREF_194) | 2006-2007 | — | Guangdong | FSWs | 320 | ELISA | YES | 0 | 297 |
| Liang HX, 2011[^195^](#_ENREF_195) | 2009-2010 | Shaya | Hubei | FSWs | 800 | ELISA | YES | 0 | 714 |
| Liao CQ, 2011[^196^](#_ENREF_196) | — | Hongkou | Shanghai | FSWs | 401 | — | — | 0 | 401 |
| Lin Z, 2007[^197^](#_ENREF_197) | 2005 | Tongliao | Inner Mongolia | FSWs | 364 | ELISA | YES | 0 | 58 |
| Liu HX, 2011[^198^](#_ENREF_198) | 2005-2010 | Beijing | Beijing | FSWs | 547 | — | — | 0 | 534 |
| Liu LL, 2011[^199^](#_ENREF_199) | 2010 | Jiangyan | Jiangsu | FSWs | 407 | ELISA | — | 0 | 155 |
| Liu LR, 2007[^200^](#_ENREF_200) | 2006 | Beijing | Beijing | FSWs | 341 | ELISA | YES | 0 | 328 |
| Liu S, 2008[^201^](#_ENREF_201) | 2006 | Xintai | Shandong | FSWs | 307 | ELISA | YES | 0 | 232 |
| Liu SF, 2007[^202^](#_ENREF_202) | — | Changzhou | Jiangsu | FSWs | 257 | ELISA | YES | 0 | 189 |
| Liu SH, 2009[^203^](#_ENREF_203) | 2008 | Shizuishan | Ningxia | FSWs | 366 | ELISA-1 | ELISA-2 | 0 | 339 |
| Liu XF, 2003[^204^](#_ENREF_204) | 2001 | — | Gansu | FSWs | 203 | ELISA | TRUST | 0 | 106 |
| Liu XZ, 2006[^44^](#_ENREF_44) | 2004 | Shandong | Shandong | FSWs | 1251 | ELISA-1 | ELISA-2 | 1 | 1251 |
| Liu ZJ, 2012[^205^](#_ENREF_205) | 2011 | Luannan | Hubei | FSWs | 400 | ELISA | — | 0 | 132 |
| Lu PN, 2002[^206^](#_ENREF_206) | 2001 | Panzhihua | Sichuan | FSWs | 306 | YES | TES | 0 | 235 |
| Luo XR, 2008[^207^](#_ENREF_207) | 2006 | Yibin | Sichuan | FSWs | 216 | ELISA | ELISA | 0 | 93 |
| Luo Y, 2008[^208^](#_ENREF_208) | 2006 | Hangzhou | Zhejiang | FSWs | 262 | ELISA | ELISA | 0 | 224 |
| Pan XL, 2008[^209^](#_ENREF_209) | 2005-2006 | Baise | Guangxi | FSWs | 253 | ELISA-1 | ELISA-2 | 0 | 176 |
| Pan XL, 2009a[^210^](#_ENREF_210) | 2006 | Baise | Guangxi | FSWs | 243 | ELISA-1 | ELISA-2 | 0 | 77 |
| Pan XL, 2009b[^210^](#_ENREF_210) | 2007 | Baise | Guangxi | FSWs | 243 | ELISA-1 | ELISA-2 | 0 | 97 |
| Pan XL, 2009c[^210^](#_ENREF_210) | 2008 | Baise | Guangxi | FSWs | 243 | ELISA-1 | ELISA-2 | 1 | 46 |
| **Table S5a Characteristics description of studies included in meta-analysis of HIV prevalence (Cont’d)** | | | | | | |  |  |  |
| **First Author &** | **Study** | **Study Location** |  | **Subgroup** | **Sample** | **Presumptive** | **Confirmatory** | **No. of** | **No. of people** |
| **Published Year** | **Period** | **City/county** | **Province** | **Population** | **Size** | **test(s)** | **test(s)*** | **infection** | **tested** |
| HIV(Cont’d) |  |  |  |  |  |  |  |  |  |
| Pan XL, 2010[^211^](#_ENREF_211) | 2006-2008 | Baise | Guangxi | FSWs | 269 | ELISA-1 | ELISA-2 | 1 | 220 |
| Peng B, 2008[^212^](#_ENREF_212) | 2007 | Jiujiang | Jiangxi | FSWs | 250 | ELISA | YES | 0 | 170 |
| Qin CM, 2009[^213^](#_ENREF_213) | 2007 | Zhaoyuan | Shandong | FSWs | 197 | ELISA | YES | 0 | 135 |
| Qin CZ, 2012[^214^](#_ENREF_214) | 2010-2011 | Jiangyan | Jiangsu | FSWs | 817 | ELISA | WB | 1 | 347 |
| Shao MC, 2010[^215^](#_ENREF_215) | — | Suzhou | Jiangsu | FSWs | 402 | ELISA | YES | 0 | 316 |
| Shi WY, 2007[^216^](#_ENREF_216) | 2005 | Fengtai | Beijing | FSWs | 114 | ELISA | YES | 0 | 113 |
| Shu HY, 2009[^217^](#_ENREF_217) | — | Zoucheng | Shandong | FSWs | 1021 | ELISA | YES | 0 | 955 |
| Song SJ, 2008[^218^](#_ENREF_218) | — | Hangzhou | Zhejiang | FSWs | 272 | ELISA | WB | 0 | 228 |
| Song ZP, 2004[^219^](#_ENREF_219) | — | Taiyuan | Shanxi | FSWs | 130 | ELISA | YES | 0 | 126 |
| Tan JG, 2009[^220^](#_ENREF_220) | 2008 | Shenzhen | Guangdong | FSWs | 335 | ELISA | YES | 0 | 309 |
| Tang X, 2012a[^221^](#_ENREF_221) | 2008 | Shanghai | Shanghai | FSWs | 244 | ELISA | WB | 0 | 244 |
| Tang X, 2012b[^221^](#_ENREF_221) | 2008 | Shanghai | Shanghai | FSWs | 360 | ELISA | WB | 0 | 360 |
| Tang ZL, 2011[^222^](#_ENREF_222) | 2010 | Qingdao | Shandong | FSWs | 322 | ELISA | YES | 0 | 174 |
| Wang GH, 2011[^223^](#_ENREF_223) | — | Tongxiang | Zhejiang | FSWs | 354 | — | — | 0 | 304 |
| Wang JW, 2005[^224^](#_ENREF_224) | — | Jiaxing | Zhejiang | FSWs | 169 | ELISA | — | 0 | 76 |
| Wang JY, 2010[^225^](#_ENREF_225) | 2009 | Zhongshan | Guangdong | FSWs | 520 | ELISA | YES | 1 | 475 |
| Wang L, 2008[^226^](#_ENREF_226) | 2007 | Yixing | Jiangsu | FSWs | 270 | ELISA | YES | 0 | 194 |
| Wang LW, 2006[^227^](#_ENREF_227) | 2004-2005 | Nanchang | Jiangxi | FSWs | 402 | ELISA | YES | 0 | 237 |
| Wang WM, 2008[^228^](#_ENREF_228) | 2007 | Kunshan | Yunnan | FSWs | 297 | ELISA | YES | 0 | 277 |
| Wang XM, 2011[^229^](#_ENREF_229) | 2007-2010 | — | Zhejiang | FSWs | 1078 | ELISA | YES | 0 | 427 |
| Wang XX, 2005[^230^](#_ENREF_230) | 2003 | Dongguan | Guangdong | FSWs | 103 | ELISA | WB | 0 | 97 |
| Wang Y, 2007[^231^](#_ENREF_231) | 2005 | Tongling | Anhui | FSWs | 431 | ELISA | YES | 0 | 120 |
| Wei XW, 2004a[^232^](#_ENREF_232) | 2003 | Shihezi | Xinjiang | FSWs | 181 | ELISA-1/ELISA-2 | WB | 0 | 68 |
| Wei XW, 2004b[^232^](#_ENREF_232) | 2003 | Shihezi | Xinjiang | FSWs | 459 | ELISA-1/ELISA-2 | WB | 0 | 255 |
| **Table S5a Characteristics description of studies included in meta-analysis of HIV prevalence (Cont’d)** | | | | | | |  |  |  |
| **First Author &** | **Study** | **Study Location** |  | **Subgroup** | **Sample** | **Presumptive** | **Confirmatory** | **No. of** | **No. of people** |
| **Published Year** | **Period** | **City/county** | **Province** | **Population** | **Size** | **test(s)** | **test(s)*** | **infection** | **tested** |
| HIV(Cont’d) |  |  |  |  |  |  |  |  |  |
| Wei ZY, 2010[^233^](#_ENREF_233) | 2009 | Yangzhou | Jiangsu | FSWs | 940 | ELISA | YES | 0 | 613 |
| Wen XQ, 2009[^234^](#_ENREF_234) | 2007 | Guilin | Guangxi | FSWs | 360 | ELISA | YES | 0 | 68 |
| Wu J, 2006[^235^](#_ENREF_235) | 2006 | Yingtan | Jiangxi | FSWs | 284 | — | — | 0 | 63 |
| Wu J, 2010a[^236^](#_ENREF_236) | 2008 | Zhangjiagang | Jiangsu | FSWs | 257 | ELISA | — | 0 | 246 |
| Wu J, 2010b[^236^](#_ENREF_236) | 2008 | Zhangjiagang | Jiangsu | FSWs | 217 | ELISA | — | 0 | 213 |
| Wu J, 2012[^237^](#_ENREF_237) | 2011 | Wuhu | Anhui | FSWs | 405 | — | — | 0 | 168 |
| Wu MS, 2006[^238^](#_ENREF_238) | 2005 | Guangxi | Guangxi | FSWs | 187 | — | — | 0 | 140 |
| Xiang SB, 2010[^239^](#_ENREF_239) | 2009 | Hongjiang | Hunan | FSWs | 401 | — | — | 0 | 63 |
| Xiao Y, 2002[^240^](#_ENREF_240) | 2002 | Jiujiang | Jiangxi | FSWs | 201 | ELISA | — | 0 | 106 |
| Xu GZ, 2002[^241^](#_ENREF_241) | 1998-1999 | Ningbo | Zhejiang | FSWs | 179 | ELISA | WB | 0 | 155 |
| Xu HF, 2011[^242^](#_ENREF_242) | 2009 | Shangqiu | Henan | FSWs | 200 | ELISA | WB | 0 | 68 |
| Xu HQ, 2008[^243^](#_ENREF_243) | 2006 | Jiaxing | Zhejiang | FSWs | 151 | — | — | 0 | 142 |
| Xu XY, 2011[^244^](#_ENREF_244) | 2009 | Huhehaote | Inner Mongolia | FSWs | 400 | ELISA | WB | 1 | 153 |
| Yan LM, 2007[^245^](#_ENREF_245) | — | Xiaogan | Hubei | FSWs | 182 | ELISA | — | 0 | 132 |
| Yan WZ, 2010[^246^](#_ENREF_246) | 2009 | Jionghong | Yunnan | FSWs | 188 | ELISA | YES | 0 | 51 |
| Yang SP, 2008[^247^](#_ENREF_247) | 2007 | Hami | Xinjiang | FSWs | 251 | ELISA | WB | 0 | 113 |
| Yang Y, 2011[^248^](#_ENREF_248) | 2008-2009 | Minhang | Shanghai | FSWs | 793 | ELISA | WB | 0 | 793 |
| Yao Y, 2012[^249^](#_ENREF_249) | 2007 | Yunnan | Yunnan | FSWs | 400 | ELISA | WB | 24 | 287 |
| Ye YD, 2011[^250^](#_ENREF_250) | 2010 | Changshu | Jiangsu | FSWs | 1619 | ELISA | YES | 0 | 1450 |
| Ye ZM, 2012[^251^](#_ENREF_251) | 2011 | Wenzhou | Zhejiang | FSWs | 830 | ELISA | YES | 0 | 713 |
| Yu X, 2007[^252^](#_ENREF_252) | 2006 | Haimen | Jiangsu | FSWs | 121 | ELISA | — | 0 | 75 |
| Zeng Y, 2009[^253^](#_ENREF_253) | — | Qingtian | Zhejiang | FSWs | 217 | ELISA | WB | 0 | 144 |
| Zhang GS, 2009[^254^](#_ENREF_254) | 2005 | Shantou | Guangdong | FSWs | 512 | ELISA | WB | 0 | 331 |
| Zhang H, 2011[^255^](#_ENREF_255) | 2006 | Changle | Fujian | FSWs | 267 | ELISA-1 | ELISA-2 | 0 | 267 |
| **Table S5a Characteristics description of studies included in meta-analysis of HIV prevalence (Cont’d)** | | | | | | |  |  |  |
| **First Author &** | **Study** | **Study Location** |  | **Subgroup** | **Sample** | **Presumptive** | **Confirmatory** | **No. of** | **No. of people** |
| **Published Year** | **Period** | **City/county** | **Province** | **Population** | **Size** | **test(s)** | **test(s)*** | **infection** | **tested** |
| HIV (Cont’d) |  |  |  |  |  |  |  |  |  |
| Zhang MN, 2011[^256^](#_ENREF_256) | 2010 | Shanxi | Shanxi | FSWs | 419 | — | — | 0 | 207 |
| Zhang Q, 2008[^257^](#_ENREF_257) | — | Wuhu | Anhui | FSWs | 400 | ELISA | — | 0 | 163 |
| Zhang YX, 2011[^258^](#_ENREF_258) | 2007 | Liuzhou | Guangxi | FSWs | 403 | ELISA-1 | ELISA-2 | 0 | 90 |
| Zhao AL, 2011[^259^](#_ENREF_259) | 2009 | Lanzhou | Gansu | FSWs | 350 | ELISA | YES | 0 | 174 |
| Zhao GD, 2011[^260^](#_ENREF_260) | 2010 | Shanluo | Shaanxi | FSWs | 337 | ELISA | YES | 0 | 75 |
| Zhao SH, 2007[^261^](#_ENREF_261) | 2006-2007 | Xiangtan | Hunan | FSWs | 140 | ELISA | WB | 5 | 81 |
| Zheng J, 2012[^262^](#_ENREF_262) | 2009-2010 | Anshan | Liaoning | FSWs | 400 | ELISA | WB | 0 | 31 |
| Zheng WA, 2009[^263^](#_ENREF_263) | 2002-2008 | Hainan | Hainan | FSWs | 1144 | ELISA | — | 0 | 903 |
| Zhou CX, 2011[^264^](#_ENREF_264) | 2010 | Zunyi | Guizhou | FSWs | 235 | — | — | 0 | 54 |
| Zhou XL, 2007[^265^](#_ENREF_265) | 2007 | Shenzhen | Guangdong | FSWs | 271 | — | — | 0 | 265 |
| Zhu FG, 2008[^266^](#_ENREF_266) | 2006 | Yancheng | Jiangsu | FSWs | 252 | — | — | 0 | 190 |
| Zhu HW, 2012[^267^](#_ENREF_267) | 2011 | Nanchang | Jiangxi | FSWs | 400 | — | — | 0 | 90 |
| Zhu TW, 2006[^268^](#_ENREF_268) | 2004 | Linqi | Shandong | FSWs | 323 | — | — | 0 | 271 |
| Zou Y, 2005[^269^](#_ENREF_269) | 2004 | — | Zhejiang | FSWs | 452 | ELISA | YES | 0 | 376 |
| He Q, 2006[^270^](#_ENREF_270) | 2003 | Guangzhou | Guangdong | MSM | 201 | ELISA | WB | 0 | 125 |
| Cai WD, 2010[^271^](#_ENREF_271) | — | Shenzhen | Guangdong | MSM | 394 | ELISA | WB | 20 | 382 |
| Chen XX, 2011[^272^](#_ENREF_272) | 2010 | Beijing | Beijing | MSM | 283 | ELISA-1 | ELISA-2 | 3 | 63 |
| Ding XB, 2010[^273^](#_ENREF_273) | 2008 | Chongqing | Chongqing | MSM | 743 | ELISA | WB | 30 | 163 |
| He Q, 2009[^274^](#_ENREF_274) | 2006 | Guangzhou | Guangdong | MSM | 380 | ELISA | WB | 5 | 350 |
| Choi KH, 2003[^275^](#_ENREF_275) | 2001-2002 | Beijing | Beijing | MSM | 481 | ELISA | WB | 9 | 312 |
| Lan GH, 2009[^276^](#_ENREF_276) | 2008 | Guangxi | Guangxi | MSM | 179 | ELISA-1,ELISA-2 | WB | 3 | 179 |
| Liang L, 2009[^277^](#_ENREF_277) | 2008 | Shijiazhuang | Hebei | MSM | 450 | ELISA | WB | 4 | 200 |
| Liu PL, 2010[^278^](#_ENREF_278) | 2008 | Wuhan | Hubei | MSM | 456 | ELISA | WB | 19 | 237 |
| Liu ZQ, 2009[^279^](#_ENREF_279) | 2006-2007 | Tianjin | Tianjin | MSM | 89 | ELISA | WB | 5 | 83 |
| **Table S5a Characteristics description of studies included in meta-analysis of HIV prevalence (Cont’d)** | | | | | | |  |  |  |
| **First Author &** | **Study** | **Study Location** |  | **Subgroup** | **Sample** | **Presumptive** | **Confirmatory** | **No. of** | **No. of people** |
| **Published Year** | **Period** | **City/county** | **Province** | **Population** | **Size** | **test(s)** | **test(s)*** | **infection** | **tested** |
| HIV (Cont’d) |  |  |  |  |  |  |  |  |  |
| Mei L, 2009[^280^](#_ENREF_280) | 2008 | Taiyuan | Shanxi | MSM | 273 | YES | WB | 9 | 184 |
| Qiao XW, 2009[^281^](#_ENREF_281) | 2008 | Lanzhou | Gansu | MSM | 619 | ELISA | WB | 4 | 85 |
| Qu L, 2011[^282^](#_ENREF_282) | 2010 | Inner Mongolia | Inner Mongolia | MSM | 805 | YES | WB | 4 | 64 |
| Ruan YH, 2007[^283^](#_ENREF_283) | 2005 | Beijing | Beijing | MSM | 526 | ELISA | WB | 2 | 188 |
| Wang B, 2013[^284^](#_ENREF_284) | 2008-2009 | Beijing | Beijing | MSM | 307 | ELISA | WB | 18 | 307 |
| Wang C, 2008[^285^](#_ENREF_285) | 2006-2007 | Beijing | Beijing | MSM | 541 | ELISA | WB | 20 | 356 |
| Wang Y, 2009[^286^](#_ENREF_286) | 2007 | Mianyang | Sichuan | MSM | 111 | ELISA | WB | 5 | 43 |
| Wang ZJ, 2010[^287^](#_ENREF_287) | 2008-2009 | Yangzhou | Jiangsu | MSM | 750 | Colloidal Gold | WB | 46 | 473 |
| Wen F, 2010[^288^](#_ENREF_288) | 2008 | Guangzhou | Guangdong | MSM | 452 | ELISA-1/ELISA-2 | WB | 19 | 290 |
| Xiao Y, 2009[^289^](#_ENREF_289) | 2007 | Chongqing | Chongqing | MSM | 1692 | ELISA | WB | 48 | 373 |
| Xiao Y, 2010[^290^](#_ENREF_290) | 2007 | 7 sites | — | MSM | 4657 | ELISA | WB | 41 | 1301 |
| Xu J, 2010[^291^](#_ENREF_291) | 2008 | 4 cities | 4 provinces | MSM | 1950 | Colloidal Gold | WB | 78 | 1084 |
| Xu JJ, 2011[^292^](#_ENREF_292) | 2008-2009 | Liaoning | Liaoning | MSM | 436 | ELISA | WB | 0 | 41 |
| Yang GZ, 2011[^293^](#_ENREF_293) | 2010 | Inner Mongolia | Inner Mongolia | MSM | 305 | ELISA | WB | 1 | 40 |
| Zhang QJ, 2010[^294^](#_ENREF_294) | 2009 | Tangshan | Hubei | MSM | 106 | ELISA | WB | 1 | 33 |
| Zheng M, 2011[^295^](#_ENREF_295) | 2009 | Guiyang | Guizhou | MSM | 357 | ELISA | WB | 32 | 148 |
| Zhou C, 2011[^296^](#_ENREF_296) | — | Chongqing | Chongqing | MSM | 1166 | YES | YES | 42 | 224 |
| Zhou ZH, 2010[^297^](#_ENREF_297) | 2008 | Beijing | Beijing | MSM | 550 | ELISA | WB | 23 | 392 |
| Zou HC, 2010[^298^](#_ENREF_298) | 2007 | Beijing | Beijing | MSM | 429 | ELISA | WB | 17 | 259 |
| Ao X, 2007[^299^](#_ENREF_299) | 2005 | Beijing | Beijing | DUs | 904 | ELISA | — | 9 | 250 |
| Zeng YM, 2005[^300^](#_ENREF_300) | 2000-2003 | Quzhou | Fujian | DUs | 1252 | ELISA | YES | 10 | 633 |
| Chen LP, 2008[^301^](#_ENREF_301) | 2007 | Qingyuan | Guangdong | DUs | 1073 | ELISA | WB | 1 | 73 |
| Chen YQ, 2006[^302^](#_ENREF_302) | 2004 | Shaoxin | Zhejiang | DUs | 185 | ELISA-1/ELISA-2 | YES | 13 | 146 |
| Deng SL, 2008a[^303^](#_ENREF_303) | 2007 | Huizhou | Guangdong | DUs | 592 | — | — | 9 | 181 |
| **Table S5a Characteristics description of studies included in meta-analysis of HIV prevalence (Cont’d)** | | | | | | |  |  |  |
| **First Author &** | **Study** | **Study Location** |  | **Subgroup** | **Sample** | **Presumptive** | **Confirmatory** | **No. of** | **No. of people** |
| **Published Year** | **Period** | **City/county** | **Province** | **Population** | **Size** | **test(s)** | **test(s)*** | **infection** | **tested** |
| HIV (Cont’d) |  |  |  |  |  |  |  |  |  |
| Deng SL, 2008b[^303^](#_ENREF_303) | 2008 | Huizhou | Guangdong | DUs | 486 | — | — | 8 | 486 |
| Dong HW, 2004[^304^](#_ENREF_304) | 1998-2003 | Zhoushan | Zhejiang | DUs | 71 | YES | YES | 8 | 71 |
| Feng ZJ, 2002[^305^](#_ENREF_305) | 2000-2001 | Dongguan | Guangdong | DUs | 953 | ELISA | WB | 22 | 608 |
| Fu DL, 2009[^306^](#_ENREF_306) | 2009 | Nanchan | Hubei | DUs | 400 | ELISA | YES | 207 | 400 |
| Huang F, 2005[^307^](#_ENREF_307) | — | Shenzhen | Guangdong | DUs | 86 | — | — | 3 | 86 |
| Huang JY, 2004[^308^](#_ENREF_308) | 2001-2003 | Shantou | Guangdong | DUs | 1160 | ELISA | — | 6 | 211 |
| Huang JY, 2007[^309^](#_ENREF_309) | 2000-2005 | Qingyuan | Guangdong | DUs | 2958 | ELISA | WB | 15 | 578 |
| Xie CH, 2009[^310^](#_ENREF_310) | 2005-2008 | Shandong | Shandong | DUs | 814 | YES | YES | 64 | 368 |
| Li DL, 2012[^311^](#_ENREF_311) | 2007 | Beijing | Beijing | DUs | 128 | ELISA | WB | 55 | 128 |
| Li GJ, 2002[^312^](#_ENREF_312) | 2001 | Zhongshan | Guangdong | DUs | 78 | ELISA | ELISA | 7 | 78 |
| Li K, 2006[^313^](#_ENREF_313) | 2005 | Guangzhou | Guangdong | DUs | 443 | ELISA | YES | 18 | 248 |
| Li XM, 2002[^314^](#_ENREF_314) | 1999 | — | Gansu | DUs | 871 | ELISA | YES | 8 | 116 |
| Liang ZJ, 2005[^315^](#_ENREF_315) | 2003-2004 | Guangzhou | Guangdong | DUs | 739 | ELISA | WB | 30 | 388 |
| Lin DZ, 2009a[^316^](#_ENREF_316) | 2008 | Huizhou | Guangdong | DUs | 592 | ELISA | WB | 9 | 389 |
| Lin DZ, 2009b[^316^](#_ENREF_316) | 2009 | Huizhou | Guangdong | DUs | 592 | ELISA | WB | 8 | 179 |
| Liu LC, 2007[^317^](#_ENREF_317) | 2001-2005 | Taizhou | Zhejiang | DUs | 3032 | ELISA | WB | 9 | 1237 |
| Liu LC, 2003[^318^](#_ENREF_318) | 2001-2002 | Taizhou | Zhejiang | DUs | 1188 | ELISA | WB | 7 | 306 |
| Liu SF, 2008[^319^](#_ENREF_319) | 2005-2007 | Changzhou | Jiangsu | DUs | 855 | ELISA | — | 18 | 328 |
| Liu XP, 2003[^320^](#_ENREF_320) | 2002 | Huhehaote | Inner Mongolia | DUs | 262 | ELISA | WB | 10 | 50 |
| Liu YH, 2007[^321^](#_ENREF_321) | 2005 | Beijing | Beijing | DUs | 486 | ELISA | — | 4 | 35 |
| Liu ZQ, 2005[^322^](#_ENREF_322) | 2004 | Dongguan | Guangdong | DUs | 1649 | ELISA | WB | 116 | 1649 |
| Luo WQ, 2012[^323^](#_ENREF_323) | 2010-2012 | Taishan | Guangdong | DUs | 1386 | ELISA | — | 4 | 206 |
| Nie XQ, 2005[^324^](#_ENREF_324) | 2004 | Xinhui | Guangdong | DUs | 512 | YES | YES | 15 | 150 |
| Qiu H, 2012[^325^](#_ENREF_325) | 2010 | Kunming | Yunnan | DUs | 872 | ELISA | WB | 16 | 162 |
| **Table S5a Characteristics description of studies included in meta-analysis of HIV prevalence (Cont’d)** | | | | | | |  |  |  |
| **First Author &** | **Study** | **Study Location** |  | **Subgroup** | **Sample** | **Presumptive** | **Confirmatory** | **No. of** | **No. of people** |
| **Published Year** | **Period** | **City/county** | **Province** | **Population** | **Size** | **test(s)** | **test(s)*** | **infection** | **tested** |
| HIV (Cont’d) |  |  |  |  |  |  |  |  |  |
| Sun DL, 2008[^326^](#_ENREF_326) | 2005-2006 | Lianyungang | Jiangsu | DUs | 913 | ELISA | YES | 0 | 695 |
| Tang JH, 2004[^327^](#_ENREF_327) | 2001-2002 | Shaoguan | Guangdong | DUs | 549 | ELISA | WB | 2 | 103 |
| Tao XY, 2004[^328^](#_ENREF_328) | 2003 | Shenzhen | Guangdong | DUs | 198 | YES | — | 3 | 49 |
| Wang FQ, 2002[^329^](#_ENREF_329) | 2000-2001 | Haikou | Hainan | DUs | 341 | YES | YES | 0 | 69 |
| Wang J, 2007[^330^](#_ENREF_330) | 2005-2006 | Suzhou | Jiangsu | DUs | 613 | ELISA | YES | 10 | 350 |
| Wang M, 2005[^331^](#_ENREF_331) | 2001-2004 | Taiyuan | Shanxi | DUs | 1269 | ELISA | WB | 3 | 86 |
| Wang SJ, 2007[^332^](#_ENREF_332) | 2005-006 | Zhejiang | Zhejiang | DUs | 125 | ELISA-1/ELISA-2 | WB | 5 | 99 |
| Wang Y, 2007[^333^](#_ENREF_333) | 2004-2006 | Jining | Shandong | DUs | 292 | YES | — | 42 | 183 |
| Wei XL, 2008[^334^](#_ENREF_334) | 2005-2007 | Xi'an | Shaanxi | DUs | 2267 | — | — | 32 | 177 |
| Wu QH, 2010a[^335^](#_ENREF_335) | 2007 | Taizhou | Zhejiang | DUs | 300 | ELISA | WB | 1 | 296 |
| Wu QH, 2010b[^335^](#_ENREF_335) | 2008 | Taizhou | Zhejiang | DUs | 363 | ELISA | WB | 2 | 363 |
| Wu QH, 2010c[^335^](#_ENREF_335) | 2009 | Taizhou | Zhejiang | DUs | 444 | ELISA | WB | 5 | 444 |
| Xie HY, 2005[^336^](#_ENREF_336) | 2000-2003 | Quanzhou | Fujian | DUs | 933 | ELISA | — | 7 | 440 |
| Xiong JG, 2007[^337^](#_ENREF_337) | 2002-2004 | Dongguan | Guangdong | DUs | 3766 | ELISA | YES | 193 | 2998 |
| Yang JZ, 2001[^338^](#_ENREF_338) | 1999 | Leqing | Zhejiang | DUs | 1712 | ELISA | WB | 9 | 516 |
| Yang ZM, 2004[^339^](#_ENREF_339) | 2001-2002 | Jiujiang | Jiangxi | DUs | 445 | ELISA | WB | 0 | 235 |
| Zhang H, 2010[^340^](#_ENREF_340) | 2006-2007 | Zhenning | Guizhou | DUs | 476 | ELISA | WB | 63 | 476 |
| Zhang L, 2012[^341^](#_ENREF_341) | 2009-2010 | Guangzhou | Guangdong | DUs | 4827 | ELISA-1 | YES | 47 | 1337 |
| Zhang ZY, 2008[^342^](#_ENREF_342) | 2007 | Lianyungang | Jiangsu | DUs | 488 | ELISA | YES | 2 | 35 |
| Zhao Q, 2008[^343^](#_ENREF_343) | 2004-2006 | Tengchong | Yunnan | DUs | 1618 | ELISA | YES | 6 | 156 |
| Zhong FH, 2008[^344^](#_ENREF_344) | 2005 | Yulin | Guangxi | DUs | 582 | — | — | 1 | 30 |
| Zhou HJ, 2001[^345^](#_ENREF_345) | 1998-2001 | Wuzhou | Guangxi | DUs | 1178 | ELISA-1 | ELISA-2 | 14 | 174 |
| Zhuo F, 2005[^346^](#_ENREF_346) | 2004 | Foshan | Guangdong | DUs | 344 | ELISA | YES | 7 | 203 |
|  |  |  |  |  |  |  |  |  |  |
| **Table S5b Characteristics description of studies included in meta-analysis of syphilis prevalence** | | | | | | |  |  |  |
| **First Author &** | **Study** | **Study Location** |  | **Subgroup** | **Sample** | **Presumptive** | **Confirmatory** | **No. of** | **No. of people** |
| **Published Year** | **Period** | **City/county** | **Province** | **Population** | **Size** | **test(s)** | **test(s)*** | **infection** | **tested** |
| Syphilis |  |  |  |  |  |  |  |  |  |
| Cheng XL, 2004[^347^](#_ENREF_347) | 1998-2002 | Wuyi | Zhejiang | Migrant women | 9455 | RPR | TPHA | 46 | 9455 |
| Ding ZW, 2010[^2^](#_ENREF_2) | 2008 | Jilin | Jilin | Migrant women | 1530 | RPR | TPPA | 0 | 838 |
| Liu KJ, 2009[^7^](#_ENREF_7) | 2006-2008 | Zoucheng | Shandong | Migrant women | 2698 | ELISA | — | 38 | 2698 |
| Shu HY, 2010[^14^](#_ENREF_14) | 2005-2008 | Zoucheng | Shandong | Migrant women | 3091 | — | ELISA | 44 | 3091 |
| Su SL, 2011[^15^](#_ENREF_15) | 2008 | Shandong | Shandong | Migrant women | 7102 | YES | — | 121 | 7102 |
| Xu WX, 2009[^24^](#_ENREF_24) | 2004-2007 | Jiaxing | Zhejiang | Migrant women | 791 | RPR | TPPA | 7 | 791 |
| Zhang SY, 2009[^31^](#_ENREF_31) | 2007 | Jucounty | Shandong | Migrant women | 742 | RPR | — | 10 | 742 |
| Zhang YQ, 2008[^34^](#_ENREF_34) | 2005-2007 | Zoucheng | Shandong | Migrant women | 2698 | — | ELISA | 38 | 2698 |
| Zhou QY, 2010[^348^](#_ENREF_348) | 2009-2009 | Dongguan | Guangdong | Migrant women | 3509 | — | — | 3 | 3509 |
| Guo WQ, 2010[^38^](#_ENREF_38) | 2006 | Lanzhou | Gansu | Truck drivers | 400 | ELISA | — | 4 | 400 |
| Liao MZ, 2008[^42^](#_ENREF_42) | 2007 | Jinan | Shandong | Truck drivers | 377 | TRUST | TPPA | 0 | 377 |
| Liao MZ, 2010[^43^](#_ENREF_43) | 2009 | Jinan | Shandong | Truck drivers | 425 | TRUST | TPPA | 20 | 422 |
| Liu XZ, 2006[^44^](#_ENREF_44) | 2004 | Shandong | Shandong | Truck drivers | 720 | TRUST | TPPA | 0 | 720 |
| Nong LP, 2005[^46^](#_ENREF_46) | — | — | — | Truck drivers | 368 | ELISA | TPPA | 4 | 368 |
| Wang YC, 2007[^47^](#_ENREF_47) | 2006 | Dangyang | Hubei | Truck drivers | 305 | — | — | 2 | 305 |
| Yi JF , 2010[^52^](#_ENREF_52) | 2009 | Yichun | Jiangsu | Truck drivers | 400 | — | — | 0 | 400 |
| Chen XS, 2006[^36^](#_ENREF_36) | 2000 | Tongling | Anhui | Truck drivers | 550 | RPR | TPHA | 4 | 550 |
| Ge FQ, 2007[^55^](#_ENREF_55) | 2005 | Sichuan | Sichuan | Construction workers | 599 | RPR | TPHA | 8 | 571 |
| He B, 2012[^56^](#_ENREF_56) | 2011 | Pinxiang | Guangxi | Construction workers | 405 | ELISA | TPPA | 16 | 405 |
| He L, 2012[^57^](#_ENREF_57) | 2010 | Shenzhen | Guangdong | Construction workers | 101 | TRUST | — | 3 | 101 |
| Hu XQ, 2011[^58^](#_ENREF_58) | 2010 | Baoji | Shaanxi | Construction workers | 400 | ELISA | RPR | 6 | 400 |
| Liu HX, 2011[^59^](#_ENREF_59) | 2010 | Changping | Beijing | Construction workers | 449 | ELISA | RPR | 1 | 449 |
| Mou XL, 2012[^60^](#_ENREF_60) | 2010 | Wuxi | Jiangsu | Construction workers | 1703 | ELISA | RPR/TRUST | 0 | 481 |
| Sheng MY, 2010[^61^](#_ENREF_61) | 2008 | Haining,Jiaxin | Zhejiang | Construction workers | 231 | RPR | — | 3 | 231 |
| **Table S5b Characteristics description of studies included in meta-analysis of syphilis prevalence (Cont’d)** | | | | | | | |  |  |
| **First Author &** | **Study** | **Study Location** |  | **Subgroup** | **Sample** | **Presumptive** | **Confirmatory** | **No. of** | **No. of people** |
| **Published Year** | **Period** | **City/county** | **Province** | **Population** | **Size** | **test(s)** | **test(s)*** | **infection** | **tested** |
| Syphilis (Cont’d) |  |  |  |  |  |  |  |  |  |
| Sun BW, 1997[^349^](#_ENREF_349) | 1996-1997 | Jiangyan | Jiangsu | Construction workers | 5892 | — | — | 3 | 5892 |
| Wang CH, 2012[^63^](#_ENREF_63) | 2011 | huanggang | Hubei | Construction workers | 332 | TRUST | — | 9 | 301 |
| Xia DY, 2011[^64^](#_ENREF_64) | 2009 | Beijing | Beijing | Construction workers | 3161 | RPR | — | 16 | 3161 |
| Zhang P, 2011[^66^](#_ENREF_66) | — | Kuancheng, Changchun | Jilin | Construction workers | 400 | RPR | TRUSt | 0 | 400 |
| Zhang Y, 2012[^67^](#_ENREF_67) | 2010 | Ningbo | Zhejiang | Construction workers | 925 | ELISA | RPR | 10 | 925 |
| He B, 2012[^56^](#_ENREF_56) | 2011 | Pinxiang | Guangxi | Factory workers | 403 | ELISA | TPPA | 10 | 403 |
| Huang RR, 2008[^69^](#_ENREF_69) | — | Jinshan District | Shanghai | Factory workers | 310 | TRUST | TPPA | 0 | 310 |
| Mou XL, 2012[^60^](#_ENREF_60) | 2010 | Wuxi | Jiangsu | Factory workers | 1703 | ELISA | RPR/TRUST | 0 | 399 |
| Pu Y, 2011[^70^](#_ENREF_70) | 2010-2011 | Jingjiang | Jiangsu | Factory workers | 469 | — | — | 1 | 469 |
| Wei W, 2012[^71^](#_ENREF_71) | 2012 | Shanghai | Shanghai | Factory workers | 404 | ELISA | TRUST | 1 | 404 |
| Xia DY, 2011[^64^](#_ENREF_64) | 2009 | Beijing | Beijing | Factory workers | 59 | RPR | — | 0 | 59 |
| Zhang Y, 2012[^67^](#_ENREF_67) | 2010 | Ningbo | Zhejiang | Factory workers | 1029 | ELISA | RPR | 6 | 1029 |
| Mou XL, 2012[^60^](#_ENREF_60) | 2010 | Wuxi | Jiangsu | Restaurant attendants | 1703 | ELISA | RPR/TRUST | 0 | 400 |
| Zhang Y, 2012[^67^](#_ENREF_67) | 2010 | Ningbo | Zhejiang | Restaurant attendants | 1025 | ELISA | RPR | 8 | 1025 |
| Cai T, 2010[^73^](#_ENREF_73) |  | Dalian | Liaoning | Unspecified job nature | 60 | TRUST | — | 0 | 60 |
| Cai W, 2005[^74^](#_ENREF_74) | 2003-2004 | Heze | Shandong | Unspecified job nature | 2137 | — | — | 22 | 2137 |
| Chen SH, 2012[^76^](#_ENREF_76) | 2010 | Foshan | Guangdong | Unspecified job nature | 1611 | RPR | TRUST | 9 | 1611 |
| Cui DZ, 2012[^77^](#_ENREF_77) | 2012 | Lanzhou | Gansu | Unspecified job nature | 400 | ELISA | TRUST | 2 | 400 |
| Dai M, 2007[^350^](#_ENREF_350) |  | Jiulongpo | Chongqing | Unspecified job nature | 1026 | — | — | 3 | 1026 |
| Ding XB, 2007[^78^](#_ENREF_78) | 2006-2006 | Yuzhong, Jiulongpo | Chongqing | Unspecified job nature | 1215 | RPR | TP | 5 | 1215 |
| Gu MD, 2009[^80^](#_ENREF_80) | 2007 | Pingjiang | Hunan | Unspecified job nature | 576 | — | — | 20 | 576 |
| Guo WG, 2009[^82^](#_ENREF_82) | 2008 | Beihai | Guangxi | Unspecified job nature | 255 | RPR | YES | 3 | 255 |
| **Table S5b Characteristics description of studies included in meta-analysis of syphilis prevalence (Cont’d)** | | | | | | | |  |  |
| **First Author &** | **Study** | **Study Location** |  | **Subgroup** | **Sample** | **Presumptive** | **Confirmatory** | **No. of** | **No. of people** |
| **Published Year** | **Period** | **City/county** | **Province** | **Population** | **Size** | **test(s)** | **test(s)*** | **infection** | **tested** |
| Syphilis (Cont’d) |  |  |  |  |  |  |  |  |  |
| Hong PK, 2010[^86^](#_ENREF_86) | 2010 | Jinjiang | Fujian | Unspecified job nature | 400 | — | — | 3 | 400 |
| Huang T, 2012[^88^](#_ENREF_88) | 2009 | Xichang | Sichuan | Unspecified job nature | 173 | ELISA | — | 3 | 173 |
| Huang ZL, 2013[^91^](#_ENREF_91) | 2011 | Changsha | Hunan | Unspecified job nature | 1093 | RPR | TPPA | 6 | 1093 |
| Jiang CH, 2012a[^92^](#_ENREF_92) | 2009 | Jinshan District | Shanghai | Unspecified job nature | 400 | ELISA,TRUST | — | 0 | 400 |
| Jiang CH, 2012b[^92^](#_ENREF_92) | 2010 | Jinshan District | Shanghai | Unspecified job nature | 405 | ELISA,TRUST | — | 0 | 405 |
| Jiang CH, 2012c[^92^](#_ENREF_92) | 2011 | Jinshan District | Shanghai | Unspecified job nature | 400 | ELISA,TRUST | — | 3 | 400 |
| Jiang JH, 2012[^93^](#_ENREF_93) | 2012 | Jiangyin | Jiangsu | Unspecified job nature | 400 | ELISA | RPR/TRUST | 5 | 400 |
| Jiang YF, 2010[^94^](#_ENREF_94) | 2009 | Hangzhou | Zhejiang | Unspecified job nature | 441 | ELISA-1 | ELISA-2 | 9 | 441 |
| Jing LH, 2010[^95^](#_ENREF_95) | 2009 | Yangquan | Shanxi | Unspecified job nature | 409 | RPR | TPPA | 1 | 409 |
| Li XJ, 2011[^102^](#_ENREF_102) | 2009 | Hefei | Anhui | Unspecified job nature | 1657 |  |  | 6 | 1657 |
| Liao MZ, 2008[^42^](#_ENREF_42) | 2007 | Jinan | Shandong | Unspecified job nature | 818 | TRUST | TPPA | 0 | 818 |
| Liao MZ, 2010[^43^](#_ENREF_43) | 2009 | Jinan | Shandong | Unspecified job nature | 1128 | TRUST | TPPA | 4 | 1128 |
| Lin X, 2011[^103^](#_ENREF_103) | 2010 | Fujian | Fujian | Unspecified job nature | 1705 | RPR/TRUST |  | 10 | 1705 |
| Lin YT, 2012[^104^](#_ENREF_104) | 2010 | Changle,Jinjiang,Meilie | Fujian | Unspecified job nature | 4167 | ELISA | RPR/TRUST | 14 | 4098 |
| Liu SZ, 2012[^106^](#_ENREF_106) | 2010 | Xuanhan | Sichuan | Unspecified job nature | 400 | ELISA | RPR | 8 | 400 |
| Liu XF, 2000[^351^](#_ENREF_351) | 1999 | Daishan | Zhejiang | Unspecified job nature | 98 | RPR | TPHA | 8 | 98 |
| Liu XZ, 2006[^44^](#_ENREF_44) | 2004 | Shandong | Shandong | Unspecified job nature | 832 | TRUST | TPPA | 4 | 832 |
| Lu JJ, 2011[^107^](#_ENREF_107) | 2011 | Jiangyin | Jiangsu | Unspecified job nature | 400 | ELISA | RPR,TRUST | 1 | 400 |
| Ma YL, 2010[^352^](#_ENREF_352) | 2006 | Pingdu | Shandong | Unspecified job nature | 396 | RPR | TPHA | 6 | 396 |
| Meng XJ, 2010[^110^](#_ENREF_110) | 2008 | Jilin, Baicheng, Tonghua,Baishan | Jilin | Unspecified job nature | 2113 | — | — | 0 | 710 |
| Meng XJ, 2010[^111^](#_ENREF_111) | — | Hunan | Hunan | Unspecified job nature | 2233 | RPR | TP | 33 | 2233 |
| **Table S5b Characteristics description of studies included in meta-analysis of syphilis prevalence (Cont’d)** | | | | | | | |  |  |
| **First Author &** | **Study** | **Study Location** |  | **Subgroup** | **Sample** | **Presumptive** | **Confirmatory** | **No. of** | **No. of people** |
| **Published Year** | **Period** | **City/county** | **Province** | **Population** | **Size** | **test(s)** | **test(s)*** | **infection** | **tested** |
| Syphilis (Cont’d) |  |  |  |  |  |  |  |  |  |
| Mou XL, 2012[^60^](#_ENREF_60) | 2010 | Wuxi | Jiangsu | Unspecified job nature | 1703 | ELISA | RPR/TRUST | 1 | 1687 |
| Mou XL, 2012[^60^](#_ENREF_60) | 2010 | Wuxi | Jiangsu | Unspecified job nature | 1703 | ELISA | RPR/TRUST | 1 | 407 |
| Mou XL, 2011[^112^](#_ENREF_112) | 2010 | Wuxi | Jiangsu | Unspecified job nature | 400 | ELISA | TRUST | 0 | 400 |
| Pan XH, 2002[^113^](#_ENREF_113) | 2001 | Hainan | Hainan | Unspecified job nature | 251 | RPR | TPPA | 4 | 251 |
| Qian ZY, 2011[^115^](#_ENREF_115) | 2010 | Xinghua | Jiangsu | Unspecified job nature | 511 | YES | TPHA | 11 | 511 |
| Qiu JQ, 2008[^116^](#_ENREF_116) | 2004-2006 | Kaihua | Zhejiang | Unspecified job nature | 1557 | — | RPR | 0 | 1557 |
| Qiu JQ, 2011[^117^](#_ENREF_117) | 2010 | Kaihua | Zhejiang | Unspecified job nature | 873 | RPR | — | 0 | 873 |
| Ruan JJ, 2010[^118^](#_ENREF_118) | 2009 - 2009 | Yiwu | Zhejiang | Unspecified job nature | 402 | RPR | — | 0 | 400 |
| Shi XJ, 2011[^119^](#_ENREF_119) | 2010 | Ningbo | Zhejiang | Unspecified job nature | 800 | ELISA | RPR | 1 | 793 |
| Su MF, 2009[^353^](#_ENREF_353) | — | Yuhuan | Zhejiang | Unspecified job nature | 622 | ELISA | TPPA | 4 | 622 |
| Tan Y, 2011[^121^](#_ENREF_121) | 2010 | Zhongshan | Guangdong | Unspecified job nature | 1600 | ELISA | TRUST | 7 | 1600 |
| Wang YL, 2006[^127^](#_ENREF_127) | 2006 | Yuzhong and Jiulongpo | Chongqing | Unspecified job nature | 2047 | RPR | TPHA | 13 | 2047 |
| Xia DY, 2011[^64^](#_ENREF_64) | 2009 | Beijing | Beijing | Unspecified job nature | 4187 | RPR | — | 16 | 4187 |
| Xia DY, 2011[^64^](#_ENREF_64) | 2009 | Beijing | Beijing | Unspecified job nature | 967 | RPR | — | 0 | 967 |
| Xing AH, 2007[^129^](#_ENREF_129) | 2006 | Shaanxi | Shaanxi | Unspecified job nature | 2753 | RPR | TPPA, ELISA | 10 | 2753 |
| Xu Y, 2010[^131^](#_ENREF_131) | 2009 | Chongming | Shanghai | Unspecified job nature | 400 | — | — | 1 | 400 |
| Xue LJ, 2011[^132^](#_ENREF_132) | 2010 | Kunshan | Jiangsu | Unspecified job nature | 400 | — | — | 3 | 400 |
| Yan ZZ, 2007[^133^](#_ENREF_133) | 2006 | Chongyang | Hubei | Unspecified job nature | 400 | RPR | — | 16 | 400 |
| Yang MH, 2011[^135^](#_ENREF_135) | 2010 | Sanming | Fujian | Unspecified job nature | 402 | ELISA | RPR/TRUST | 2 | 402 |
| Ye W, 2003[^138^](#_ENREF_138) | 2002 | Shanghai | Shanghai | Unspecified job nature | 1128 | TRUST | TPPA | 6 | 1041 |
| Yu XF, 2011a[^141^](#_ENREF_141) | 2006 | Chongqing | Chongqing | Unspecified job nature | 604 | — | — | 0 | 604 |
| Yu XF, 2011b[^141^](#_ENREF_141) | 2007 | Chongqing | Chongqing | Unspecified job nature | 606 | — | — | 0 | 606 |
| **Table S5b Characteristics description of studies included in meta-analysis of syphilis prevalence (Cont’d)** | | | | | | | |  |  |
| **First Author &** | **Study** | **Study Location** |  | **Subgroup** | **Sample** | **Presumptive** | **Confirmatory** | **No. of** | **No. of people** |
| **Published Year** | **Period** | **City/county** | **Province** | **Population** | **Size** | **test(s)** | **test(s)*** | **infection** | **tested** |
| Syphilis (Cont’d) |  |  |  |  |  |  |  |  |  |
| Yu XF, 2011c[^141^](#_ENREF_141) | 2008 | Chongqing | Chongqing | Unspecified job nature | 718 | — | — | 3 | 718 |
| Yu XF, 2011d[^141^](#_ENREF_141) | 2009 | Chongqing | Chongqing | Unspecified job nature | 410 | — | — | 0 | 410 |
| Yuan JM, 2012[^354^](#_ENREF_354) | — | Qantong | Jiangsu | Unspecified job nature | 380 | RPR | TPPA | 6 | 380 |
| Zang DA, 2008[^142^](#_ENREF_142) | 2006-2007 | Qheyang | Jiangsu | Unspecified job nature | 3554 | RPR | TPPA | 3 | 3554 |
| Zhang Y, 2012[^67^](#_ENREF_67) | 2010 | Ningbo | Zhejiang | Unspecified job nature | 4002 | ELISA | RPR | 29 | 4002 |
| Zhang Y, 2012[^67^](#_ENREF_67) | 2010 | Ningbo | Zhejiang | Unspecified job nature | 1023 | ELISA | RPR | 5 | 1023 |
| Zhu JQ, 2012[^146^](#_ENREF_146) | 2010 | Jinzhou | Zhejiang | Unspecified job nature | 403 | ELISA | — | 4 | 403 |
| Zhu YY, 2012[^147^](#_ENREF_147) | 2009 | Beijing | Beijing | Unspecified job nature | 4187 | RPR | — | 15 | 4187 |
| He N, 2005[^83^](#_ENREF_83) | — | Shanghai | Shanghai | Unspecified job nature | 1086 | — | TPHA | 10 | 986 |
| Hesketh T, 2006[^85^](#_ENREF_85) | 2004 | Hangzhou | Zhejiang | Unspecified job nature | 4148 | PA | VDRL | 20 | 4148 |
| Pan XH, 2013[^114^](#_ENREF_114) | 2010 | Nanjing | Zhejiang | Unspecified job nature | 17377 | RPR | TPPA | 70 | 12694 |
| Chen JX, 2005[^148^](#_ENREF_148) | 20022003 | Shenzhen | Guangdong | Pregnant women | 4365 | TRUST | TPHA | 3 | 315 |
| Cheng JQ, 2008[^355^](#_ENREF_355) | 2003-2005 | Shenzhen | Guangdong | Pregnant women | 418871 | TRUST | TPPA | 759 | 185979 |
| Huang ZM, 2004[^356^](#_ENREF_356) | 1999-2002 | Shenzhen | Guangdong | Pregnant women | 10842 | TRUST | TPPA | 47 | 10842 |
| Hu YM, 2011[^357^](#_ENREF_357) | 2005-2007 | Jingning | Zhejiang | Pregnant women | 4911 | TRUST | —— | 15 | 89 |
| Li SH, 2012a[^152^](#_ENREF_152) | 2007 | Beijing | Beijing | Pregnant women | 26819 | CMIA | ABDSTT | 6 | 6730 |
| Li SH, 2012b[^152^](#_ENREF_152) | 2008 | Beijing | Beijing | Pregnant women | 26819 | CMIA | ABDSTT | 13 | 6869 |
| Li SH, 2012c[^152^](#_ENREF_152) | 2009 | Beijing | Beijing | Pregnant women | 26819 | CMIA | ABDSTT | 10 | 7076 |
| Li SH, 2012d[^152^](#_ENREF_152) | 2010 | Beijing | Beijing | Pregnant women | 26819 | CMIA | ABDSTT | 12 | 6144 |
| Liao HL, 2008[^358^](#_ENREF_358) | 2003-2007 | Shenzhen | Guangdong | Pregnant women | 10703 | RPR | TPPA | 40 | 10703 |
| Nong LP, 2011[^154^](#_ENREF_154) | 2009-2010 | Pingxiang | Guangxi | Pregnant women | 800 | TRUST | — | 1 | 117 |
| Fang Q, 2005[^173^](#_ENREF_173) | — | Shenzhen | Guangdong | FSWs | 55 | — | — | 5 | 55 |
| Liao CQ, 2011[^196^](#_ENREF_196) | — | Hongkou | Shanghai | FSWs | 401 | RPR | TPPA | 22 | 401 |
| Liu SH, 2009[^203^](#_ENREF_203) | 2008 | Shizuishan | Ningxia | FSWs | 366 | RPR | — | 0 | 339 |
| **Table S5b Characteristics description of studies included in meta-analysis of syphilis prevalence (Cont’d)** | | | | | | | |  |  |
| **First Author &** | **Study** | **Study Location** |  | **Subgroup** | **Sample** | **Presumptive** | **Confirmatory** | **No. of** | **No. of people** |
| **Published Year** | **Period** | **City/county** | **Province** | **Population** | **Size** | **test(s)** | **test(s)*** | **infection** | **tested** |
| Syphilis (Cont’d) |  |  |  |  |  |  |  |  |  |
| Pan XL, 2008[^209^](#_ENREF_209) | 2005-2006 | Baise | Guangxi | FSWs | 253 | RPR | — | 0 | 176 |
| Pan XL, 2010[^211^](#_ENREF_211) | 2006-2008 | Baise | Guangxi | FSWs | 269 | RPR | — | 0 | 220 |
| Qin CZ, 2012[^214^](#_ENREF_214) | 2010-2011 | Jiangyan | Jiangsu | FSWs | 817 | ELISA | TRUST | 16 | 347 |
| Tang X, 2012a[^221^](#_ENREF_221) | 2008 | Shanghai | Shanghai | FSWs | 244 | RPR | TPPA | 10 | 244 |
| Tang X, 2012b[^221^](#_ENREF_221) | 2008 | Shanghai | Shanghai | FSWs | 360 | RPR | TPPA | 15 | 360 |
| Tang ZL, 2011[^222^](#_ENREF_222) | 2010 | Qingdao | Shandong | FSWs | 322 | RPR | TPPA | 0 | 174 |
| Xiang SB, 2010[^239^](#_ENREF_239) | 2009 | Hongjiang | Hunan | FSWs | 401 | — | — | 0 | 63 |
| Xu HQ, 2008[^243^](#_ENREF_243) | 2006 | Jiaxing | Zhejiang | FSWs | 151 | — | — | 0 | 142 |
| Zhang QQ, 2012[^359^](#_ENREF_359) | 2009 | Changzhou | Jiangsu | FSWs | 1806 | ELISA | RPR | 114 | 1349 |
| Zhang YX, 2011[^258^](#_ENREF_258) | 2007 | Liuzhou | Guangxi | FSWs | 403 | RPR | — | 1 | 90 |
| Zhao AL, 2011[^259^](#_ENREF_259) | 2009 | Lanzhou | Gansu | FSWs | 350 | TRUST | — | 20 | 174 |
| Zheng J, 2012[^262^](#_ENREF_262) | 2009-2010 | Anshan | Liaoning | FSWs | 400 | RPR | TPPA | 0 | 31 |
| Chen XS, 2012[^360^](#_ENREF_360) | 2003 | 8 cities | 8 Provinces | FSWs | 7118 | ELISA | TRUST | 273 | 5246 |
| Liao MZ, 2011[^361^](#_ENREF_361) | 2006-2008 | Qingdao | Shandong | FSWs | 1209 | RPR | TPPA | 10 | 414 |
| Hong Y, 2009[^362^](#_ENREF_362) | 2004 | Guangxi | Guangxi | FSWs | 151 | RPR | TPPA | 4 | 147 |
| Yang Y, 2011a[^248^](#_ENREF_248) | 2008 | Minhang | Shanghai | FSWs | 411 | — | TPHA | 26 | 382 |
| Yang Y, 2011b[^248^](#_ENREF_248) | 2009 | Minhang | Shanghai | FSWs | 411 | — | TPHA | 7 | 382 |
| Ding XB, 2010[^273^](#_ENREF_273) | 2008 | Shongqing | Chongqing | MSM | 743 | RPR | TPPA | 10 | 163 |
| He B, 2011[^363^](#_ENREF_363) | 2010 | Haikou | Hainan | MSM | 248 | ELISA | RPR | 14 | 97 |
| Lan GH, 2009[^276^](#_ENREF_276) | 2008 | Guangxi | Guangxi | MSM | 179 | RPR/TRUST | — | 14 | 179 |
| Liang L, 2009[^277^](#_ENREF_277) | 2008 | Shijiazhuang | Hebei | MSM | 450 | TRUST | TPPA | 14 | 200 |
| Liu PL, 2010[^278^](#_ENREF_278) | 2008 | Wuhan | Hubei | MSM | 456 | RPR | TPPA | 38 | 237 |
| Liu ZQ, 2009[^279^](#_ENREF_279) | 2006-2007 | Sianjin | Tianjin | MSM | 89 | RPR | — | 16 | 83 |
| Mei L, 2009[^280^](#_ENREF_280) | 2008-2008 | Taiyuan | Shanxi | MSM | 273 | RPR | TPPA | 26 | 184 |
| **Table S5b Characteristics description of studies included in meta-analysis of syphilis prevalence (Cont’d)** | | | | | | | |  |  |
| **First Author &** | **Study** | **Study Location** |  | **Subgroup** | **Sample** | **Presumptive** | **Confirmatory** | **No. of** | **No. of people** |
| **Published Year** | **Period** | **City/county** | **Province** | **Population** | **Size** | **test(s)** | **test(s)*** | **infection** | **tested** |
| Syphilis (Cont’d) |  |  |  |  |  |  |  |  |  |
| Wang Y, 2009[^286^](#_ENREF_286) | 2007 | Mianyang | Sichuan | MSM | 111 | TPPA | ELISA | 7 | 43 |
| Wen F, 2010[^288^](#_ENREF_288) | 2008 | Guangzhou | Guangdong | MSM | 452 | RPR | TPPA | 24 | 290 |
| Feng TJ, 2008[^364^](#_ENREF_364) | 2005-2007 | Shenzhen | Guangdong | MSM | 1313 | TRUST | TPPA | 9 | 44 |
| He Q, 2009[^274^](#_ENREF_274) | 2006 | Guangzhou | Guangdong | MSM | 380 | ELISA | RPR | 56 | 350 |
| Ruan YH, 2007[^283^](#_ENREF_283) | 2005 | Seijing | Beijing | MSM | 526 | ELISA | TPPA | 19 | 188 |
| Ruan YH, 2009[^365^](#_ENREF_365) | — | Seijing | Beijing | MSM | 551 | ELISA | PPAT | 76 | 356 |
| Wang B, 2013[^284^](#_ENREF_284) | 2008-2009 | Beijing | Beijing | MSM | 307 | ELISA | PPAT | 62 | 307 |
| Xiao Y, 2009[^289^](#_ENREF_289) | 2007 | Shongqing | Chongqing | MSM | 1692 | RPR | TPPA | 29 | 373 |
| Xiao Y, 2010[^290^](#_ENREF_290) | 2007 | Several sites | Several Provinces | MSM | 4657 | RPR | TPPA | 134 | 1301 |
| Zou HC, 2010[^298^](#_ENREF_298) | 2007 | Seijing | Beijing | MSM | 429 | ELISA | TPPA | 33 | 264 |
| Zeng YM, 2005[^300^](#_ENREF_300) | 2000-2003 | Quzhou | Fujian | DUs | 1252 | RPR | YES | 73 | 633 |
| Fu DL, 2009[^306^](#_ENREF_306) | 2009 | Nanchan | Hubei | DUs | 400 | RPR | YES | 49 | 400 |
| Huang F, 2005[^307^](#_ENREF_307) | — | Shenzhen | Guangdong | DUs | 198 | — | — | 9 | 86 |
| Li GY, 2009[^366^](#_ENREF_366) | 2006 | beijing | Beijing | DUs | 720 | ELISA | — | 44 | 531 |
| Li XM, 2002[^314^](#_ENREF_314) | 1999 | Lanzhou | Gansu | DUs | 871 | — | — | 86 | 871 |
| Liang ZJ, 2005[^315^](#_ENREF_315) | 2003-2004 | Guangzhou | Guangdong | DUs | 739 | TPPA | YES | 56 | 388 |
| Li XM, 2002[^314^](#_ENREF_314) | 2000 | lanzhou | Gansu | DUs | 1356 | — | — | 15 | 89 |
| Lin ZL,2008[^367^](#_ENREF_367) | 2007 | Sanya | Hainan | DUs | 395 | — | — | 6 | 88 |
| Liu SF,2008[^319^](#_ENREF_319) | 2005-2007 | Changzhou | Jiangsu | DUs | 855 | — | — | 19 | 328 |
| Chen LP,2008[^301^](#_ENREF_301) | 2007 | Qingyuan | Guangdong | DUs | 1073 | TPPA | YES | 9 | 73 |
| Luo WQ, 2012[^323^](#_ENREF_323) | 2010-2012 | Taishan | Guangdong | DUs | 1386 | ELISA | TRUST | 14 | 206 |
| Qiu H, 2012[^325^](#_ENREF_325) | 2010 | Kunming | Yunnan | DUs | 872 | RPR | TPPA | 14 | 162 |
| Tao XY, 2004[^328^](#_ENREF_328) | 2003 | Shenzhen | Guangdong | DUs | 198 | YES | — | 5 | 49 |
| **Table S5b Characteristics description of studies included in meta-analysis of syphilis prevalence (Cont’d)** | | | | | | | |  |  |
| **First Author &** | **Study** | **Study Location** |  | **Subgroup** | **Sample** | **Presumptive** | **Confirmatory** | **No. of** | **No. of people** |
| **Published Year** | **Period** | **City/county** | **Province** | **Population** | **Size** | **test(s)** | **test(s)*** | **infection** | **tested** |
| Syphilis (Cont’d) |  |  |  |  |  |  |  |  |  |
| Wang Y, 2007[^333^](#_ENREF_333) | 2004-2006 | Jining | Shandong | DUs | 292 | RPR | TPPA | 8 | 183 |
| Wu QH, 2010a[^335^](#_ENREF_335) | 2007 | Taizhou | Zhejiang | DUs | 300 | ELISA | — | 5 | 251 |
| Wu QH, 2010b[^335^](#_ENREF_335) | 2008 | Taizhou | Zhejiang | DUs | 363 | ELISA | — | 10 | 362 |
| Wu QH, 2010c[^335^](#_ENREF_335) | 2009 | Taizhou | Zhejiang | DUs | 444 | ELISA | — | 30 | 440 |
| Xie HY,2005[^336^](#_ENREF_336) | 2000-2003 | Quanzhou | Fujian | DUs | 933 | PRP | TPPA | 107 | 440 |
| Li DL,2012[^311^](#_ENREF_311) | 2007 | Beijing | Beijing | DUs | 128 | ELISA | — | 17 | 128 |

| **Table S5c Characteristics description of studies included in meta-analysis of gonorrhea prevalence** | | | | | | | |  |  |
| --- | --- | --- | --- | --- | --- | --- | --- | --- | --- |
| Gonorrhea |  |  |  |  |  |  |  |  |  |
| Zhou QY, 2010[^348^](#_ENREF_348) | 2009 | Dongguan | Guangdong | Migrant women | 3509 | — | — | 127 | 3509 |
| Zhou YJ,2012[^368^](#_ENREF_368) | 2012 | Hengyang | Hunan | Migrant women | 100 | — | — | 0 | 100 |
| Chen XS, 2006[^36^](#_ENREF_36) | 2000 | Tongling | Anhui | Truck drivers | 550 | — | — | 43 | 530 |
| Sun BW, 1997[^349^](#_ENREF_349) | 1996-1997 | Jiangyan | Jiangsu | Construction workers | 5892 | — | — | 184 | 5892 |
| Zhao R, 2005[^68^](#_ENREF_68) | — | Gejiu City | Yunnan | Miners | 232 | — | — | 1 | 182 |
| Cai W, 2005[^74^](#_ENREF_74) | 2003-2004 | Heze | Shandong | Unspecified job nature | 2137 | Colloidal Gold | — | 17 | 2137 |
| He N, 2005[^83^](#_ENREF_83) | — | Shanghai | Shanghai | Unspecified job nature | 1086 | LCR | — | 5 | 986 |
| Lei JG, 2010[^369^](#_ENREF_369) | 2008 | Huidong | Sichuan | Unspecified job nature | 1741 | — | — | 7 | 518 |
| Liu XF, 2000[^351^](#_ENREF_351) | 1999 | Daishan | Zhejiang | Unspecified job nature | 98 | Gram Staining | — | 24 | 98 |
| Ma YL,2010[^352^](#_ENREF_352) | 2006 | Pingdu | Shandong | Unspecified job nature | 396 | Oxidase, Glucolysis | — | 6 | 396 |
| Wang W, 2010a[^370^](#_ENREF_370) | 1999-2000 | 18 provinces | 18 provinces | Unspecified job nature | 272 | LCR | — | 0 | 234 |
| Wang W, 2010b[^370^](#_ENREF_370) | 1999-2000 | 18 provinces | 18 provinces | Unspecified job nature | 213 | LCR | — | 9 | 198 |
| Ye W, 2003[^138^](#_ENREF_138) | 2002 | Shanghai | Shanghai | Unspecified job nature | 1128 | LCR, MEIA | — | 8 | 1128 |
| Hong Y, 2009[^362^](#_ENREF_362) | 2004 | Guangxi | Guangxi | FSWs | 151 | YES | — | 17 | 147 |
| Wu JP, 2006[^371^](#_ENREF_371) | 2002-2004 | Jiangmen | Guangdong | DUs | 326 | — | — | 0 | 189 |

| **Table S5d Characteristics description of studies included in meta-analysis of chlamydia prevalence** | | | | | | | |  |  |
| --- | --- | --- | --- | --- | --- | --- | --- | --- | --- |
| **First Author &** | **Study** | **Study Location** |  | **Subgroup** | **Sample** | **Presumptive** | **Confirmatory** | **No. of** | **No. of people** |
| **Published Year** | **Period** | **City/county** | **Province** | **Population** | **Size** | **test(s)** | **test(s)*** | **infection** | **tested** |
| Chlamydia |  |  |  |  |  |  |  |  |  |
| Zhou QY, 2010[^348^](#_ENREF_348) | 2009 | Dongguan | Guangdong | Migrant women | 3509 | — | — | 9 | 3509 |
| Chen XS, 2006[^36^](#_ENREF_36) | 2000 | Tongling | Anhui | Truck drivers | 550 | — | — | 56 | 530 |
| Zhao R, 2005[^68^](#_ENREF_68) | — | Gejiu City | Yunnan | Miners | 232 | — | — | 17 | 182 |
| Cai W, 2005[^74^](#_ENREF_74) | 2003-2004 | Heze | Shandong | Unspecified job nature | 2137 | — | Colloidal Gold | 76 | 2137 |
| Ye W, 2003[^138^](#_ENREF_138) | 2002 | Shanghai | Shanghai | Unspecified job nature | 1128 | — | — | 38 | 1128 |
| He N, 2005[^83^](#_ENREF_83) | — | Shanghai | Shanghai | Unspecified job nature | 1086 | — | LCR | 35 | 986 |
| Wang W, 2010a[^370^](#_ENREF_370) | 1999-2000 | 18 provinces | 18 provinces | Unspecified job nature | 272 | — | LCR | 4 | 234 |
| Wang W, 2010b[^370^](#_ENREF_370) | 1999-2000 | 18 provinces | 18 provinces | Unspecified job nature | 213 | — | LCR | 0 | 198 |
| Zhang QQ, 2012[^359^](#_ENREF_359) | 2009 | Changzhou | Jiangsu | FSWs | 1806 | PCR | — | 103 | 656 |
| Hong Y, 2009[^362^](#_ENREF_362) | 2004 | Guangxi | Guangxi | FSWs | 151 | RAT | — | 20 | 147 |

| **Table S5e Characteristics description of studies included in meta-analysis of genital warts prevalence** | | | | | | | |  |  |
| --- | --- | --- | --- | --- | --- | --- | --- | --- | --- |
| **First Author &** | **Study** | **Study Location** |  | **Subgroup** | **Sample** | **Presumptive** | **Confirmatory** | **No. of** | **No. of people** |
| **Published Year** | **Period** | **City/county** | **Province** | **Population** | **Size** | **test(s)** | **test(s)*** | **infection** | **tested** |
| Genital warts |  |  |  |  |  |  |  |  |  |
| Zhou YJ, 2012[^368^](#_ENREF_368) | 2012 | Hengyang | Hunan | Migrant women | 100 | — | — | 2 | 100 |
| Zhou QY, 2010[^348^](#_ENREF_348) | 2009 | Dongguan | Guangdong | Migrant women | 3509 | — | — | 42 | 3509 |
| Sun BW, 1997[^349^](#_ENREF_349) | 1996-1997 | Jiangyan | Jiangsu | Construction workers | 5892 | — | — | 25 | 5892 |
| Lei JG, 2010[^369^](#_ENREF_369) | 2008 | Huidong | Sichuan | Unspecified job nature | 1741 | — | — | 16 | 518 |
| Liu XF, 2000[^351^](#_ENREF_351) | 1999 | Daishan | Zhejiang | Unspecified job nature | 98 | — | — | 1 | 98 |
| Ma YL, 2010[^352^](#_ENREF_352) | 2006 | Pingdu | Shandong | Unspecified job nature | 396 | — | — | 15 | 396 |

| **Table S5f Characteristics description of studies included in meta-analysis of human papillomavirus (HPV) prevalence** | | | | | | | |  |  |
| --- | --- | --- | --- | --- | --- | --- | --- | --- | --- |
| **First Author &** | **Study** | **Study Location** |  | **Subgroup** | **Sample** | **Presumptive** | **Confirmatory** | **No. of** | **No. of people** |
| **Published Year** | **Period** | **City/county** | **Province** | **Population** | **Size** | **test(s)** | **test(s)*** | **infection** | **tested** |
| HPV |  |  |  |  |  |  |  |  |  |
| Zhou YJ, 2012[^368^](#_ENREF_368) | 2012 | Hengyang | Hunan | Migrant women | 100 | — | — | 17 | 100 |
| Wu ZL, 2012[^372^](#_ENREF_372) | 2010-2011 | Shenzhen | Guangdong | Factory workers | 5260 | GENE | — | 364 | 5260 |
| Yao CL, 2012[^373^](#_ENREF_373) | 2009-2011 | Hangzhou | Zhejiang | Unspecified job nature | 1198 | — | — | 196 | 1198 |
| Chen XX, 2011[^272^](#_ENREF_272) | 2010 | Beijing | Beijing | MSM | 283 | GENE | — | 44 | 63 |

| **Table S5g Characteristics description of studies included in meta-analysis of herpes simplex virus (HSV) prevalence** | | | | | | | |  |  |
| --- | --- | --- | --- | --- | --- | --- | --- | --- | --- |
| **First Author &** | **Study** | **Study Location** |  | **Subgroup** | **Sample** | **Presumptive** | **Confirmatory** | **No. of** | **No. of people** |
| **Published Year** | **Period** | **City/county** | **Province** | **Population** | **Size** | **test(s)** | **test(s)*** | **infection** | **tested** |
| HSV |  |  |  |  |  |  |  |  |  |
| Chen XS, 2006[^36^](#_ENREF_36) | 2000 | Tongling | Anhui | Truck drivers | 550 | — | ELISA | 24 | 545 |
| Sun BW, 1997[^349^](#_ENREF_349) | 1996-1997 | Jiangyan | Jiangsu | Construction workers | 5892 | — | — | 15 | 5892 |
| Ma YL, 2010[^352^](#_ENREF_352) | 2006 | Pingdu | Shandong | Unspecified job nature | 396 | — | — | 3 | 396 |
| Qiu HP, 2007[^374^](#_ENREF_374) | 2005 | Quzhou | Zhejiang | Unspecified job nature | 219 | ELISA | WB | 58 | 219 |
| Tang SK, 2008[^375^](#_ENREF_375) | 2005-2006 | Guangzhou | Guangdong | Unspecified job nature | 300 | ELISA | ELISA | 44 | 300 |
| Zhong YX, 2008[^376^](#_ENREF_376) | — | Shanghai | Shanghai | Unspecified job nature | 398 | ELISA | — | 31 | 398 |
| Zhu JQ, 2012[^146^](#_ENREF_146) | 2010 | Jinzhou | Zhejiang | Unspecified job nature | 403 | ELISA | — | 1 | 403 |
| He N, 2009[^377^](#_ENREF_377) | — | Shanghai | Shanghai | Unspecified job nature | 895 | ELISA | — | 49 | 895 |
| Gao LL, 2007[^378^](#_ENREF_378) | 2007 | — | Yunnan | FSWs | 270 | YES | — | 0 | 97 |
| Yang Y, 2011a[^248^](#_ENREF_248) | 2008 | Minhang | Shanghai | FSWs | 411 | ELISA | — | 169 | 382 |
| Yang Y, 2011b[^248^](#_ENREF_248) | 2009 | Minhang | Shanghai | FSWs | 411 | ELISA | — | 188 | 382 |

| **Table S5h Characteristics description of studies included in meta-analysis of hepatitis B virus (HBV) prevalence** | | | | | | | |  |  |
| --- | --- | --- | --- | --- | --- | --- | --- | --- | --- |
| **First Author &** | **Study** | **Study Location** |  | **Subgroup** | **Sample** | **Presumptive** | **Confirmatory** | **No. of** | **No. of people** |
| **Published Year** | **Period** | **City/county** | **Province** | **Population** | **Size** | **test(s)** | **test(s)*** | **infection** | **tested** |
| HBV |  |  |  |  |  |  |  |  |  |
| Su SL, 2011[^15^](#_ENREF_15) | 2008 | Shandong | Shandong | Migrant women | 7102 | Yes | — | 950 | 7102 |
| Sun T, 2008[^379^](#_ENREF_379) | 2008 | Daiyue, Tai'an | Shandong | Migrant women | 160 | ELISA | — | 7 | 160 |
| Zhang SY, 2009[^31^](#_ENREF_31) | 2007 | Ju county | Shandong | Migrant women | 742 | ELISA | — | 66 | 742 |
| Nong LP, 2005[^46^](#_ENREF_46) | — | — | — | Truck drivers | 368 | ELISA | — | 45 | 368 |
| He L, 2012[^57^](#_ENREF_57) | 2010 | Shenzhen | Guangdong | Construction workers | 101 | ELISA | — | 14 | 101 |
| Sheng MY, 2010[^61^](#_ENREF_61) | 2008 | Haining, Jiaxing | Zhejiang | Construction workers | 231 | ELISA | — | 28 | 231 |
| Wang CH, 2012[^63^](#_ENREF_63) | 2011 | Huanggang | Hubei | Construction workers | 332 | ELISA | — | 42 | 301 |
| Xu XP, 2010[^65^](#_ENREF_65) | 2007 | Jiande | Zhejiang | Construction workers | 529 | — | — | 75 | 529 |
| Huang RR, 2008[^69^](#_ENREF_69) | — | Jinshan District | Shanghai | Factory workers | 310 | ELISA | — | 12 | 310 |
| Fan YD, 2007a[^380^](#_ENREF_380) | 2006 | Gaotai | Gansu | Unspecified job nature | 2187 | ELISA | — | 49 | 688 |
| Fan YD, 2007b[^380^](#_ENREF_380) | 2005 | Gaotai | Gansu | Unspecified job nature | 2187 | ELISA | — | 41 | 584 |
| Fan YD, 2007c[^380^](#_ENREF_380) | 2004 | Gaotai | Gansu | Unspecified job nature | 2187 | ELISA | — | 74 | 915 |
| Fei, P, 2010a[^381^](#_ENREF_381) | 2001 | Zhangzhou | Fujian | Unspecified job nature | 2917 | ELISA | — | 263 | 2917 |
| Fei, P, 2010b[^381^](#_ENREF_381) | 2002 | Zhangzhou | Fujian | Unspecified job nature | 5542 | ELISA | — | 462 | 5542 |
| Fei, P, 2010c[^381^](#_ENREF_381) | 2003 | Zhangzhou | Fujian | Unspecified job nature | 5283 | ELISA | — | 389 | 5283 |
| Fei, P, 2010d[^381^](#_ENREF_381) | 2004 | Zhangzhou | Fujian | Unspecified job nature | 5490 | ELISA | — | 387 | 5490 |
| Fei, P, 2010e[^381^](#_ENREF_381) | 2005 | Zhangzhou | Fujian | Unspecified job nature | 5553 | ELISA | — | 387 | 5553 |
| Fei, P, 2010f[^381^](#_ENREF_381) | 2006 | Zhangzhou | Fujian | Unspecified job nature | 6453 | ELISA | — | 319 | 6453 |
| Fei, P, 2010g[^381^](#_ENREF_381) | 2007 | Zhangzhou | Fujian | Unspecified job nature | 3094 | ELISA | — | 142 | 3094 |
| Fei, P, 2010h[^381^](#_ENREF_381) | 2008 | Zhangzhou | Fujian | Unspecified job nature | 2557 | ELISA | — | 102 | 2557 |
| Fei, P, 2010i[^381^](#_ENREF_381) | 2009 | Zhangzhou | Fujian | Unspecified job nature | 1150 | ELISA | — | 41 | 1150 |
| Gong T, 2007[^382^](#_ENREF_382) | 2004-2005 | Chongqing | Chongqing | Unspecified job nature | 1195 | ELISA | — | 103 | 1195 |
| Gu MD, 2009[^80^](#_ENREF_80) | 2007 | Pingjiang | Hunan | Unspecified job nature | 576 | — | — | 85 | 576 |
| Gu, ZD, 2006[^383^](#_ENREF_383) | 2004 | Dongguan | Guangdong | Unspecified job nature | 2290 | ELISA | — | 296 | 2290 |
| **Table S5h Characteristics description of studies included in meta-analysis of hepatitis B virus (HBV) prevalence (Cont’d)** | | | | | | | |  |  |
| **First Author &** | **Study** | **Study Location** |  | **Subgroup** | **Sample** | **Presumptive** | **Confirmatory** | **No. of** | **No. of people** |
| **Published Year** | **Period** | **City/county** | **Province** | **Population** | **Size** | **test(s)** | **test(s)*** | **infection** | **tested** |
| HBV (Cont’d) |  |  |  |  |  |  |  |  |  |
| Hou JH, 2007[^384^](#_ENREF_384) | 2006 | Shenzhen | Guangdong | Unspecified job nature | 2274 | — | — | 624 | 2274 |
| Li J, 2011[^385^](#_ENREF_385) | 2006-2010 | Shangqiu | shandong | Unspecified job nature | 2462 | ELISA | — | 166 | 2462 |
| Li L, 2005[^386^](#_ENREF_386) | 2005 | Wuhan | Hubei | Unspecified job nature | 1565 | — | — | 224 | 1565 |
| Li YN, 2006a[^387^](#_ENREF_387) | 2001 | Shenzhen | Guangdong | Unspecified job nature | 1667 | — | HBV Casset | 62 | 1667 |
| Li YN, 2006b[^387^](#_ENREF_387) | 2002 | Shenzhen | Guangdong | Unspecified job nature | 1815 | — | HBV Casset | 75 | 1815 |
| Li YN, 2006c[^387^](#_ENREF_387) | 2003 | Shenzhen | Guangdong | Unspecified job nature | 1660 | — | HBV Casset | 42 | 1660 |
| Li YN, 2006d[^387^](#_ENREF_387) | 2004 | Shenzhen | Guangdong | Unspecified job nature | 1971 | — | HBV Casset | 42 | 1971 |
| Mao LH, 2011a[^388^](#_ENREF_388) | 2003 | Jiangxi | Jiangxi | Unspecified job nature | 4567 | ELISA | — | 416 | 4567 |
| Mao LH, 2011b[^388^](#_ENREF_388) | 2004 | Jiangxi | Jiangxi | Unspecified job nature | 4185 | ELISA | — | 349 | 4185 |
| Mao LH, 2011c[^388^](#_ENREF_388) | 2005 | Jiangxi | Jiangxi | Unspecified job nature | 9064 | ELISA | — | 579 | 9064 |
| Meng, QY, 2009[^389^](#_ENREF_389) | 2008 | Beijing | Beijing | Unspecified job nature | 61285 | ELISA | — | 2153 | 61285 |
| Qiu JQ, 2008[^116^](#_ENREF_116) | 2004-2006 | Kaihua | Zhejiang | Unspecified job nature | 1557 | — | HBV Casset | 212 | 1557 |
| Qiu JQ, 2011[^117^](#_ENREF_117) | 2010 | Kaihua | Zhejiang | Unspecified job nature | 873 | — | — | 116 | 873 |
| Tan XW, 2006[^390^](#_ENREF_390) | 2003 | Shenzhen | Guangdong | Unspecified job nature | 112113 | ELISA | — | 16527 | 112113 |
| Wang LJ, 2006[^125^](#_ENREF_125) | — | Congwen | Beijing | Unspecified job nature | 380 | — | — | 44 | 380 |
| Wang WD, 2007a[^391^](#_ENREF_391) | 2002 | Kaifeng | Henan | Unspecified job nature | 896 | ELISA | — | 35 | 896 |
| Wang WD, 2007b[^391^](#_ENREF_391) | 2003 | Kaifeng | Henan | Unspecified job nature | 1203 | ELISA | — | 38 | 1203 |
| Wang WD, 2007c[^391^](#_ENREF_391) | 2004 | Kaifeng | Henan | Unspecified job nature | 928 | ELISA | — | 30 | 928 |
| Wang WD, 2007d[^391^](#_ENREF_391) | 2005 | Kaifeng | Henan | Unspecified job nature | 1321 | ELISA | — | 37 | 1321 |
| Wen HH, 2006[^392^](#_ENREF_392) | 2002-2003 | Shenzhen | Guangdong | Unspecified job nature | 1321 | ELISA | — | 167 | 1321 |
| Yang RX, 2007[^393^](#_ENREF_393) | 2006 | Shenzhen | Guangdong | Unspecified job nature | 300786 | ELISA | — | 38801 | 300786 |
| Yang XF, 2008[^394^](#_ENREF_394) | 2006 | Yinzhou, Ningbo | Zhejiang | Unspecified job nature | 19403 | ELISA | — | 1404 | 14687 |
| Yu GL, 2006[^395^](#_ENREF_395) | 2004-2005 | Guangzhou | Guangdong | Unspecified job nature | 1137 | ELISA | — | 319 | 1137 |
| **Table S5h Characteristics description of studies included in meta-analysis of hepatitis B virus (HBV) prevalence (Cont’d)** | | | | | | | |  |  |
| **First Author &** | **Study** | **Study Location** |  | **Subgroup** | **Sample** | **Presumptive** | **Confirmatory** | **No. of** | **No. of people** |
| **Published Year** | **Period** | **City/county** | **Province** | **Population** | **Size** | **test(s)** | **test(s)*** | **infection** | **tested** |
| HBV (Cont’d) |  |  |  |  |  |  |  |  |  |
| Zang DA, 2008[^142^](#_ENREF_142) | 2006-2007 | Sheyang | Jiangsu | Unspecified job nature | 3554 | — | — | 168 | 3554 |
| Zhang YB, 2012[^396^](#_ENREF_396) | — | Shenzhen | Guangdong | Unspecified job nature | 1873 | — | — | 132 | 1873 |
| Zhang, YJ, 2009[^397^](#_ENREF_397) | 2005-2007 | Xianju | Zhejiang | Unspecified job nature | 4710 | ELISA | — | 747 | 4710 |
| Zhao SK, 2005[^398^](#_ENREF_398) | 2003 | Daxing | Beijing | Unspecified job nature | 11817 | ELISA | — | 1403 | 11817 |
| Zheng SM, 2007[^399^](#_ENREF_399) | 2004-2005 | Dongwan, Guangzhou | Guangdong | Unspecified job nature | 962 | ELISA | — | 335 | 962 |
| Zhong YW, 2003[^400^](#_ENREF_400) | — | Shenzhen | Guangdong | Unspecified job nature | 4447 | ELISA | — | 556 | 4447 |
| Zhou JH, 2009[^401^](#_ENREF_401) | 2006 | Xiuzhou | Zhejiang | Unspecified job nature | 326 | ELISA | — | 23 | 326 |
| Zhu QX, 2007[^402^](#_ENREF_402) | — | Xianju | Zhejiang | Unspecified job nature | 1117 | ELISA | — | 191 | 1117 |
| Zhu QX, 2009[^403^](#_ENREF_403) | 2005-2007 | Xianju | Zhejiang | Unspecified job nature | 5658 | ELISA | — | 897 | 5658 |
| Jiang PF, 2011[^404^](#_ENREF_404) | 2010 | Shenzhen | Guangdong | Pregnant women | 1031 | ELISA | — | 76 | 1031 |
| Li QC, 2000a[^405^](#_ENREF_405) | 1993-1995 | Guangxi | Guangxi | Pregnant women | 1741 | EIA | — | 148 | 1741 |
| Li QC, 2000b[^405^](#_ENREF_405) | 1995-1996 | Guangxi | Guangxi | Pregnant women | 1743 | EIA | — | 203 | 1743 |
| Li SH, 2012a[^152^](#_ENREF_152) | 2007 | Beijing | Beijing | Pregnant women | 26819 | CLEIA | CLEIA | 85 | 6730 |
| Li SH, 2012b[^152^](#_ENREF_152) | 2008 | Beijing | Beijing | Pregnant women | 26819 | CLEIA | CLEIA | 96 | 6869 |
| Li SH, 2012c[^152^](#_ENREF_152) | 2009 | Beijing | Beijing | Pregnant women | 26819 | CLEIA | CLEIA | 132 | 7076 |
| Li SH, 2012d[^152^](#_ENREF_152) | 2010 | Beijing | Beijing | Pregnant women | 26819 | CLEIA | CLEIA | 144 | 6144 |
| Huang F, 2005[^307^](#_ENREF_307) | — | Shenzhen | Guangdong | DUs | 86 | — | — | 11 | 86 |
| Tao XY, 2004[^328^](#_ENREF_328) | 2003 | Shenzhen | Guangdong | DUs | 198 | — | — | 5 | 49 |

| **Table S5i Characteristics description of studies included in meta-analysis of hepatitis C virus (HCV) prevalence** | | | | | | | |  |  |
| --- | --- | --- | --- | --- | --- | --- | --- | --- | --- |
| **First Author &** | **Study** | **Study Location** |  | **Subgroup** | **Sample** | **Presumptive** | **Confirmatory** | **No. of** | **No. of people** |
| **Published Year** | **Period** | **City/county** | **Province** | **Population** | **Size** | **test(s)** | **test(s)*** | **infection** | **tested** |
| HCV |  |  |  |  |  |  |  |  |  |
| Su SL, 2011[^15^](#_ENREF_15) | 2008 | Shandong | Shandong | Migrant women | 7102 | YES | — | 206 | 7102 |
| Wang Q, 2009[^19^](#_ENREF_19) | 2009 | Qidong | Hunan | Migrant women | 2709 | — | — | 1 | 95 |
| Zhang SY, 2009[^31^](#_ENREF_31) | 2007 | Ju county | Shandong | Migrant women | 742 | ELISA | — | 10 | 742 |
| Guo WQ, 2010[^38^](#_ENREF_38) | 2006 | lanzhou | Gansu | Truck drivers | 400 | — | — | 4 | 400 |
| Liao MZ, 2010[^43^](#_ENREF_43) | 2009 | Jinan | Shandong | Truck drivers | 425 | ELISA-1 | ELISA-2 | 3 | 425 |
| Yi JF, 2010[^52^](#_ENREF_52) | 2009 | yichun | Jiangsu | Truck drivers | 400 | — | — | 5 | 400 |
| He B, 2012[^56^](#_ENREF_56) | 2011 | Pinxiang | Guangxi | Construction workers | 405 | — | ELISA | 4 | 405 |
| He L, 2012[^57^](#_ENREF_57) | 2010 | shenzhen | Guangdong | Construction workers | 101 | ELISA | — | 1 | 101 |
| He QH, 2010[^84^](#_ENREF_84) | 2009 | fushan | Guangdong | Construction workers | 160 | — | ELISA | 0 | 160 |
| Hou XY, 2011[^406^](#_ENREF_406) | 2010 | Huhehaote | Inner Mongolia | Construction workers | 411 | ELISA | ELISA | 2 | 411 |
| Hu XQ, 2011[^58^](#_ENREF_58) | 2010 | Baoji | Shaanxi | Construction workers | 400 | ELISA | ELISA | 7 | 400 |
| Liu HX, 2011[^59^](#_ENREF_59) | 2010 | Changping | Beijing | Construction workers | 449 | — | — | 3 | 449 |
| Mou XL, 2012[^60^](#_ENREF_60) | 2010 | Wuxi | Jiangsu | Construction workers | 1703 | ELISA-1 | ELISA-2 | 0 | 481 |
| Wang CH, 2012a[^63^](#_ENREF_63) | 2011 | huanggang | Hubei | Construction workers | 332 | ELISA | — | 3 | 301 |
| Xia DY, 2011a[^64^](#_ENREF_64) | 2009 | Beijing | Beijing | Construction workers | 3161 | ELISA-1 | ELISA-2 | 25 | 3161 |
| Zhang P, 2011[^66^](#_ENREF_66) | — | Pinxiang | Jilin | Construction workers | 400 | — | — | 2 | 400 |
| Xia DY, 2011b[^64^](#_ENREF_64) | 2009 | Kuancheng, Changchun | Beijing | Factory workers | 59 | ELISA-1 | ELISA-2 | 0 | 59 |
| He B, 2012[^56^](#_ENREF_56) | 2011 | Jinshan District | Guangxi | Factory workers | 403 | — | ELISA | 1 | 403 |
| Huang RR, 2008[^69^](#_ENREF_69) | — | Wuxi | Shanghai | Factory workers | 310 | ELISA | — | 0 | 310 |
| Mou XL, 2012b[^60^](#_ENREF_60) | 2010 | Shanghai | Jiangsu | Factory workers | 1703 | ELISA-1 | ELISA-2 | 0 | 399 |
| Wei W, 2012[^71^](#_ENREF_71) | 2012 | Beijing | Shanghai | Factory workers | 404 | — | — | 0 | 404 |
| Mou XL, 2012c[^60^](#_ENREF_60) | 2010 | Wuxi | Jiangsu | Restaurant attendants | 1703 | ELISA-1 | ELISA-2 | 0 | 400 |
| Mou XL, 2012d[^60^](#_ENREF_60) | 2010 | Lanzhou | Jiangsu | Unspecified job nature | 1703 | ELISA-1 | ELISA-2 | 0 | 408 |
| **Table S5i Characteristics description of studies included in meta-analysis of hepatitis C virus (HCV) prevalence (Cont’d)** | | | | | | | |  |  |
| **First Author &** | **Study** | **Study Location** |  | **Subgroup** | **Sample** | **Presumptive** | **Confirmatory** | **No. of** | **No. of people** |
| **Published Year** | **Period** | **City/county** | **Province** | **Population** | **Size** | **test(s)** | **test(s)*** | **infection** | **tested** |
| HCV (Cont’d) |  |  |  |  |  |  |  |  |  |
| Xia DY, 2011c[^64^](#_ENREF_64) | 2009 | Foshan | Beijing | Unspecified job nature | 967 | ELISA-1 | ELISA-2 | 0 | 967 |
| Chen SH, 2012[^76^](#_ENREF_76) | 2010 | Jinjiang | Guangdong | Unspecified job nature | 1611 | ELISA | — | 8 | 1611 |
| Cui DZ, 2012[^77^](#_ENREF_77) | 2012 | Jinshan District | Gansu | Unspecified job nature | 400 | ELISA | — | 3 | 400 |
| Hong PK, 2010[^86^](#_ENREF_86) | 2010 | Jinshan District | Fujian | Unspecified job nature | 400 | — | — | 1 | 400 |
| Jiang CH, 2012a[^92^](#_ENREF_92) | 2009 | Jinshan District | Shanghai | Unspecified job nature | 400 | ELISA | — | 0 | 400 |
| Jiang CH, 2012b[^92^](#_ENREF_92) | 2010 | Jiangyin | Shanghai | Unspecified job nature | 405 | ELISA | — | 1 | 405 |
| Jiang CH, 2012c[^92^](#_ENREF_92) | 2011 | Yangquan | Shanghai | Unspecified job nature | 400 | ELISA | — | 2 | 400 |
| Jiang JH, 2012[^93^](#_ENREF_93) | 2012 | Yangquan | Jiangsu | Unspecified job nature | 400 | ELISA-1 | ELISA-2 | 1 | 400 |
| Jing LH, 2010[^95^](#_ENREF_95) | 2009 | Hefei | Shanxi | Unspecified job nature | 409 | ELISA | ELISA | 1 | 409 |
| Kang YF, 2011[^96^](#_ENREF_96) | 2008-2010 | Fujian | Shanxi | Unspecified job nature | 813 | ELISA | ELISA | 3 | 813 |
| Li XJ, 2011[^193^](#_ENREF_193) | 2009 | Changle,Jinjiang,Meilie | Anhui | Unspecified job nature | 1657 | — | — | 5 | 1657 |
| Lin X, 2011[^103^](#_ENREF_103) | 2010 | Xuanhan | Fujian | Unspecified job nature | 1705 | ELISA-1 | ELISA-2 | 2 | 1705 |
| Lin YT,2012[^104^](#_ENREF_104) | 2010 | Jiangyin | Fujian | Unspecified job nature | 4167 | ELISA | ELISA | 4 | 4098 |
| Liu SZ, 2012[^106^](#_ENREF_106) | 2010 | Wuxi | Sichuan | Unspecified job nature | 400 | ELISA-1 | ELISA-2 | 0 | 400 |
| Lu JJ, 2011[^107^](#_ENREF_107) | 2011 | Wuxi | Jiangsu | Unspecified job nature | 400 | ELISA-1 | ELISA-2 | 2 | 400 |
| Mou XL, 2012[^60^](#_ENREF_60) | 2010 | Wuxi | Jiangsu | Unspecified job nature | 1703 | ELISA-1 | ELISA-2 | 0 | 1688 |
| Mou XL, 2011[^112^](#_ENREF_112) | 2010 | Xinghua | Jiangsu | Unspecified job nature | 400 | ELISA-1 | ELISA-2 | 1 | 400 |
| Pan XH, 2013[^114^](#_ENREF_114) | 2010 | Ningbo | Zhejiang | Unspecified job nature | 17377 | ELISA | WB | 70 | 17377 |
| Qian ZY, 2011[^115^](#_ENREF_115) | 2010 | Beijing | Jiangsu | Unspecified job nature | 511 | — | — | 2 | 511 |
| Shi XJ, 2011[^119^](#_ENREF_119) | 2010 | Beijing | Zhejiang | Unspecified job nature | 800 | ELISA-1 | ELISA-2 | 4 | 793 |
| Xia DY, 2011[^64^](#_ENREF_64) | 2009 | Kunshan | Beijing | Unspecified job nature | 4187 | ELISA-1 | ELISA-2 | 25 | 4187 |
| Xue LJ, 2011[^132^](#_ENREF_132) | 2010 | Sanming | Jiangsu | Unspecified job nature | 400 | — | — | 1 | 400 |
| Yang MH, 2011[^135^](#_ENREF_135) | 2010 | Beijing | Fujian | Unspecified job nature | 402 | ELISA-1 | ELISA-2 | 1 | 402 |
| **Table S5i Characteristics description of studies included in meta-analysis of hepatitis C virus (HCV) prevalence (Cont’d)** | | | | | | | |  |  |
| **First Author &** | **Study** | **Study Location** |  | **Subgroup** | **Sample** | **Presumptive** | **Confirmatory** | **No. of** | **No. of people** |
| **Published Year** | **Period** | **City/county** | **Province** | **Population** | **Size** | **test(s)** | **test(s)*** | **infection** | **tested** |
| HCV (Cont’d) |  |  |  |  |  |  |  |  |  |
| Zhu YY, 2012[^147^](#_ENREF_147) | 2009 | Nanjing | Beijing | Unspecified job nature | 4187 | ELISA | — | 25 | 4187 |
| Li SH, 2012a[^152^](#_ENREF_152) | 2007 | Beijing | Beijing | Pregnant women | 26819 | YES | — | 2 | 6730 |
| Li SH, 2012b[^152^](#_ENREF_152) | 2008 | Beijing | Beijing | Pregnant women | 26819 | YES | — | 1 | 6869 |
| Li SH, 2012c[^152^](#_ENREF_152) | 2009 | Beijing | Beijing | Pregnant women | 26819 | YES | — | 2 | 7076 |
| Li SH, 2012d[^152^](#_ENREF_152) | 2010 | Beijing | Beijing | Pregnant women | 26819 | YES | — | 3 | 6144 |
| Nong LP, 2011[^154^](#_ENREF_154) | 2009-2010 | Pingxiang | Guangxi | Pregnant women | 800 | ELISA | — | 1 | 117 |
| Chen ZB, 2011[^166^](#_ENREF_166) | 2007 | Lianzhou | Guangdong | FSWs | 200 | ELISA-1 | ELISA-2 | 0 | 173 |
| Guo H, 2012[^176^](#_ENREF_176) | 2010-2011 | Longnan | Gansu | FSWs | 820 | ELISA | — | 0 | 104 |
| He B, 2012[^407^](#_ENREF_407) | 2009 | Pinxiang | Guangxi | FSWs | 206 | YES | — | 0 | 95 |
| Liao CQ, 2011[^196^](#_ENREF_196) | — | Hongkou | Shanghai | FSWs | 401 | — | — | 1 | 401 |
| Ling Z, 2012[^408^](#_ENREF_408) | — | — | Zhejiang | FSWs | 275 | — | — | 0 | 139 |
| Qin CZ, 2012[^214^](#_ENREF_214) | 2010-2011 | Jiangyan | Jiangsu | FSWs | 817 | ELISA-1 | ELISA-2 | 0 | 347 |
| Lan GH, 2009[^276^](#_ENREF_276) | 2008 | Guangxi | Guangxi | MSM | 179 | ELISA | — | 0 | 179 |
| Liang L, 2009[^277^](#_ENREF_277) | 2008 | Shijiazhuang | Hebei | MSM | 450 | ELISA | RIBA | 1 | 200 |
| Liu PL, 2010[^278^](#_ENREF_278) | 2008 | Wuhan | Hubei | MSM | 456 | — | — | 4 | 237 |
| Ma XY, 2007[^409^](#_ENREF_409) | 2004-2006 | Taiyuan | Beijing | MSM | 540 | ELISA | — | 27 | 540 |
| Mei L, 2009[^280^](#_ENREF_280) | 2008 | Beijing | Shanxi | MSM | 273 | ELISA | YES | 0 | 184 |
| Fu DL, 2009[^306^](#_ENREF_306) | 2009 | Nanchan | Hubei | DUs | 400 | — | — | 180 | 400 |
| Huang F, 2005[^307^](#_ENREF_307) | — | Shenzhen | Guangdong | DUs | 86 | — | — | 42 | 86 |
| Li DL, 2012[^311^](#_ENREF_311) | 2007 | Changzhou | Beijing | DUs | 128 | ELISA | — | 81 | 128 |
| Liu SF, 2008[^319^](#_ENREF_319) | 2005-2007 | Taishan | Jiangsu | DUs | 855 | — | — | 158 | 328 |
| Luo WQ, 2012[^323^](#_ENREF_323) | 2010-2012 | Jiangsu | Guangdong | DUs | 1386 | ELISA | — | 91 | 206 |
| Shi P, 2009[^410^](#_ENREF_410) | 2005-2008 | Shenzhen | Jiangsu | DUs | 2811 | ELISA | YES | 226 | 678 |
| Tao XY, 2004[^328^](#_ENREF_328) | 2003 | Jinhua | Guangdong | DUs | 198 | — | — | 31 | 49 |
| **Table S5i Characteristics description of studies included in meta-analysis of hepatitis C virus (HCV) prevalence (Cont’d)** | | | | | | | |  |  |
| **First Author &** | **Study** | **Study Location** |  | **Subgroup** | **Sample** | **Presumptive** | **Confirmatory** | **No. of** | **No. of people** |
| **Published Year** | **Period** | **City/county** | **Province** | **Population** | **Size** | **test(s)** | **test(s)*** | **infection** | **tested** |
| HCV (Cont’d) |  |  |  |  |  |  |  |  |  |
| Wang SJ, 2012[^411^](#_ENREF_411) | 2009-2010 | Beijing | Zhejiang | DUs | 1040 | ELISA | — | 398 | 757 |

ELISA: Enzyme-linked Immuno Sorbent Assay; WB: Western Blot (HIV testing); PA: Gelatin particle agglutination method; LCR: Ligase Chain reaction; MEIA: Microparticle Enzyme Immune Assay; HC: Hybrid Capture; RAT: Rapid Antigen Test; CLEIA: Chemiluminescence Immunoassay; ABDSTT: Ab-bott Determine Syphilis TP test; PPAT: Passive particle agglutination test; TPHA: Treponema pallidum hemagglutination assay; TPPA: Treponema pallidum particle agglutination; TRUST: Toluidine Red Unheated Serum Test ;

**Reference:**

**Table S6** Time trend test for low-risk groups included in meta-analysis

| **Disease** | ***N*** | ***t*** | ***p*** |
| --- | --- | --- | --- |
| HIV | 201 | -0.710 | 0.479 |
| Syphilis | 110 | 0.647 | 0.519 |
| Gonorrhea | 13 | 0.512 | 0.620 |
| Chlamydia | 8 | -0.050 | 0.962 |
| Genital warts | 6 | 1.255 | 0.278 |
| HSV | 8 | 0.086 | 0.935 |
| HPV | 3 | 0.656 | 0.631 |
| HBV | 63 | -0.146 | 0.884 |
| HCV | 52 | 0.224 | 0.823 |

**Table S7 Prevalence and odds ratios of sexually transmitted and viral hepatitis infections among rural-to-urban migrants, migrant pregnant women and high-risk groups with a migratory background comparing with national prevalences or systematic review studies among background population**

| **Subgroups** | **No. of estimates (No. of studies)** | **Pooled Prevalence (%) and 95% CI** | **Heterogeneity** | | **Publication bias (*p* value)** | **Background prevalence (%) and 95% CI†** | **Odds Ratio (95% CI)** |
| --- | --- | --- | --- | --- | --- | --- | --- |
|  |  |  | ***I^2^*** | ***p*** |  |  |  |
| ***HIV*** | 401 (345) |  |  |  |  |  |  |
| **Rural-to-urban migrants in various occupations** | 188 (149) | 0.23 (0.20-0.27) | 72.58 | <0.01‥ | 0.01^＊＊^ | 0.06 (0.05-0.07)[^1^](#_ENREF_1) | 4.0 (3.1-5.2)* |
| Migrant women through marriage | 37 (35) | 0.46 (0.38-0.57) | 75.90 | <0.01‥ | 0.30 | 0.06 (0.05-0.07)[^1^](#_ENREF_1) | 8.0 (6.1-10.5)* |
| Construction workers | 13(13) | 0.17 (0.08-0.33) | 0.00 | 0.85 | <0.01^＊＊^ | 0.06 (0.05-0.07)[^1^](#_ENREF_1) | 2.8 (1.6-4.9)* |
| Truck drivers | 23 (19) | 0.12 (0.08-0.19) | 0.00 | 0.91 | <0.01^＊＊^ | 0.06 (0.05-0.07)[^1^](#_ENREF_1) | 2.0 (1.4-2.9)* |
| Miners | 1 (1) | 0.55 (0.08-3.79) | 0.00 | 1.00 | **--** | **--** | **--** |
| Factory workers | 8 (8) | 0.20 (0.09-0.44) | 0.00 | 0.91 | 0.05 | 0.06 (0.05-0.07)[^1^](#_ENREF_1) | 3.3 (1.9-5.9)* |
| Restaurant attendants | 2 (2) | 0.26 (0.09-0.74) | 0.00 | 0.58 | **--** | 0.06 (0.05-0.07)[^1^](#_ENREF_1) | 4.3 (2.2-8.5)* |
| Migrants with unspecified job nature | 104 (86) | 0.16 (0.12-0.21) | 71.84 | <0.01‥ | 0.09 | 0.06 (0.05-0.07)[^1^](#_ENREF_1) | 2.7 (2.0-3.8)* |
| **Migrant pregnant women** | 12 (9) | 0.10 (0.02-0.49) | 95.26 | <0.01‥ | 0.58 | 0.01 (0.01-0.02)[^2^](#_ENREF_2) | 7.7 (3.4-17.4)* |
| **High-risk groups with a migratory background** |  |  |  |  |  |  |  |
| FSWs | 119 (110) | 0.31 (0.19-0.48) | 84.41 | <0.01‥ | <0.01^＊＊^ | 0.30 (0.28-0.32)[^3^](#_ENREF_3) | 1.0 (0.7-1.5) |
| MSM | 29 (29) | 5.88 (4.48-7.68) | 87.21 | <0.01‥ | 0.07 | 6.30 (6.05-6.55)[^3^](#_ENREF_3) | 0.9 (0.7-1.2) |
| DUs | 52 (48) | 4.45 (3.43-5.76) | 92.51 | <0.01‥ | 0.09 | 6.00 (4.70-7.70)[^4^](#_ENREF_4) | 0.7 (0.5-1.0) |
| ***Syphilis*** | **166 (138)** |  |  |  |  |  |  |
| **Rural-to-urban migrants in various occupations** | 100 (80) | 0.69 (0.57-0.84) | 83.65 | <0.01‥ | 0.02^＊^ | 0.37 (0.20-0.65)[^5^](#_ENREF_5) | 1.9 (1.1-3.0)* |
| Migrant women through marriage | 9 (9) | 0.91 (0.60-1.39) | 90.28 | <0.01‥ | 0.11 | 0.37 (0.20-0.65)[^5^](#_ENREF_5) | 2.5 (1.4-4.4)* |
| Construction workers | 12 (12) | 0.88 (0.44-1.74) | 86.91 | <0.01‥ | 0.34 | 0.37 (0.20-0.65)[^5^](#_ENREF_5) | 2.4 (1.2-4.7)* |
| Truck drivers | 8 (8) | 0.72 (0.27-1.93) | 81.88 | <0.01‥ | 0.22 | 0.37 (0.20-0.65)[^5^](#_ENREF_5) | 1.4 (0.7-2.9) |
| Miners | **--** | **--** | **--** | **--** | **--** | **--** | **--** |
| Factory workers | 7 (7) | 0.52 (0.19-1.42) | 67.23 | 0.01‥ | 0.29 | 0.37 (0.20-0.65)[^5^](#_ENREF_5) | 1.4 (0.6-3.1) |
| Restaurant attendants | 2 (2) | 0.52 (0.12-2.29) | 37.18 | 0.21 | **--** | 0.37 (0.20-0.65)[^5^](#_ENREF_5) | 1.4 (0.6-3.5) |
| Migrants with unspecified job nature | 62 (54) | 0.62 (0.49-0.79) | 80.60 | <0.01‥ | 0.05 | 0.37 (0.20-0.65)[^5^](#_ENREF_5) | 1.7 (1.0-2.8)* |
| **Migrant pregnant women** | 10 (7) | 0.29 (0.22-0.40) | 78.57 | <0.01‥ | 0.65 | 0.45 (0.29-0.60)[^6^](#_ENREF_6) | 0.6 (0.4-1.0) |
| **High-risk groups with a migratory background** |  |  |  |  |  |  |  |
| FSWs | 20 (18) | 4.19 (3.17-5.52) | 78.38 | <0.01‥ | 0.09 | 5.00 (4.50-5.50)[^7^](#_ENREF_7) | 0.8 (0.6~1.1) |
| MSM | 17 (17) | 12.67 (10.44-15.30) | 81.43 | <0.01‥ | 0.46 | 13.45 (11.78-15.23) | 0.9 (0.7-1.2) |
| DUs | 19 (16) | 9.16 (7.12-11.73) | 88.41 | <0.01‥ | 0.17 | 6.81 (5.01-11.27)[^6^](#_ENREF_6) | 1.4 (0.9-2.0) |

| **Table S7 Prevalence and odds ratios of sexually transmitted and viral hepatitis infections among rural-to-urban migrants, migrant pregnant women and high-risk groups with a migratory background compared with national prevalence or systematic review studies among background population (Cont’d)** | | | | | | | |
| --- | --- | --- | --- | --- | --- | --- | --- |
| **Subgroups** | **No. of estimates (No. of studies)** | **Pooled Prevalence (%) and 95% CI** | **Heterogeneity** | | **Publication bias (*p* value)** | **Background prevalence (%) and 95% CI†** | **Odds Ratio (95% CI)** |
|  |  |  | ***I^2^*** | ***p*** |  |  |  |
| ***Gonorrhea*** | **15 (14)** |  |  |  |  |  |  |
| **Rural-to-urban migrants in various occupations** | 13(12) | 2.18 (1.30-3.64) | 94.56 | <0.01‥ | 0.71 | 0.16 (0.06-0.39) | 13.6 (5.8-32.1)* |
| Migrant women through marriage | 2 (2) | 2.19 (0.40-11.20) | 50.60 | 0.15 | -- | 0.16 (0.06-0.39) | 13.7 (4.8-38.9)* |
| Construction workers | 1 (1) | 3.12 (2.71-3.60) | 0.00 | 1.00 | -- | -- | -- |
| Truck drivers | 1 (1) | 8.11 (6.07-10.76) | 0.00 | 1.00 | -- | -- | -- |
| Miners | 1 (1) | 0.55 (0.08-3.79) | 0.00 | 1.00 | -- | -- | -- |
| Factory workers | -- | -- | -- | -- | -- | -- | -- |
| Restaurant attendants | -- | -- | -- | -- | -- | -- | -- |
| Migrants with unspecified job nature | 8 (7) | 1.60 (0.47-5.31) | 96.02 | <0.01‥ | 0.80 | 0.16 (0.06-0.39) | 10.0 (3.5-28.2)* |
| **Migrant pregnant women** | **--** | **--** | **--** | **--** | **--** | **--** | **--** |
| **High-risk groups with a migratory background** | **--** | **--** | **--** | **--** | **--** | **--** | **--** |
| FSWs | 1 (1) | 11.56 (7.31-17.82) | 0.00 | 1.00 | -- | -- | -- |
| MSM | -- | -- | -- | -- | -- | -- | -- |
| DUs | 1 (1) | 0.26 (0.02-4.06) | 0.00 | 1.00 | -- |  |  |
| ***Chlamydia*** | **10 (9)** |  |  |  |  |  |  |
| **Rural-to-urban migrants in various occupations** | 8 (8) | 2.81 (1.46-5.36) | 95.17 | <0.01‥ | 0.46 | 3.29 (2.74-3.95)[^10^](#_ENREF_10) | 0.9 (0.5-1.7) |
| Migrant women through marriage | 1 (1) | 0.26 (0.13-0.49) | 0.00 | 1.00 | -- | -- | -- |
| Construction workers | -- | -- | -- | -- | -- | -- | -- |
| Truck drivers | 1 (1) | 10.57 (8.22-13.48) | 0.00 | 1.00 | -- | -- | -- |
| Miners | 1 (1) | 9.34 (5.89-14.51) | 0.00 | 1.00 | -- | -- | -- |
| Factory workers | -- | -- | -- | -- | -- | -- | -- |
| Restaurant attendants | -- | -- | -- | -- | -- | -- | -- |
| Migrants with unspecified job nature | 5 (4) | 3.33 (2.69-4.11) | 29.21 | 0.23 | 0.05 | 3.29 (2.74-3.95)[^10^](#_ENREF_10) | 1.1 (0.8-1.6) |
| **Migrant pregnant women** | -- | -- | -- | -- | -- | -- | -- |
| **High-risk groups with a migratory background** |  |  |  |  |  |  |  |
| FSWs | 2 (2) | 15.34 (13.00-18.00) | 0.00 | 0.52 | -- | 17.30 (16.01-18.67)[^11^](#_ENREF_11) | 0.9 (0.7-1.1) |
| MSM | -- | -- | -- | -- | -- | -- | -- |
| DUs | -- | -- | -- | -- | -- | -- | -- |

| **Table S7 Prevalence and odds ratios of sexually transmitted and viral hepatitis infections among rural-to-urban migrants, migrant pregnant women and high-risk groups with a migratory background compared with national prevalence or systematic review studies among background population (Cont’d)** | | | | | | | | |
| --- | --- | --- | --- | --- | --- | --- | --- | --- |
| **Subgroups** | **No. of estimates (No. of studies)** | **Pooled Prevalence (%) and 95% CI** | **Heterogeneity** | | **Publication bias (*p* value)** | | **Background prevalence (%) and 95% CI†** | **Odds Ratio (95% CI)** |
|  |  |  | ***I^2^*** | ***p*** |  |  |  |  |
| ***Genital warts*** | **6 (6)** |  |  |  |  | |  |  |
| **Rural-to-urban migrants in various occupations** | 6 (6) | 1.54 (0.70-3.36) | 91.91 | <0.01‥ | 0.57 | | 0.04 (0.01-0.07)[^12^](#_ENREF_12) | 38.5 (15.7-94.5)* |
| Migrant women through marriage | 2 (2) | 1.23 (0.91-1.64) | 0.00 | 0.48 | | -- | 0.04 (0.01-0.07)[^12^](#_ENREF_12) | 30.6 (14.4-64.9)* |
| Construction workers | 1 (1) | 0.42 (0.29-0.63) | 0.00 | 1.00 | | -- | -- | -- |
| Truck drivers | -- | -- | -- | -- | | -- | -- | -- |
| Miners | -- | -- | -- | -- | | -- | -- | -- |
| Factory workers | -- | -- | -- | -- | | -- | -- | -- |
| Restaurant attendants | -- | -- | -- | -- | | -- | -- | -- |
| Migrants with unspecified job nature | 3 (3) | 3.28 (2.33-4.60) | 0.00 | 0.41 | | 0.60 | 0.04 (0.01-0.07)[^12^](#_ENREF_12) | 82.0 (38.1-176.6)* |
| **Migrant pregnant women** | -- | -- | -- | -- | | -- | -- | -- |
| **High-risk groups with a migratory background** | -- | -- | -- | -- | | -- | -- | -- |
| FSWs | -- | -- | -- | -- | | -- | -- | -- |
| MSM | -- | -- | -- | -- | | -- | -- | -- |
| DUs | -- | -- | -- | -- | | -- | -- | -- |
| ***HPV*** | **4 (4)** |  |  |  | |  |  |  |
| **Rural-to-urban migrants in various occupations** | 3 (3) | 12.39 (6.11-23.51) | 98.18 | <0.01‥ | | 0.60 | 16.80 (16.50-17.10)[^13^](#_ENREF_13) | 0.7 (0.4-1.3) |
| Migrant women through marriage | 1 (1) | 17.00 (10.84-25.66) | 0.00 | 1.00 | | -- | -- | -- |
| Construction workers | -- | -- | -- | -- | | -- | -- | -- |
| Truck drivers | -- | -- | -- | -- | | -- | -- | -- |
| Miners | -- | -- | -- | -- | | -- | -- | -- |
| Factory workers | 1 (1) | 6.92 (6.26-7.64) | 0.00 | 1.00 | | -- | -- | -- |
| Restaurant attendants | -- | -- | -- | -- | | -- | -- | -- |
| Migrants with unspecified job nature | 1 (1) | 16.36 (14.37-18.56) | 0.00 | 1.00 | | -- | -- | -- |
| **Pregnant women with a migratory background** | -- | -- | -- | -- | | -- | -- | -- |
| **High-risk groups with a migratory background** |  |  |  |  | |  |  |  |
| FSWs | -- | -- | -- | -- | | -- | -- | -- |
| MSM | 1 (1) | 69.84 (57.49-79.86) | 0.00 | 1.00 | | -- | -- | -- |
| DUs | -- | -- | -- | -- | | -- | -- | -- |

| **Table S7 Prevalence and odds ratios of sexually transmitted and viral hepatitis infections among rural-to-urban migrants, migrant pregnant women and high-risk groups with a migratory background compared with national prevalence or systematic review studies among background population (Cont’d)** | | | | | | | |
| --- | --- | --- | --- | --- | --- | --- | --- |
| **Subgroups** | **No. of estimates (No. of studies)** | **Pooled Prevalence (%) and 95% CI** | **Heterogeneity** | | **Publication bias (*p* value)** | **Background prevalence (%) and 95% CI†** | **Odds Ratio (95% CI)** |
|  |  |  | ***I^2^*** | ***p*** |  |  |  |
| ***HSV*** | **11 (10)** |  |  |  |  |  |  |
| **Rural-to-urban migrants in various occupations** | 8 (8) | 3.34 (1.22-8.79) | 97.92 | <0.01 | <0.05 | 5.80 (3.82-7.78)[^14^](#_ENREF_14) | 0.6 (0.3-1.2) |
| Migrant women through marriage | -- | -- | -- | -- | -- | -- | -- |
| Construction workers | 1 (1) | 0.25 (0.15-0.42) | 0.00 | 1.00 | -- | -- | -- |
| Truck drivers | 1 (1) | 4.40 (2.97-6.49) | 0.00 | 1.00 | -- | -- | -- |
| Miners | -- | -- | -- | -- | -- | -- | -- |
| Factory workers | -- | -- | -- | -- | -- | -- | -- |
| Restaurant attendants | -- | -- | -- | -- | -- | -- | -- |
| Migrants with unspecified job nature | 6 (6) | 5.63 (2.48-12.29) | 95.90 | <0.01‥ | 0.57 | 5.80 (3.82-7.78)[^14^](#_ENREF_14) | 0.4 (0.2-1.2) |
| **Pregnant women with a migratory background** | -- | -- | -- | -- | -- | -- | -- |
| **High-risk groups with a migratory background** |  |  |  |  |  |  |  |
| FSWs | 3(2) | 42.52 (30.51-55.50) | 86.68 | 0.01‥ | 0.12 | 50.19 (33.47-66.87)[^15^](#_ENREF_15) | 0.8 (0.4-1.8) |
| MSM | -- | -- | -- | -- | -- | -- | -- |
| DUs | -- | -- | -- | -- | -- | -- | -- |
| ***HBV*** | **65 (43)** |  |  |  |  |  |  |
| **Rural-to-urban migrants in various occupations** | 56(38) | 8.51 (7.59-9.53) | 99.65 | <0.01‥ | 0.58 | 6.40 (4.45-9.08) | 1.3 (1.0-1.9) |
| Migrant women through marriage | 3 (3) | 9.14 (5.77-14.17) | 90.53 | <0.01‥ | 0.60 | 6.40 (4.45-9.08) | 1.4 (0.9-2.4) |
| Construction workers | 4 (4) | 13.70 (11.84-15.80) | 0.00 | 0.90 | 0.17 | 6.40 (4.45-9.08) | 2.1 (1.5-3.1)* |
| Truck drivers | 1 (1) | 12.23 (9.26-15.99) | 0.00 | 1.00 | -- | -- | -- |
| Miners | -- | -- | -- | -- | -- | -- | -- |
| Factory workers | 1 (1) | 3.87 (2.21-6.69) | 0.00 | 1.00 | -- | -- | -- |
| Restaurant attendants | -- | -- | -- | -- | -- | -- | -- |
| Migrants with unspecified job nature | 47(29) | 8.22 (7.25-9.31) | 99.46 | <0.01‥ | 0.32 | 6.40 (4.45-9.08) | 1.3 (0.9-1.8) |
| **Pregnant women with a migratory background** | 7(3) | 3.49 (1.70-7.05) | 99.15 | <0.01‥ | 0.10 | 5.49 (4.83-6.15)[^19^](#_ENREF_19) | 0.6 (0.3-1.2) |
| **High-risk groups with a migratory background** |  |  |  |  |  |  |  |
| FSWs | -- | -- | -- | -- | -- | -- | -- |
| MSM | -- | -- | -- | -- | -- | -- | -- |
| DUs | 2 (2) | 11.91 (7.42-18.56) | 0.00 | 0.66 | -- | 12.6 (11.18-14.02)[^20^](#_ENREF_20) | 1.0 (0.6-1.5) |

| **Table S7 Prevalence and odds ratios of sexually transmitted and viral hepatitis infections among rural-to-urban migrants, migrant pregnant women and high-risk groups with a migratory background compared with national prevalence or systematic review studies among background population (Cont’d)** | | | | | | | | |
| --- | --- | --- | --- | --- | --- | --- | --- | --- |
| **Subgroups** | **No. of estimates (No. of studies)** | **Pooled Prevalence (%) and 95% CI** | **Heterogeneity** | | | **Publication bias (*p* value)** | **Background prevalence (%) and 95% CI†** | **Odds Ratio (95% CI)** |
|  |  |  | ***I^2^*** | ***p*** | |  |  |  |
| ***HCV*** | **71 (58)** |  |  |  | |  |  |  |
| **Rural-to-urban migrants in various occupations** | 47 (37) | 0.45 (0.31-0.65) | 88.52 | <0.01‥ | | 0.84 | 0.12 (0.05-0.27) | 3.8 (1.9-7.3)* |
| Migrant women through marriage | 3 (3) | 1.97 (1.00-3.85) | 70.29 | 0.03 | | 0.60 | 0.12 (0.05-0.27) | 16.4 (7.6-35.5)* |
| Construction workers | 10 (10) | 0.85 (0.64-1.13) | 0.00 | 0.52 | | 0.09 | 0.12 (0.05-0.27) | 7.1 (3.8-13.4)* |
| Truck drivers | 3 (3) | 1.01 (0.57-1.76) | 0.00 | 0.73 | | 0.12 | 0.12 (0.05-0.27) | 8.4 (4.0-17.8)* |
| Miners | -- | -- | -- | | -- | -- | -- | -- |
| Factory workers | 5 (5) | 0.22 (0.07-0.69) | 0.00 | | 0.87 | 0.33 | 0.12 (0.05-0.27) | 1.8 (0.8-4.5) |
| Restaurant attendants | 1 (1) | 0.12 (0.01-1.96) | 0.00 | | 1.00 | -- | -- | -- |
| Migrants with unspecified job nature | 25 (21) | 0.39 (0.31-0.49) | 20.42 | | 0.18 | 0.01^＊＊^ | 0.12 (0.05-0.27) | 3.3 (1.8-6.0)* |
| **Pregnant women with a migratory background** | 5 (2) | 0.05 (0.02-0.15) | 62.63 | | 0.03• | 0.59 | 0.15 (0.08-0.22)[^22^](#_ENREF_22) | 0.3 (0.2-0.6)* |
| **High-risk groups with a migratory background** |  |  |  | |  |  |  |  |
| FSWs | 6 (6) | 0.30 (0.11-0.86) | 0.00 | | 0.99 | 0.56 | 1.00 (0.83-1.17)[^23^](#_ENREF_23) | 0.3 (0.2-0.6)* |
| MSM | 5 (5) | 1.25 (0.37-4.10) | 75.23 | | <0.01‥ | <0.01^＊＊^ | 1.25 (1.00-1.55) | 1.0 (0.5-2.1) |
| DUs | 8 (8) | 48.95 (42.03-55.92) | 90.98 | | <0.01‥ | 0.62 | 51.71 (51.29-52.13)[^24^](#_ENREF_24) | 1.0 (0.7-1.1) |

† rural-to-urban migrants of various occupations were compared with the general population; pregnant women with migrant background were compared with general pregnant women; the drug users (DUs) with a migratory background were compared with the general DUs; the female sex workers (FSWs) with a migratory background were compared with general FSWs; the men have sex with men (MSM) with a migratory background were compared with the general MSM;

*denote the odds ratios were significant comparing the migrants with background populations; ‥*p* value of heterogeneity test was less than 0.01; • p value of heterogeneity test was significantly less than 0.05; ^＊＊^*p* value of publication bias (Begg’s test) was less than 0.01; ^＊^p value of publication bias (Begg’s test) was less than 0.05.

**Reference:**

1. China NHaFPCotPsRo, UNAIDS, WHO. 2011 Estimates for the HIV/AIDS Epidemic in China. Beijing2011.

2. Song J, Feng T, Bulterys M, et al. An integrated city-driven perinatal HIV prevention program covering 1.8 million pregnant women in Shenzhen, China, 2000 to 2010. Sex Transm Dis. Apr 2013;40(4):329-334.

3. China NHaFPCotPsRo, UNAIDS, WHO. 2012 China AIDS Response Progress Report2012.

4. Zhuang X, Liang Y, Chow EP, Wang Y, Wilson DP, Zhang L. HIV and HCV prevalence among entrants to methadone maintenance treatment clinics in China: a systematic review and meta-analysis. BMC Infect Dis. 2012;12:130.

5. Hesketh T, Ye X, Zhu W. Syphilis in China: the great comeback. Emerg Health Threats J. 2008;1:e6.

6. Lin CC, Gao X, Chen XS, Chen Q, Cohen MS. China's syphilis epidemic: a systematic review of seroprevalence studies. Sex Transm Dis. Dec 2006;33(12):726-736.

7. Chen XS, Wang QQ, Yin YP, et al. Prevalence of syphilis infection in different tiers of female sex workers in China: implications for surveillance and interventions. BMC Infect Dis. 2012;12:84.

8. Chow EP, Iu KI, Fu X, Wilson DP, Zhang L. HIV and sexually transmissible infections among money boys in China: a data synthesis and meta-analysis. PLoS One. 2012;7(11):e48025.

9. Chow EPF, Tucker JD, Wong FY, et al. Disparities and Risks of Sexually Transmissible Infections among Men Who Have Sex with Men in China: A Meta-Analysis and Data Synthesis. PLoS One. 2014;9(2):e89959.

10. Parish WL, Laumann EO, Cohen MS, et al. Population-based study of chlamydial infection in China: a hidden epidemic. JAMA. Mar 12 2003;289(10):1265-1273.

11. Chen XS, Yin YP, Liang GJ, et al. The prevalences of Neisseria gonorrhoeae and Chlamydia trachomatis infections among female sex workers in China. BMC Public Health. 2013;13:121.

12. Wu C, Chen G, Li H, Liang G, Li C. Hepatitis B and syphilis and condyloma acuminatum infection among public service workers in Zhanjiang. South China Journal of Preventive Medicine. 2006;32(2):46-47.

13. Zhao FH, Lewkowitz AK, Hu SY, et al. Prevalence of human papillomavirus and cervical intraepithelial neoplasia in China: a pooled analysis of 17 population-based studies. Int J Cancer. Dec 15 2012;131(12):2929-2938.

14. Lu F, Jia Y, Bin S, et al. Predictors for casual sex and/or infection among sexually transmitted disease clinic attendees in China. Int J STD AIDS. Apr 2009;20(4):241-248.

15. Poon AN, Li Z, Wang N, Hong Y. Review of HIV and other sexually transmitted infections among female sex workers in China. AIDS Care. Jun 2011;23 Suppl 1:5-25.

16. Xiao H, Cao J, Li J, Huang H, Zhang W, Cao Z. [Prevalence of HIV, HBV and HCV among drug users and general population]. Chinese Medical Science & Health. 2007;6:7-8.

17. Yu Q, Yang Y, Liu R, Chun Z, Zhang J. [Investigation on HBV, syphilis and HIV infection among high-risk population in Dunhuang city of Gansu province]. Strait Journal of Preventive Medicine. 2007;13(6):45.

18. Luo Z, Xie Y, Deng M, Zhou X, Ruan B. Prevalence of hepatitis B in the southeast of China: a population-based study with a large sample size. Eur J Gastroenterol Hepatol. Aug 2011;23(8):695-700.

19. Ding Y, Sheng Q, Ma L, Dou X. Chronic HBV infection among pregnant women and their infants in Shenyang, China. Virol J. 2013;10:17.

20. Zhao YS, Su SI, Lv CX, et al. Seroprevalence of hepatitis C, hepatitis B virus and syphilis in HIV-1 infected patients in Shandong, China. Int J STD AIDS. Sep 2012;23(9):639-643.

21. Dai S, Shen Z, Zha Z, et al. Seroprevalence of HIV, syphilis, and hepatitis C virus in the general population of the Liangshan Prefecture, Sichuan Province, China. J Med Virol. Jan 2012;84(1):1-5.

22. Xu J, Huang W. Prevalence analysis of four kinds of transmitted diseases among 13113 pregnant women. International Medicine and Health Guidance News. 2010;16(20):2507-2509.

23. Zhou Y, Li X, Zhang C, et al. Rates of HIV, syphilis, and HCV infections among different demographic groups of female sex workers in Guangxi China: Evidence from 2010 national sentinel surveillance data. AIDS Care. Feb 25 2013.

24. Bao YP, Liu ZM. Systematic review of HIV and HCV infection among drug users in China. Int J STD AIDS. Jun 2009;20(6):399-405.

**Table S8 Comparison of prevalence levels of infections of low-risk subgroup population between low- and high-quality groups**

| **Disease** | **Quality score ≤4 (%)** | **Quality score >4 (%)** | ***Z*** | ***P*** |
| --- | --- | --- | --- | --- |
| HIV | 0.00 (0.00-0.39) | 0.00 (0.00-0.25) | -0.733 | 0.464 |
| Syphilis | 0.75 (0.22-1.58) | 0.44 (0.09-0.95) | -1.772 | 0.076 |
| Gonorrhea | 1.43 (0.13-4.19) | 2.21 (0.73-6.99) | -0.510 | 0.683 |
| Chlamydia | 1.71 (0.85-2.63) | 3.46 (1.81-7.06) | -1.061 | 0.400* |
| Genital warts | 2.00 (0.72-3.44) | —— | -0.293 | 1.000* |
| HSV | 4.27(0.38-12.95) | 4.94 (1.29-21.23) | -1.225 | 0.667* |
| HPV | —— | 11.64 (6.92-16.36) | 0.000 | 1.000* |
| HBV | 10.34 (7.60-12.94) | 7.06 (3.80-12.91) | -1.469 | 0.142 |
| HCV | 0.39 (0.25-1.03) | 0. 25(0.00-0.60) | -1.574 | 0.116 |

* Fisher’s exact test

**Figure S3-1a Forest plots of meta-analysis of HIV prevalence in individual occupational subgroups of rural-to-urban migrants in China**

**
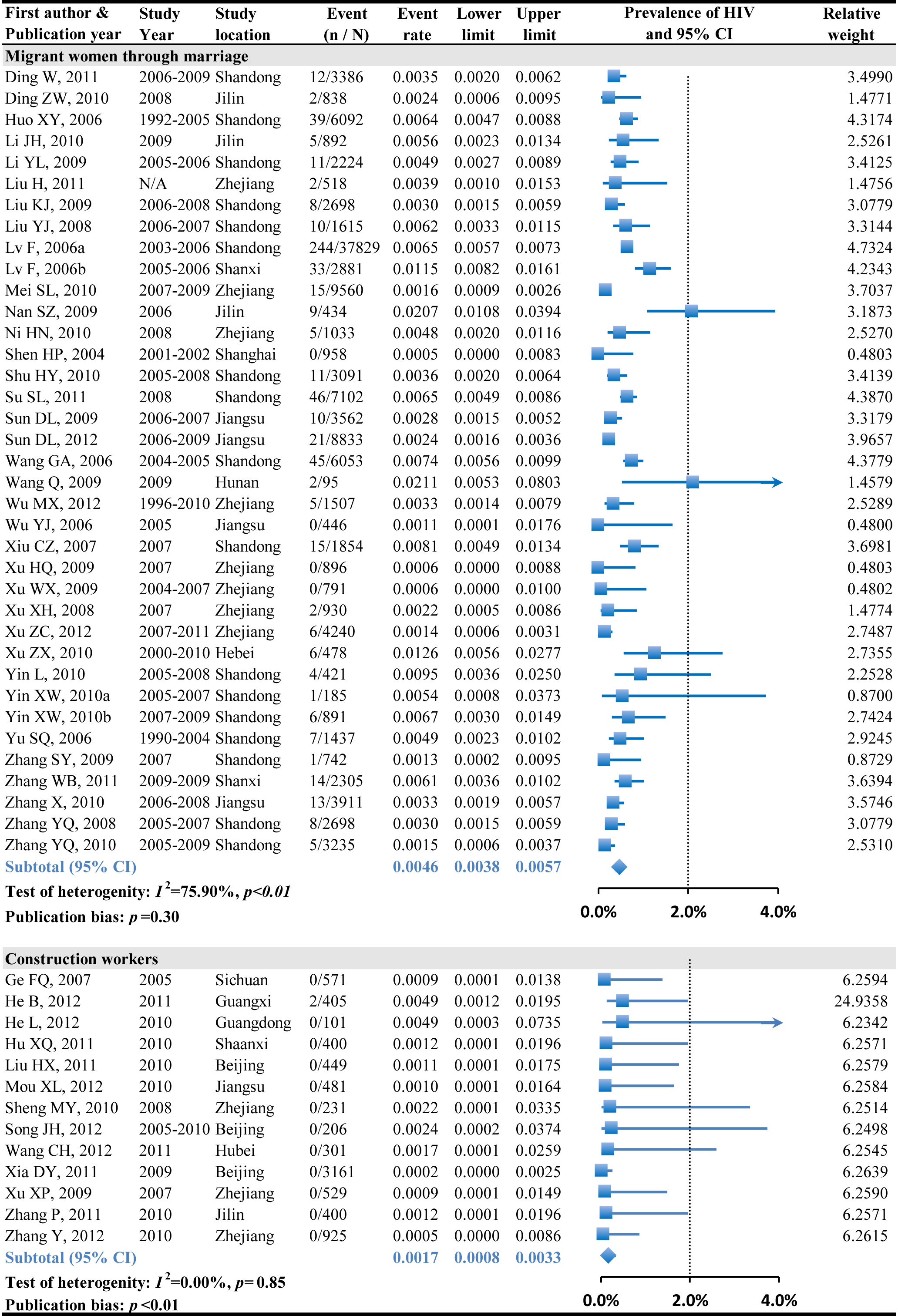
**

**Figure S3-1a Forest plots of meta-analysis of HIV prevalence in individual occupational subgroups of rural-to-urban migrants in China (Cont’d)
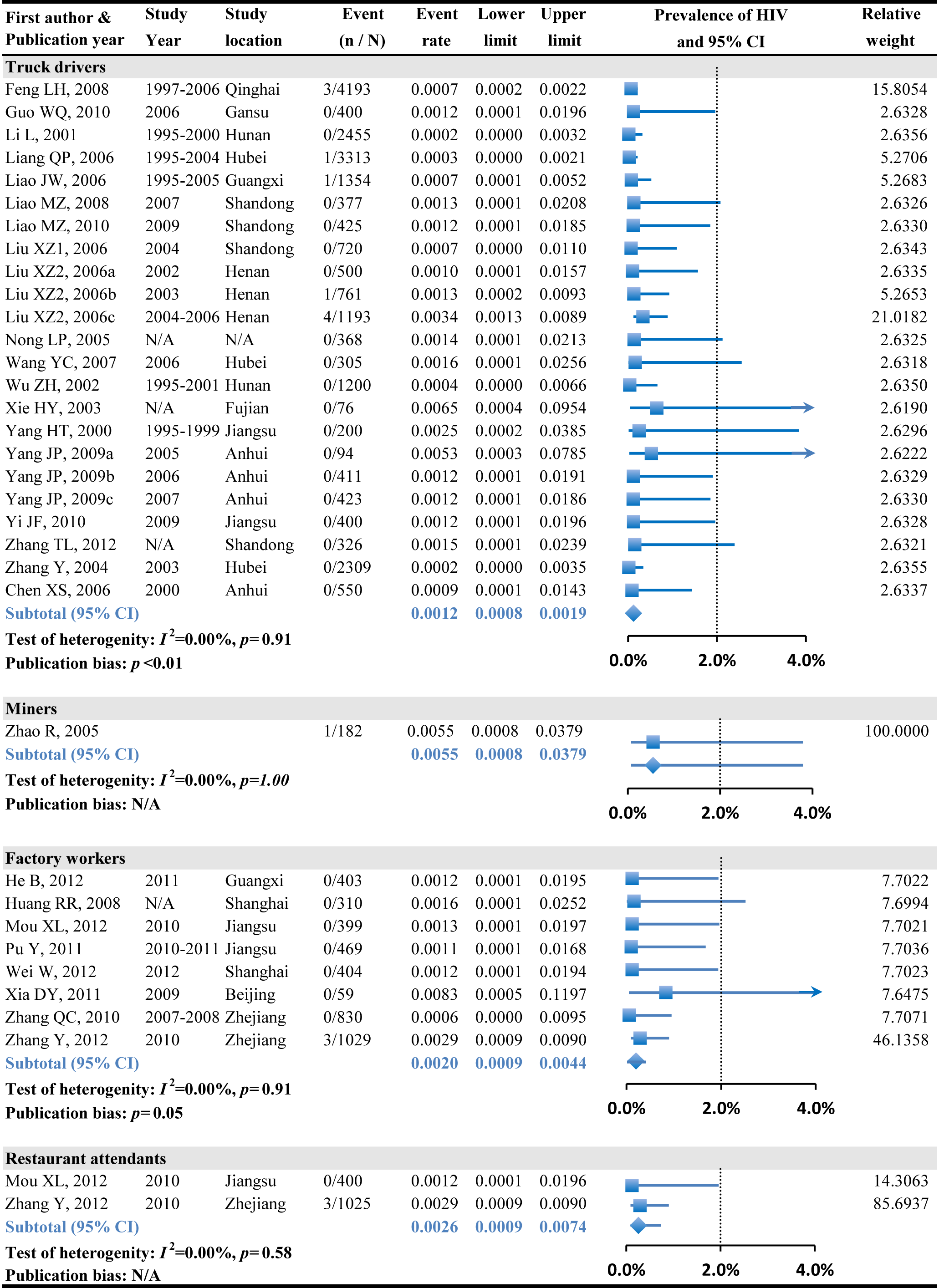
**

**Figure S3-1a Forest plots of meta-analysis of HIV prevalence in individual occupational subgroups of rural-to-urban migrants in China (Cont’d)**

**
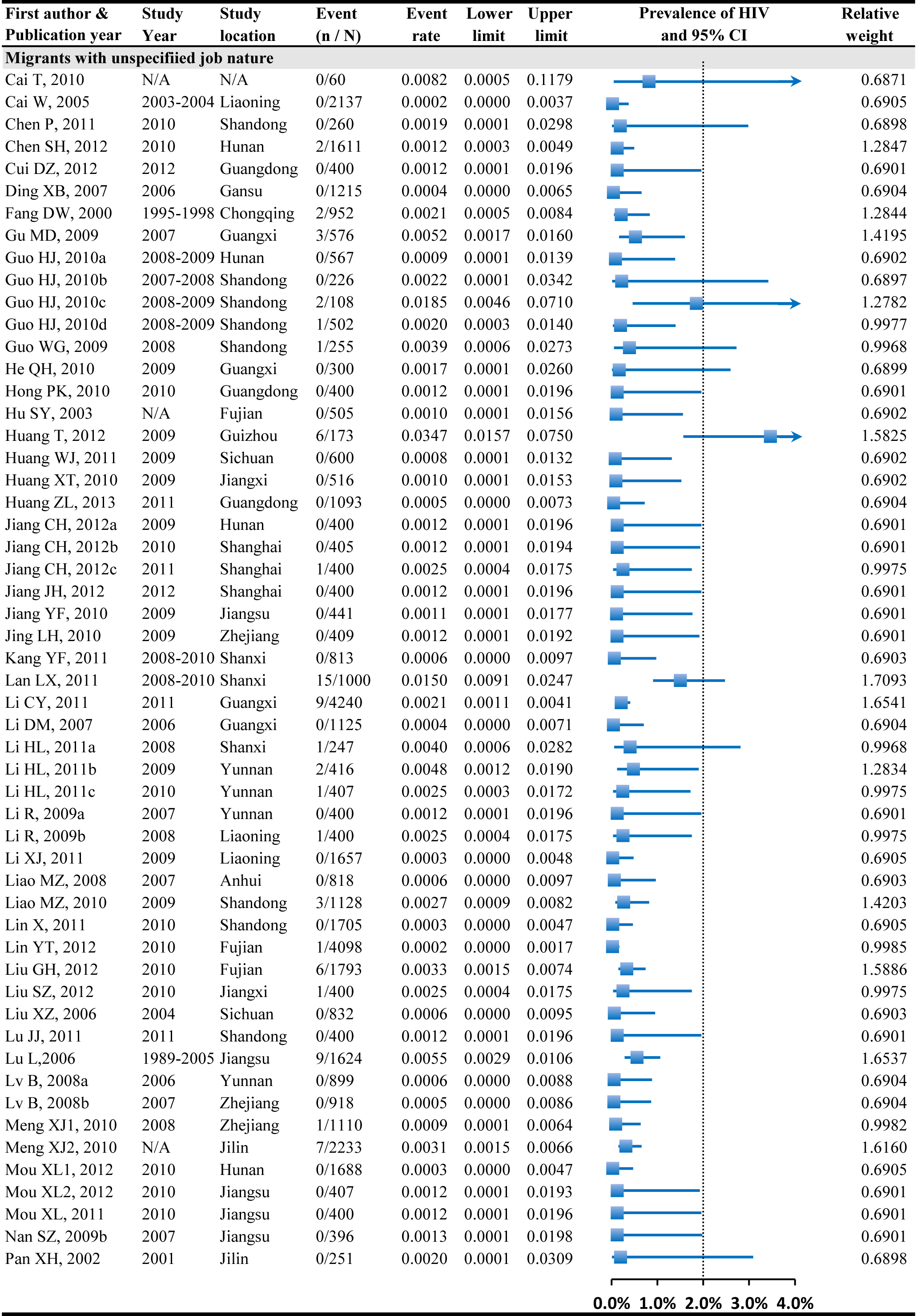
**

**Figure S3-1a Forest plots of meta-analysis of HIV prevalence in individual occupational subgroups of rural-to-urban migrants in China (Cont’d)**

**
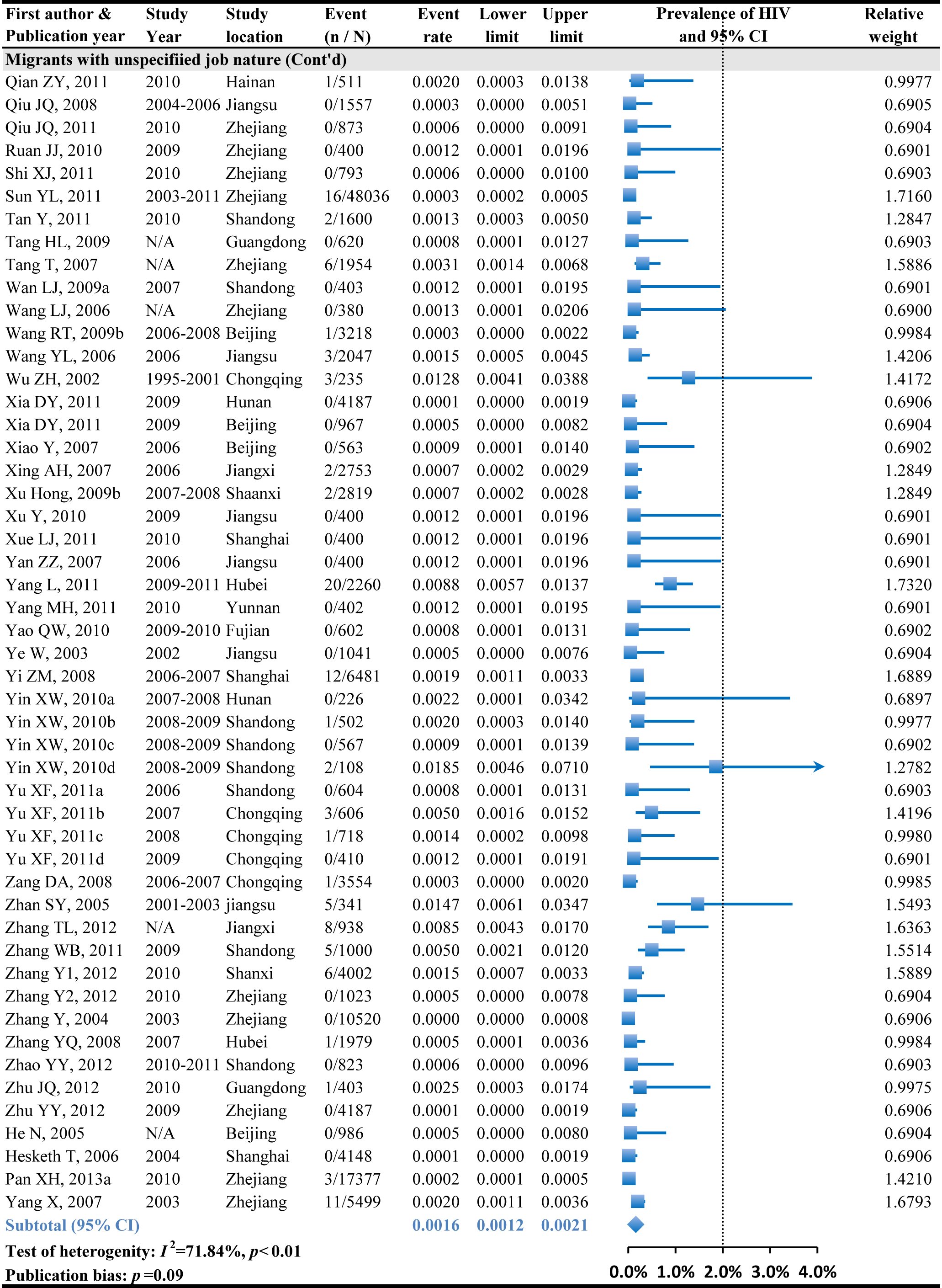
**

**Figure S3-1b Overall pooled estimate of HIV prevalence in rural-to-urban migrants in China**

**
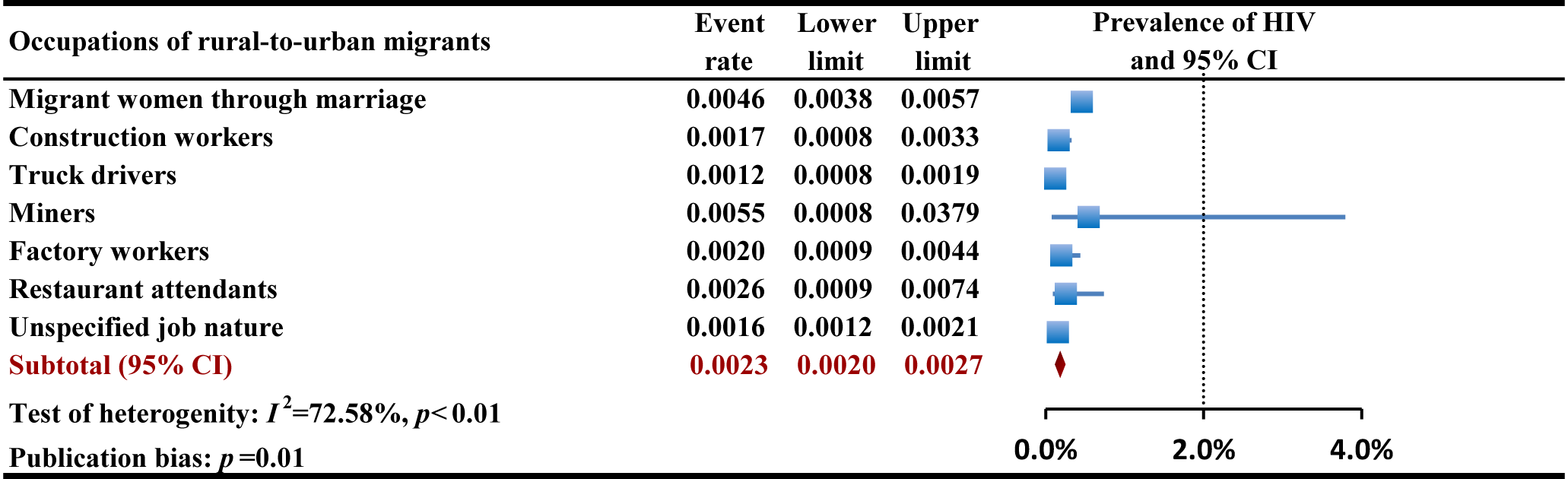
**

**Figure S3-1c Forest plots of meta-analysis of HIV prevalence in migrant pregnant women in China**

**
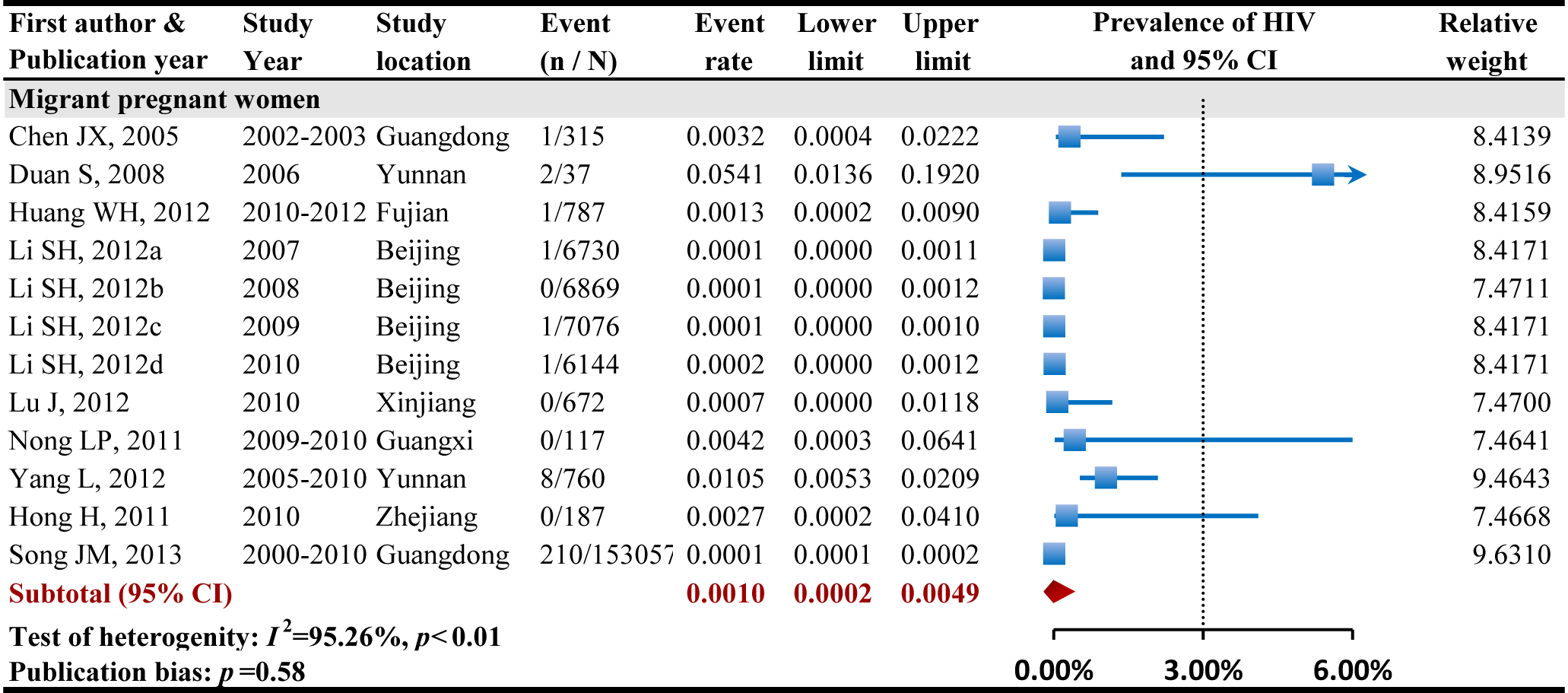
**

**Figure S3-1d Forest plots of meta-analysis of HIV prevalence in most at-risk populations with a migratory background in China**

**
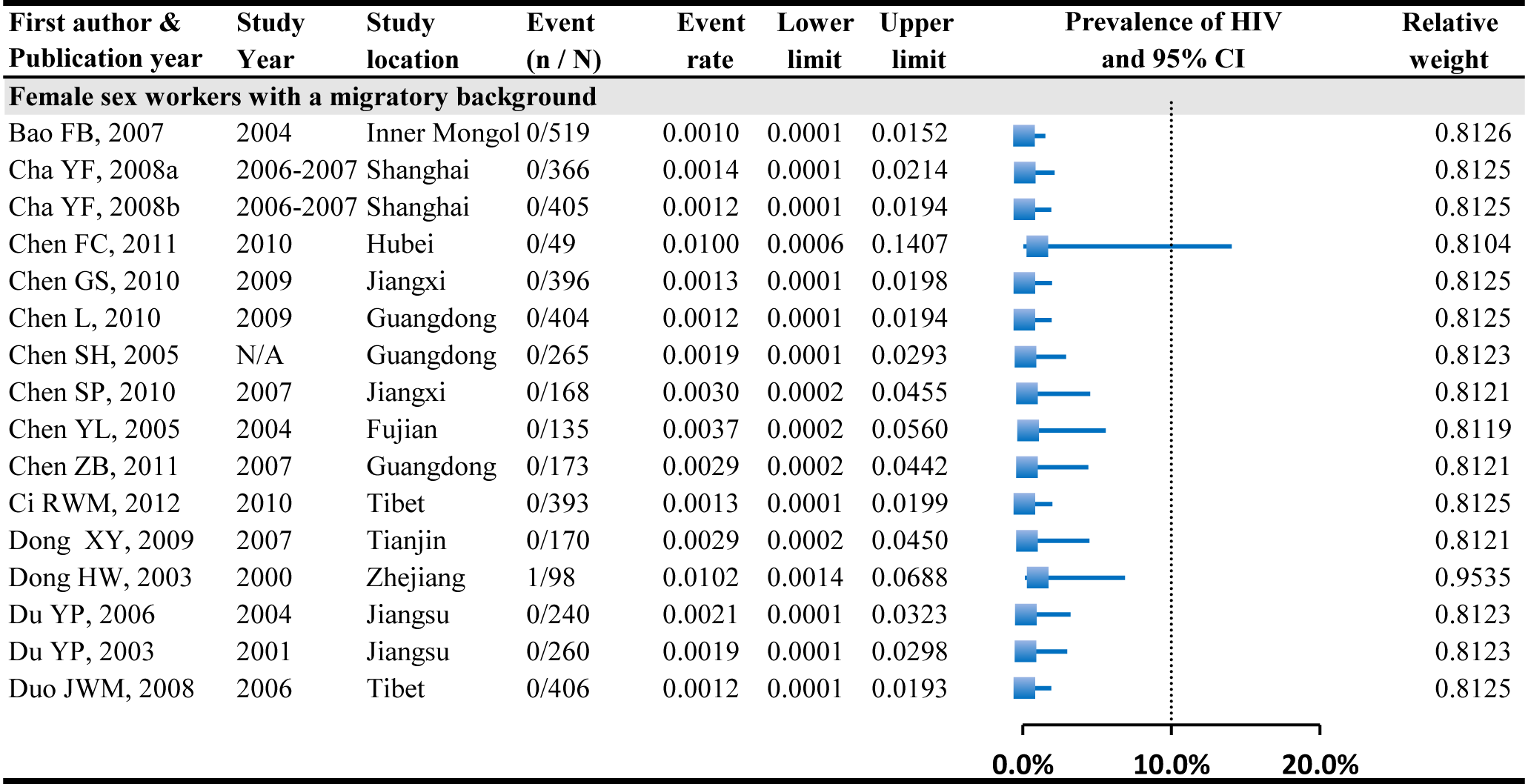
**

**Figure S3-1d Forest plots of meta-analysis of HIV prevalence in most at-risk populations with a migratory background in China (Cont’d)**

**
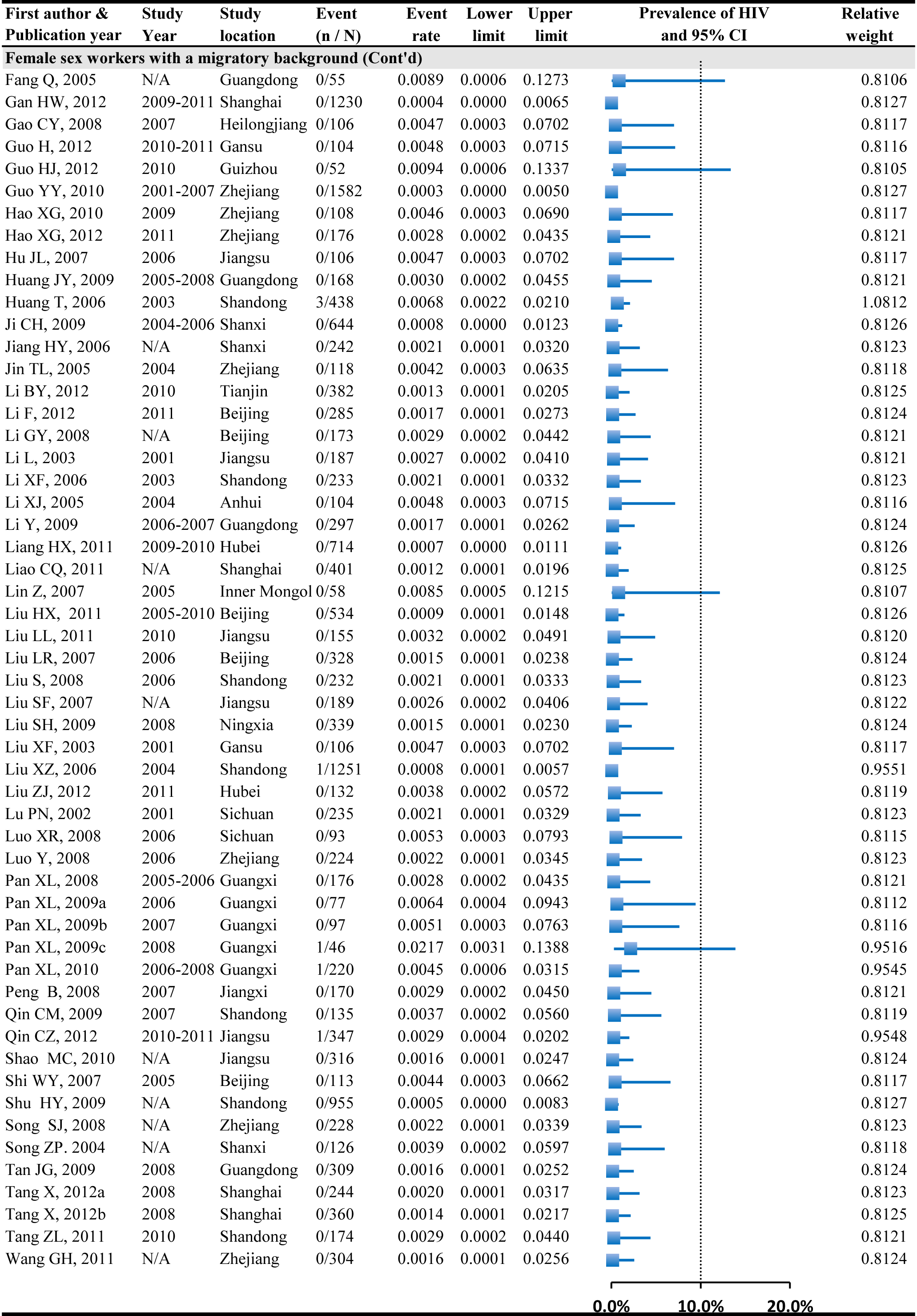
Figure S3-1d Forest plots of meta-analysis of HIV prevalence in most at-risk populations with a migratory background in China (Cont’d)**

**
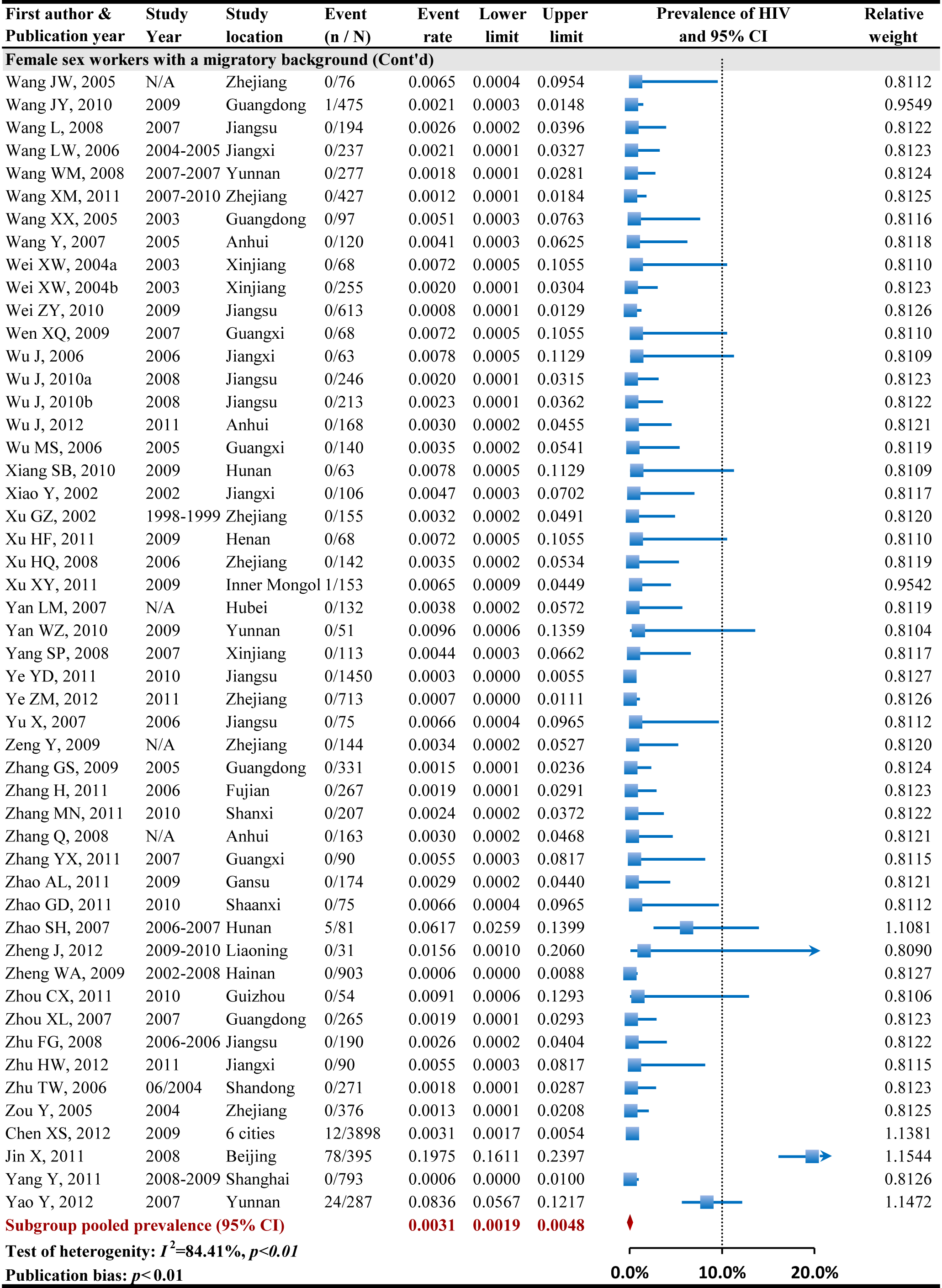
Figure S3-1d Forest plots of meta-analysis of HIV prevalence in most at-risk populations with a migratory background in China (Cont’d)**

**
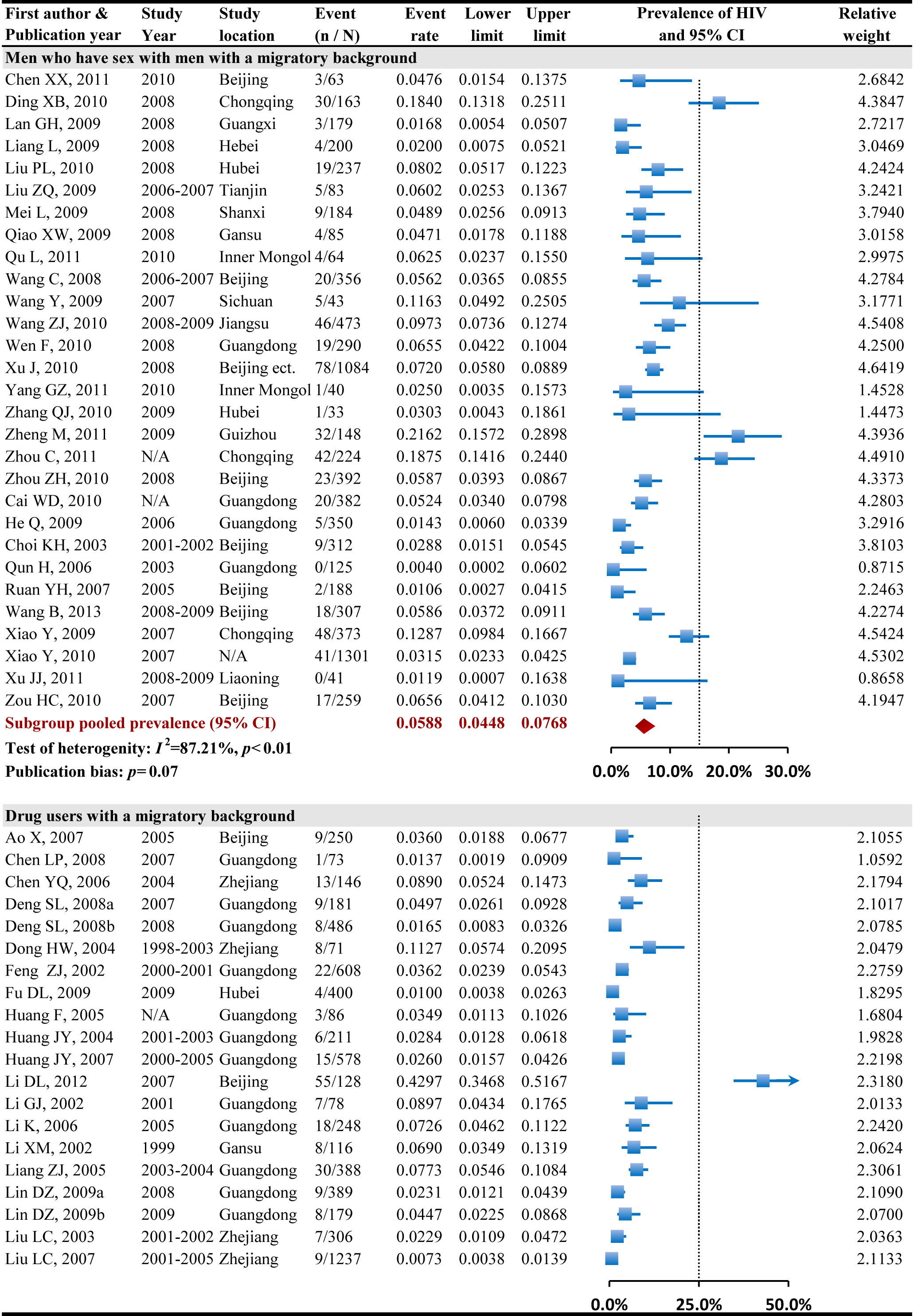
**

**Figure S3-1d Forest plots of meta-analysis of HIV prevalence in most at-risk populations with a migratory background in China (Cont’d)**

**
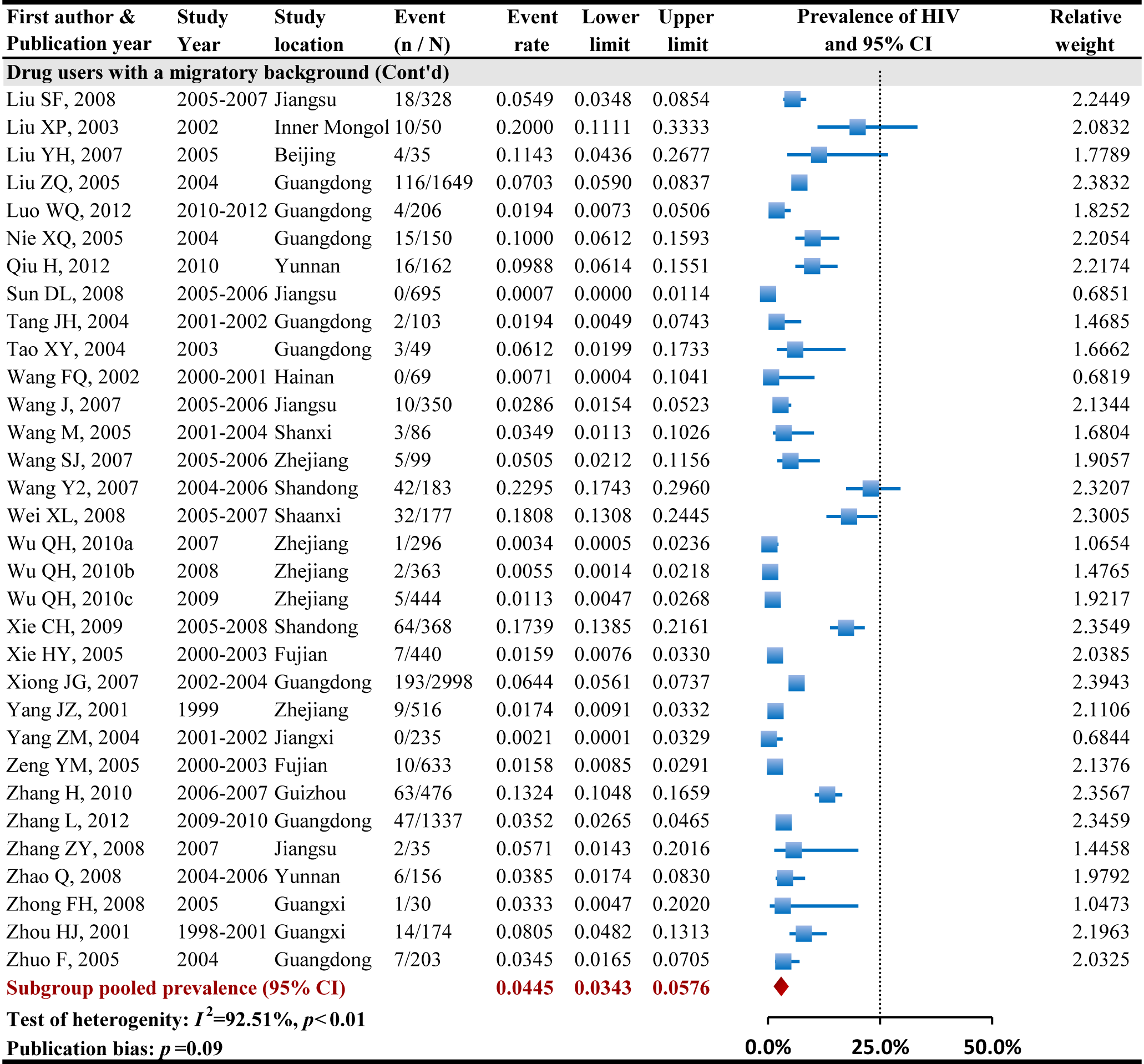
**

**Figure S3-2a Forest plots of meta-analysis of syphilis prevalence in individual occupational subgroups of rural-to-urban migrants in China**

**
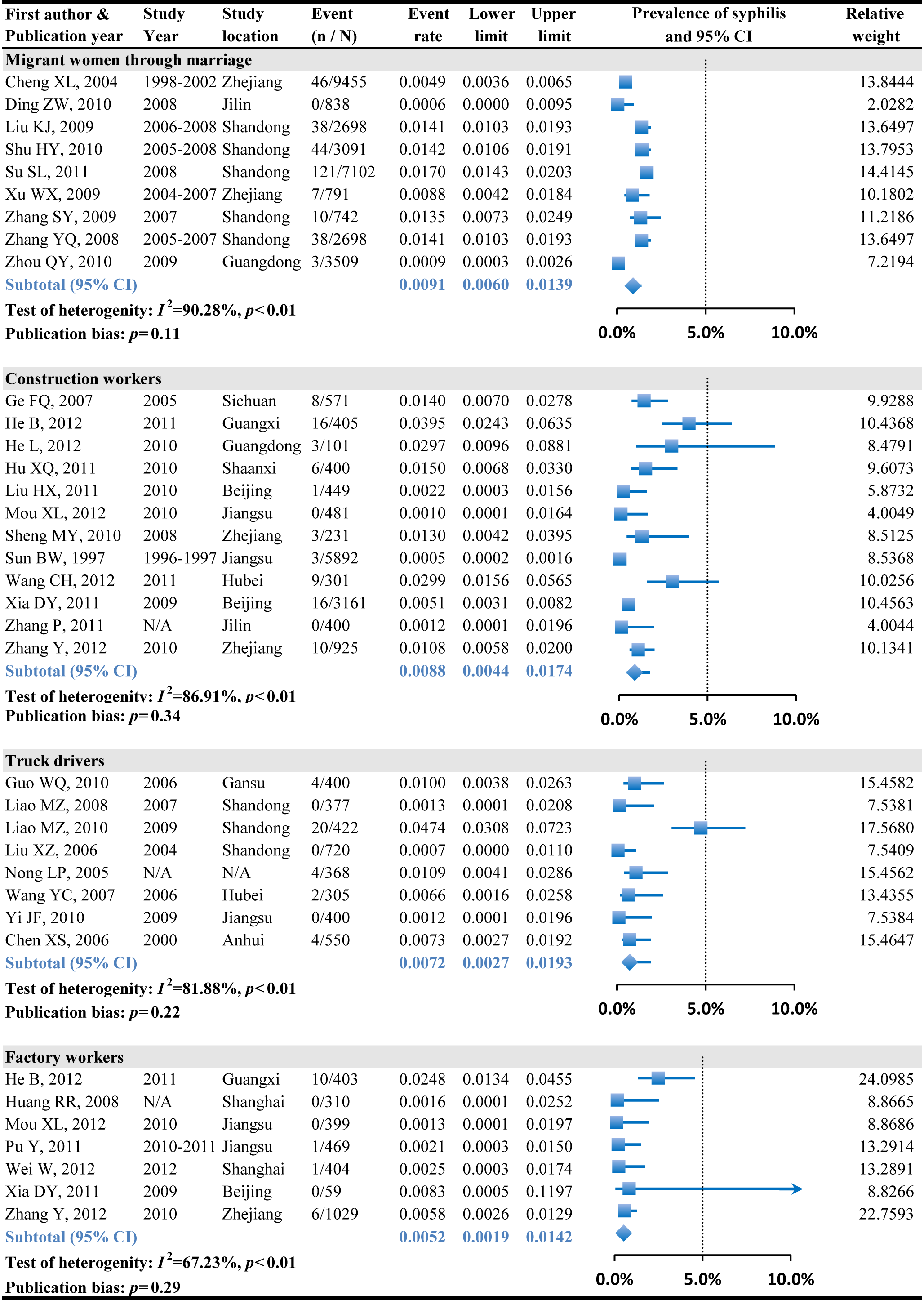
Figure S3-2a Forest plots of meta-analysis of syphilis prevalence in individual occupational subgroups of rural-to-urban migrants in China (Cont’d)**

**
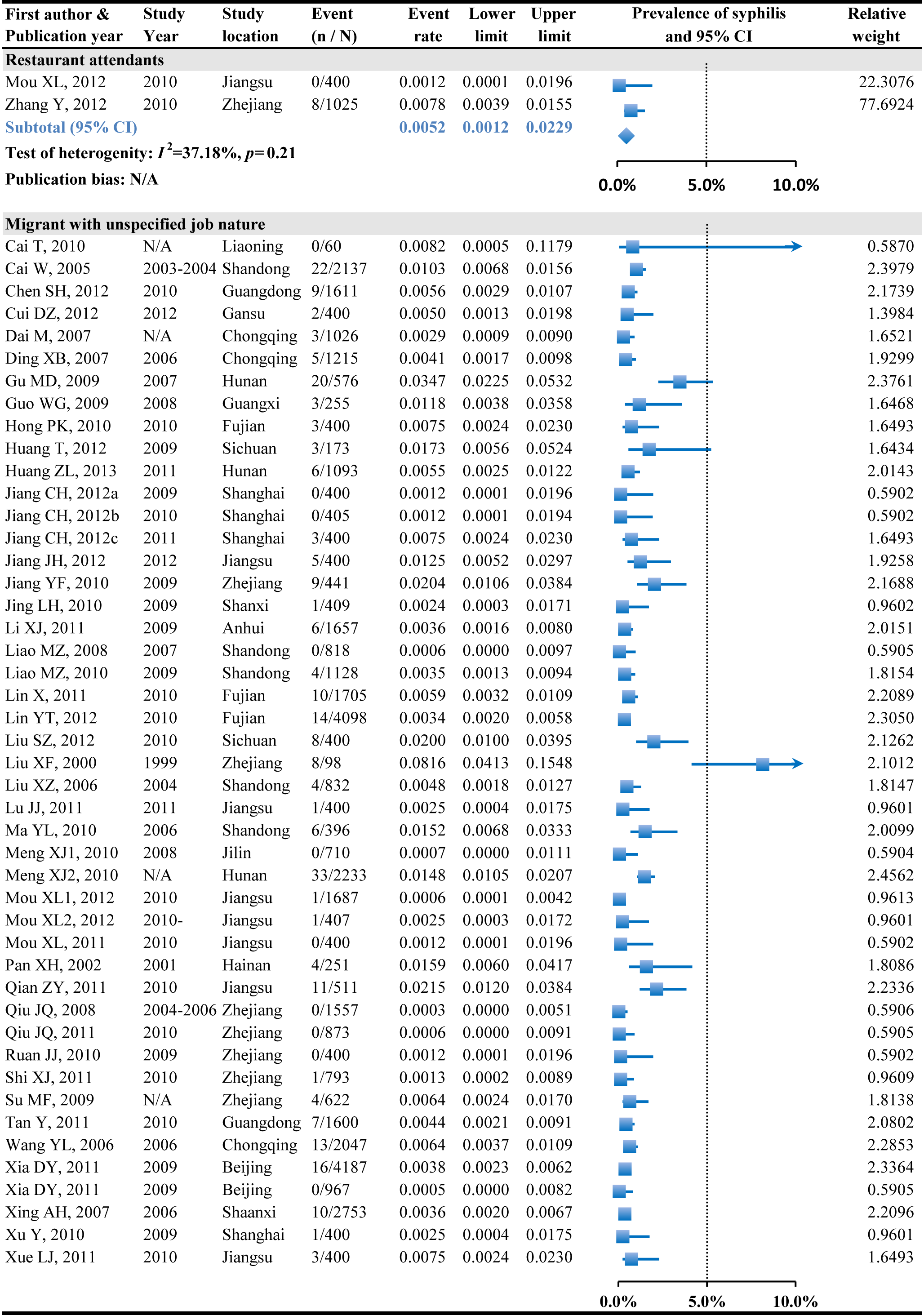
**

**Figure S3-2a Forest plots of meta-analysis of syphilis prevalence in individual occupational subgroups of rural-to-urban migrants in China (Cont’d)**

**
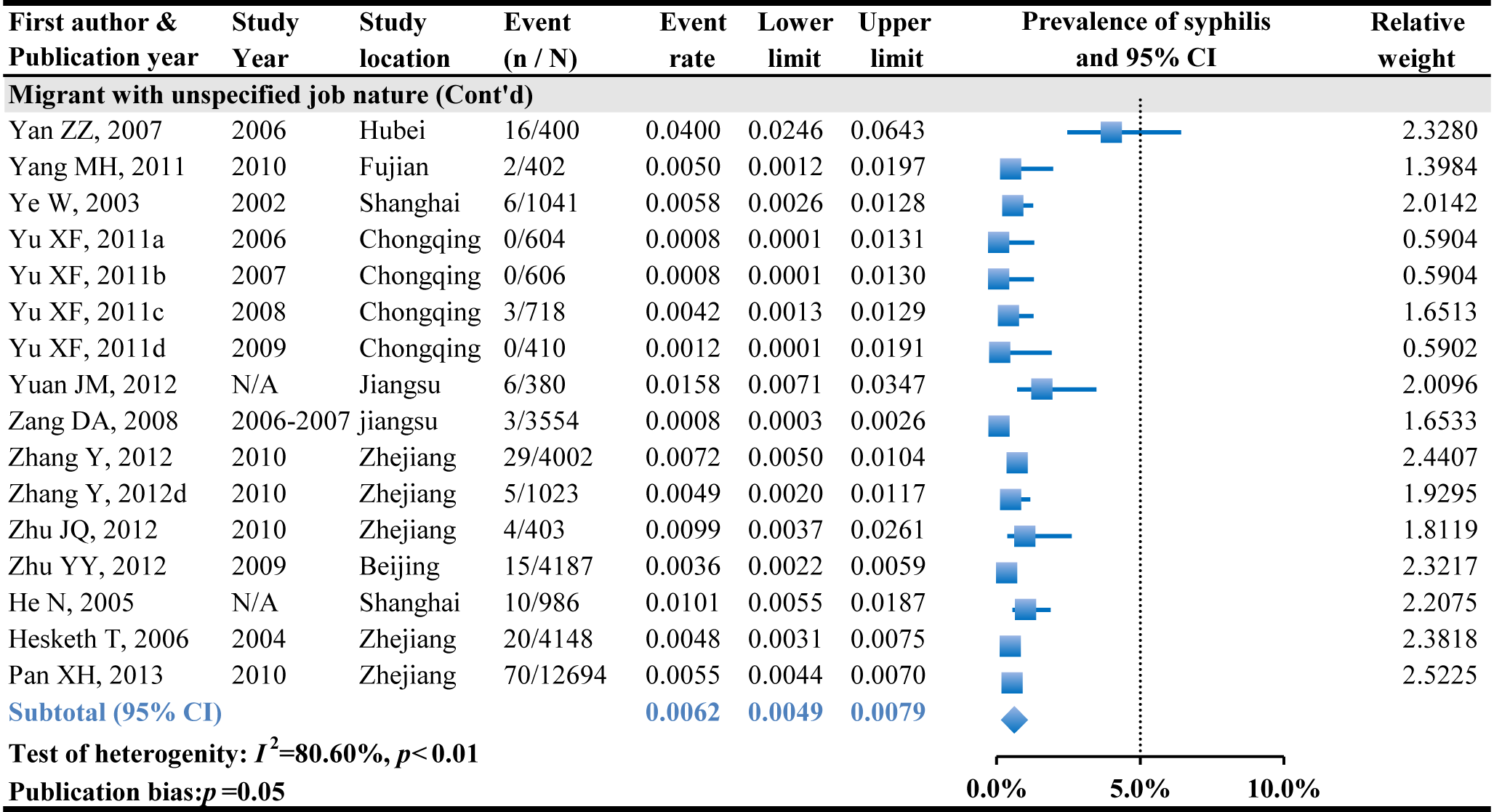
**

**Figure S3-2b Overall pooled estimate of syphilis prevalence in rural-to-urban migrants in China**

**
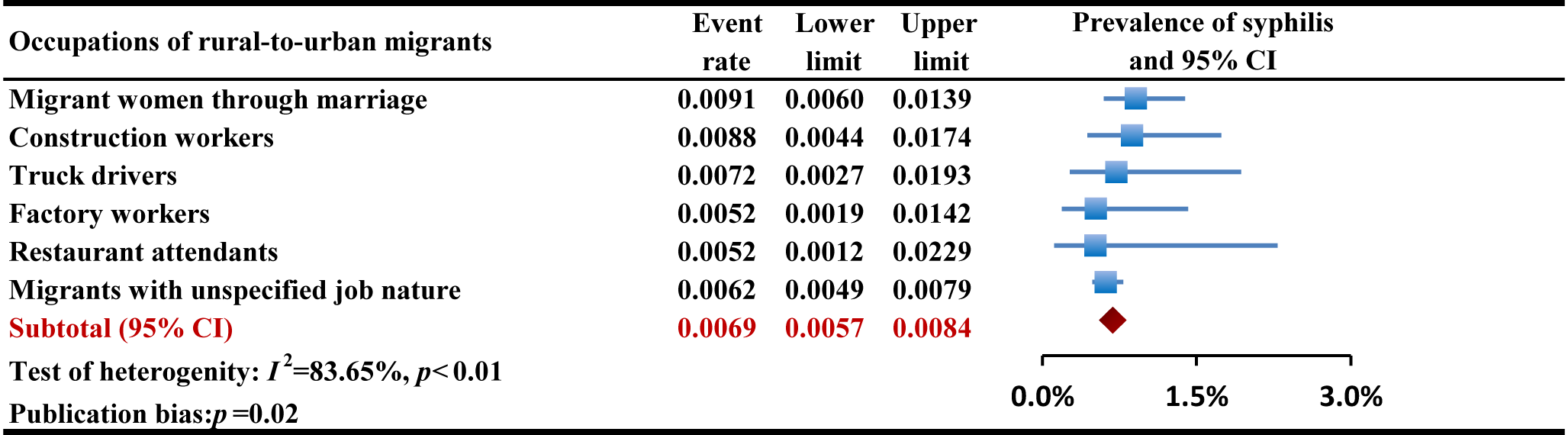
**

**Figure S3-2c Forest plots of meta-analysis of syphilis prevalence in migrant pregnant women in China**

**
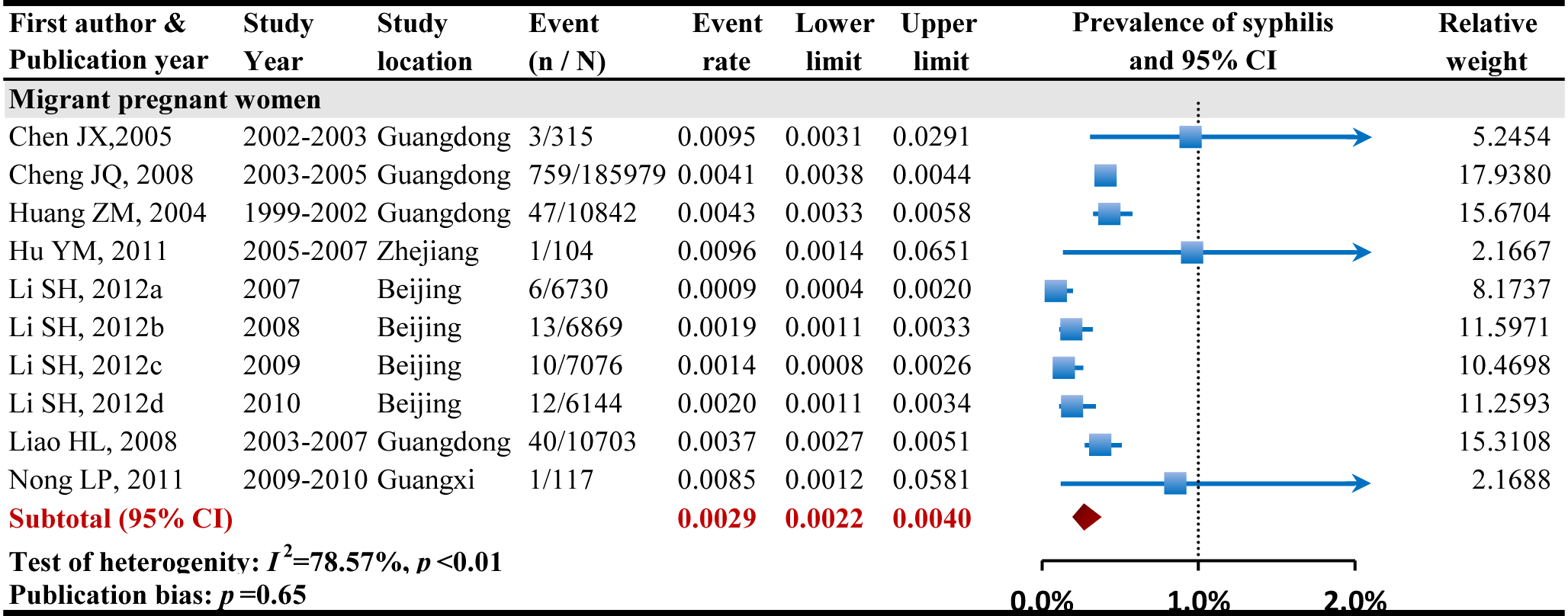
Figure S3-2d Forest plots of meta-analysis of syphilis prevalence in most at-risk populations with a migratory background in China**

**
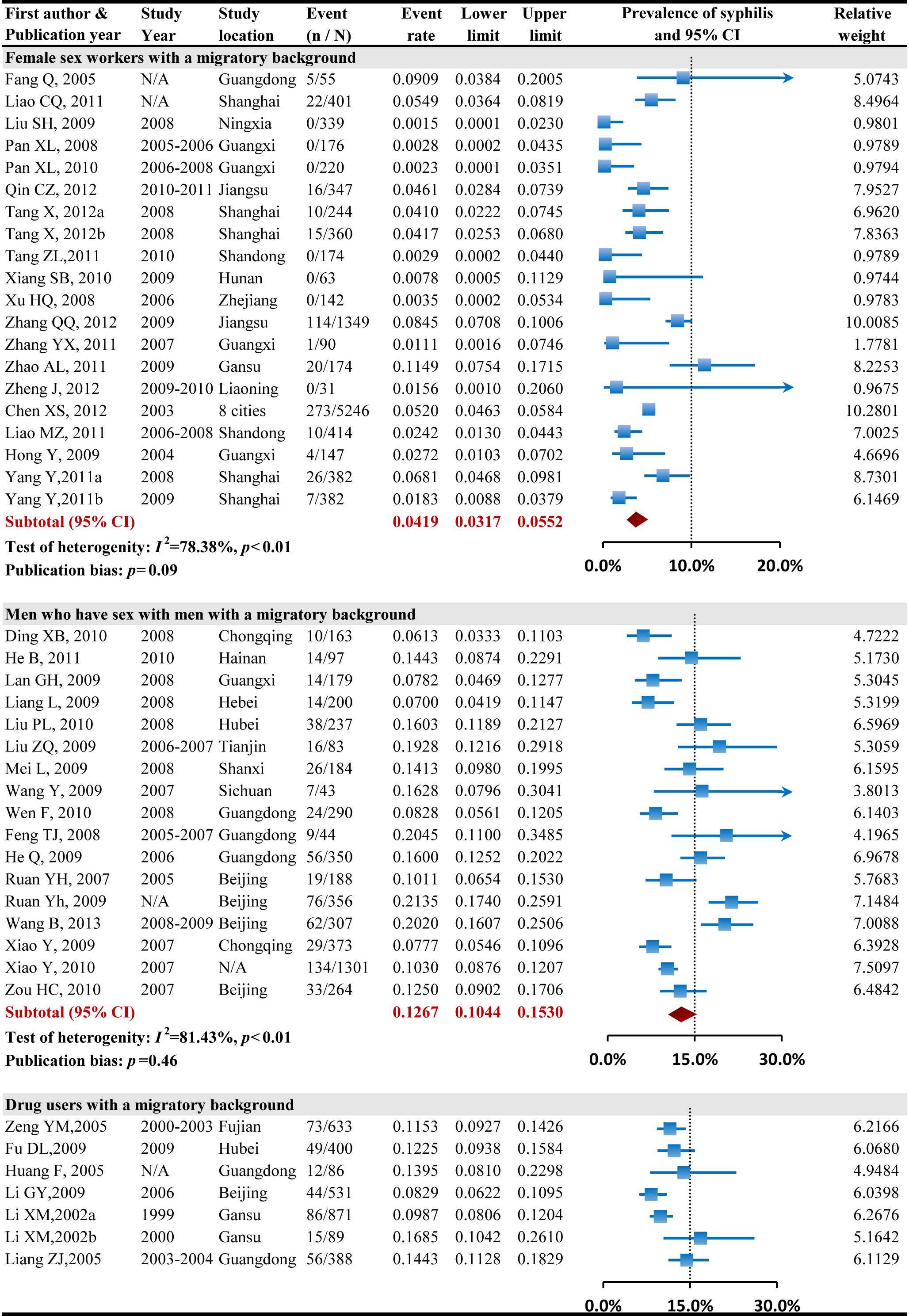
**

**Figure S3-2d Forest plots of meta-analysis of syphilis prevalence in most at-risk populations with a migratory background in China (Cont’d)
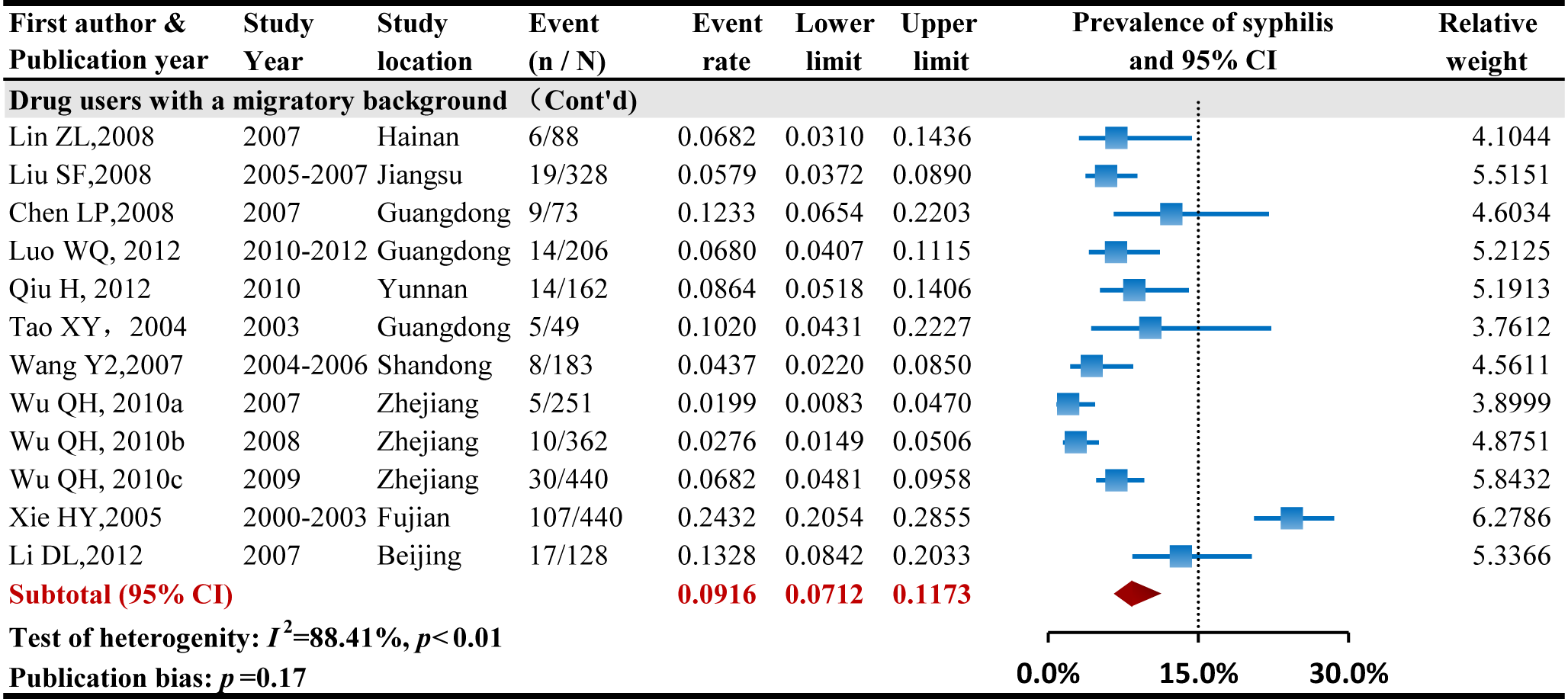
**

**Figure S3-3a Forest plots of meta-analysis of gonorrhea prevalence in individual occupational subgroups of rural-to-urban migrants in China**

**
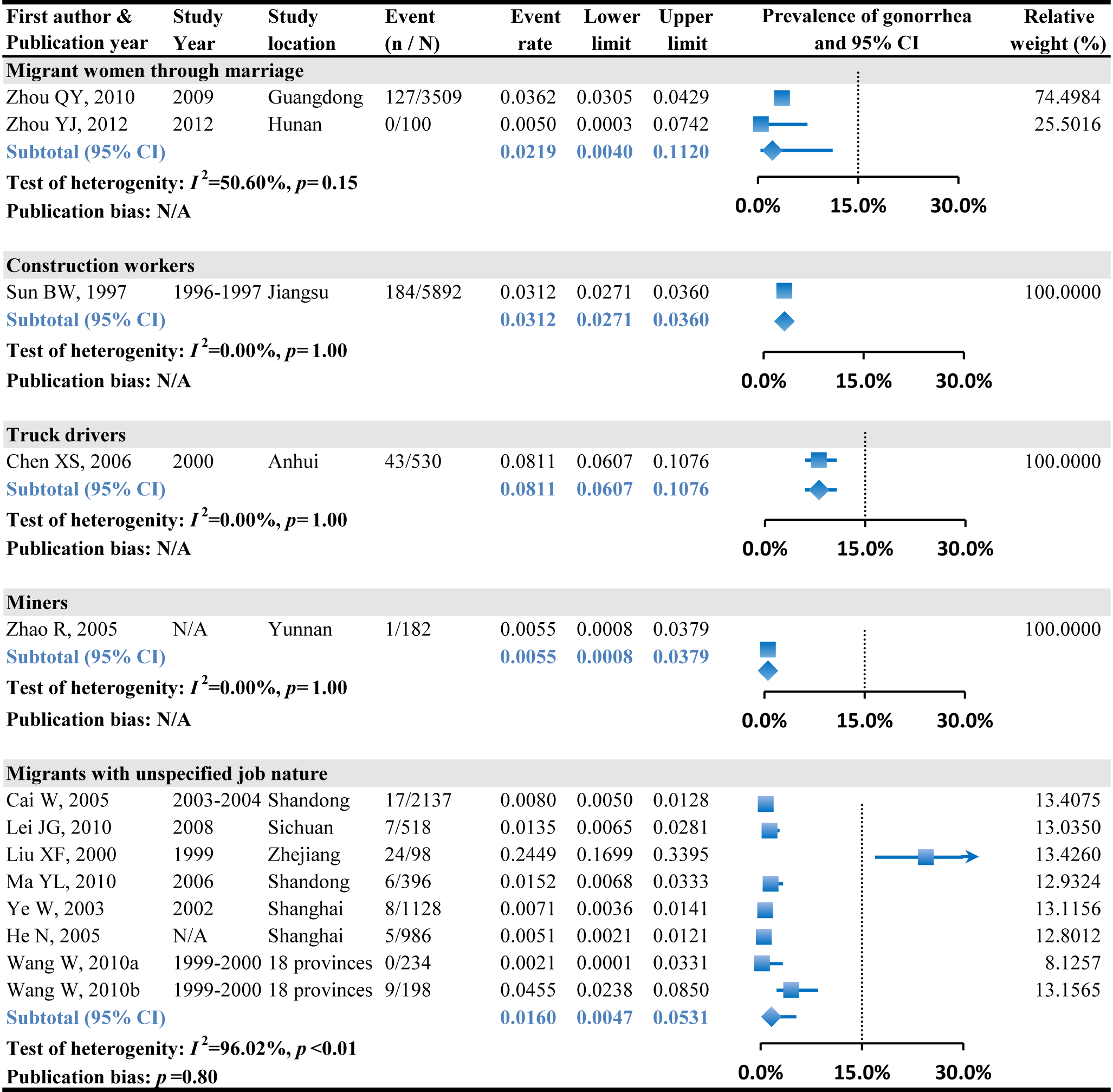
**

**Figure S3-3b Overall pooled estimate of gonorrhea prevalence in rural-to-urban migrants in China**

**
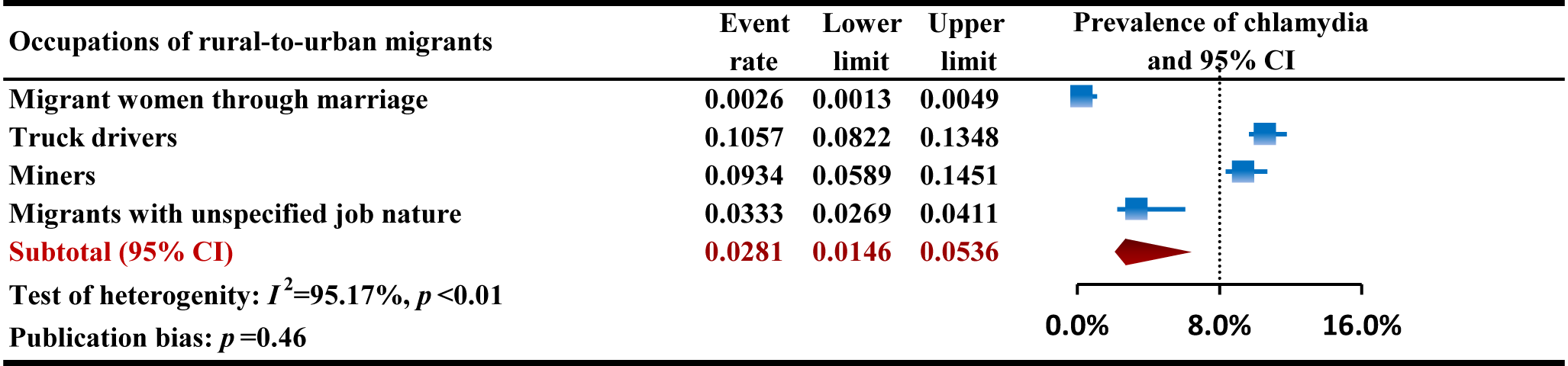
**

**Figure S3-3c Forest plots of meta-analysis of gonorrhea prevalence in most at-risk populations with a migratory background in China**

**
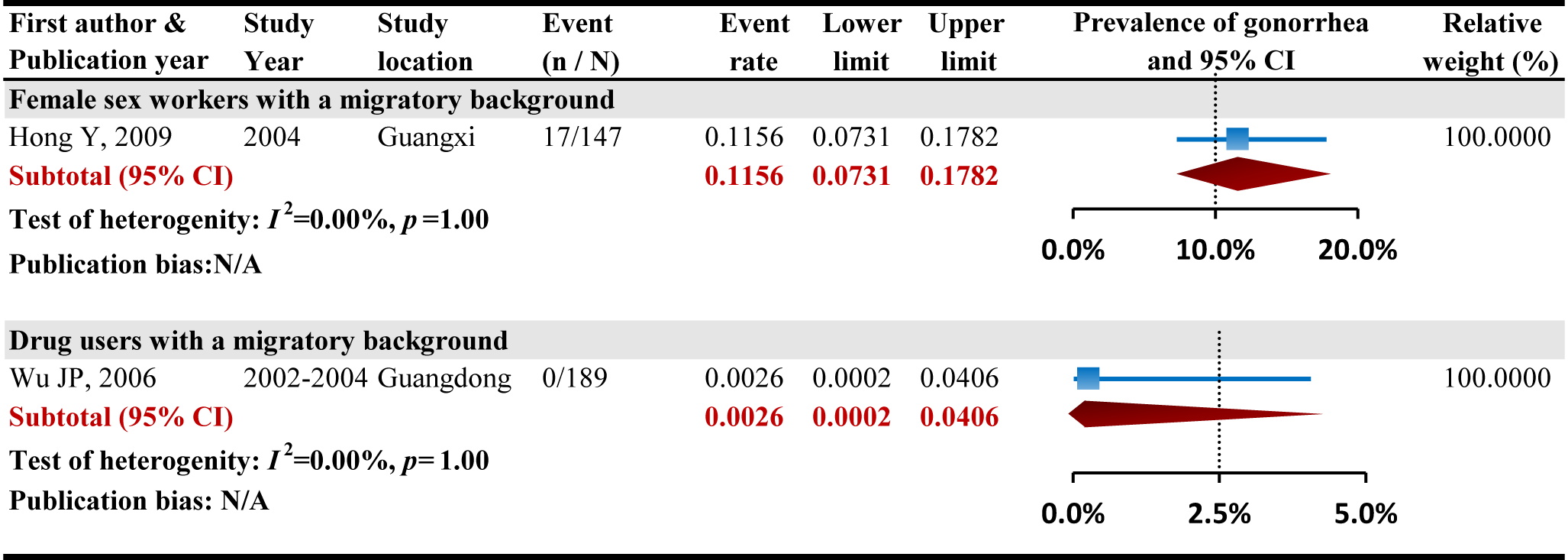
**

**Figure S3-4a Forest plots of meta-analysis of chlamydia prevalence in individual occupational subgroups of rural-to-urban migrants in China**

**
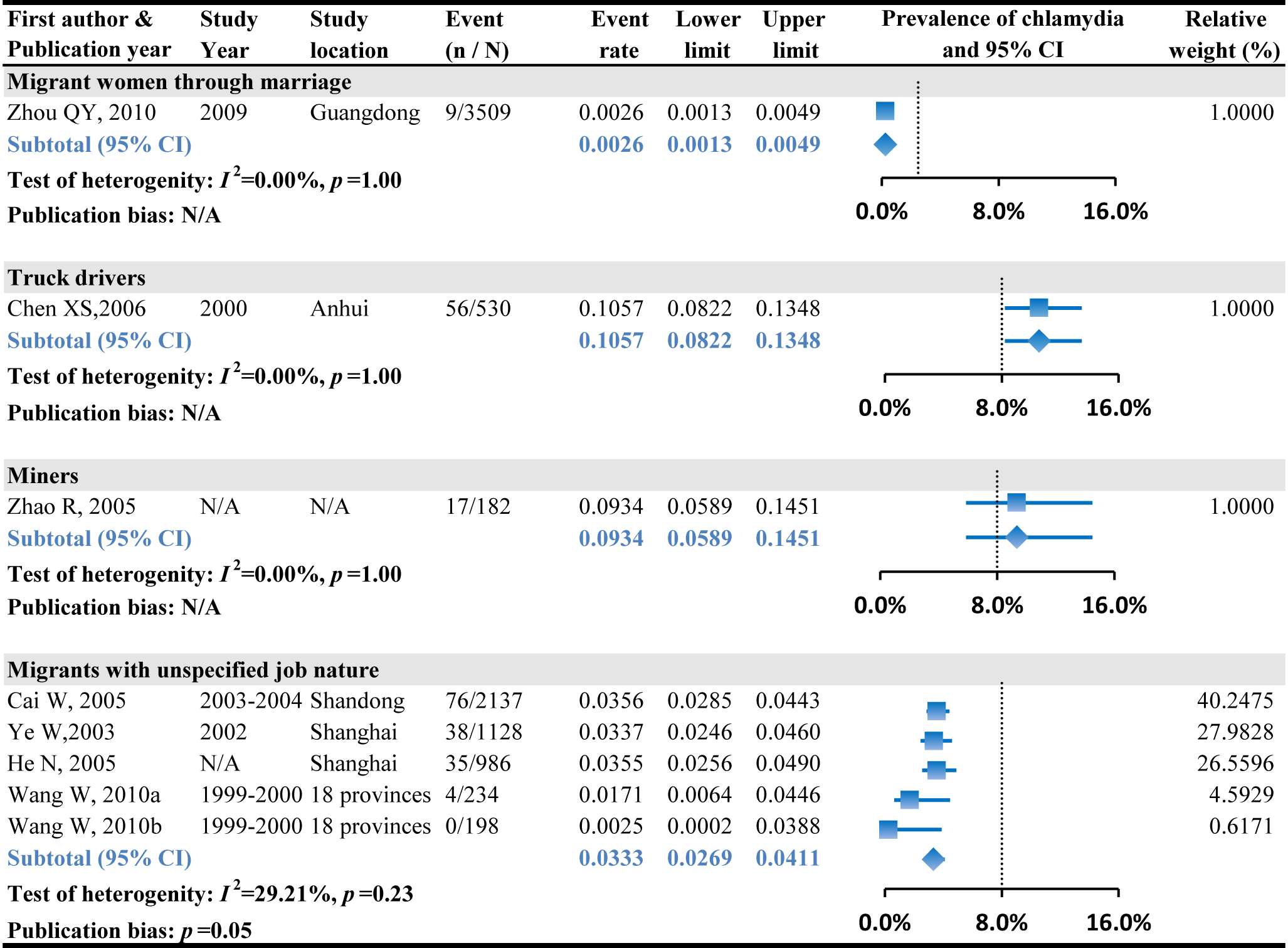
**

**Figure S3-4b Overall pooled estimate of chlamydia prevalence in rural-to-urban migrants in China**

**
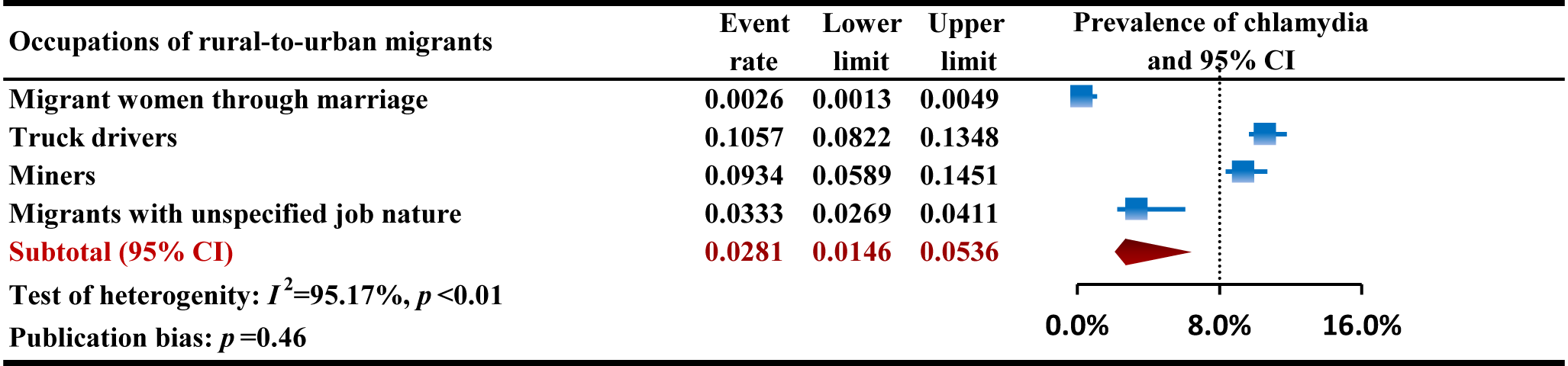
**

**Figure S3-4c Forest plots of meta-analysis of chlamydia prevalence in most at-risk populations with a migratory background in China**

**
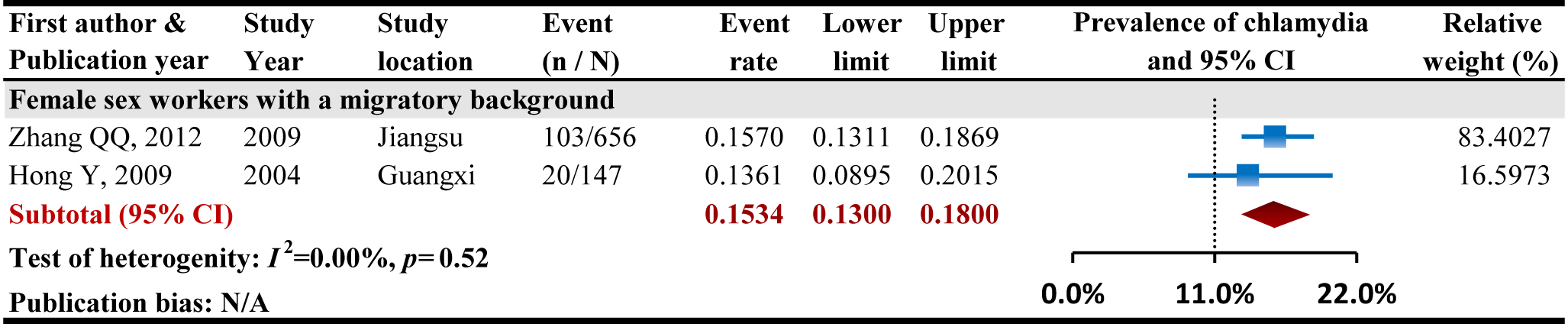
**

**Figure S3-5a Forest plots of meta-analysis of genital warts prevalence in individual occupational subgroups of rural-to-urban migrants in China**

**
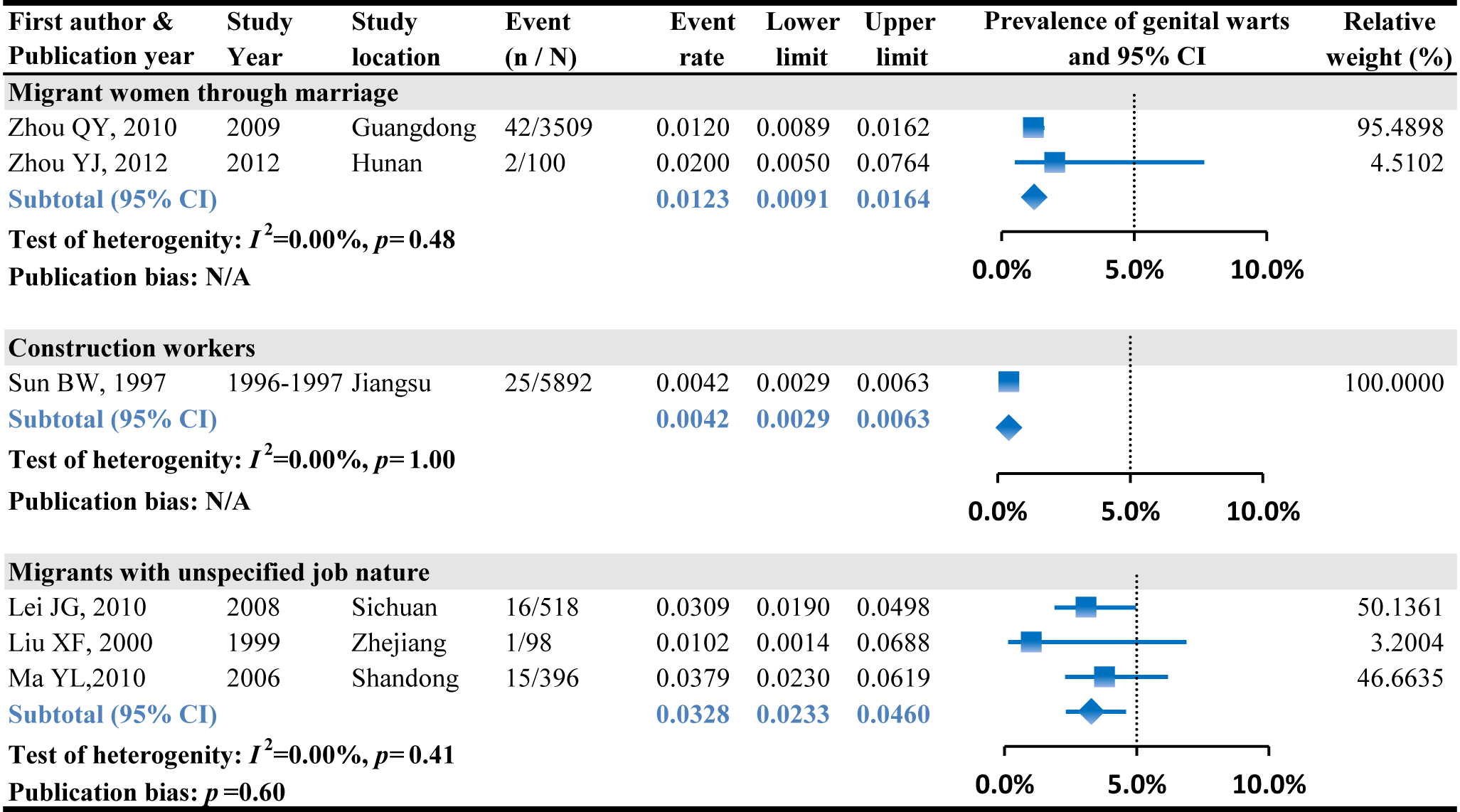
**

**Figure S3-5b Overall pooled estimate of genital warts prevalence in rural-to-urban migrants in China**

**
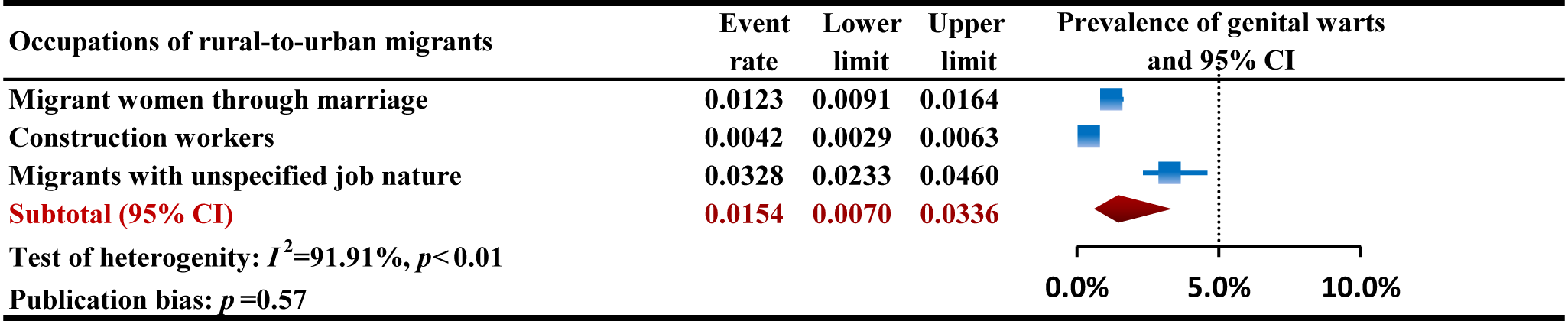
**

**Figure S3-6a Forest plots of meta-analysis of human papillomavirus (HPV) prevalence in individual occupational subgroups of rural-to-urban migrants in China**

**
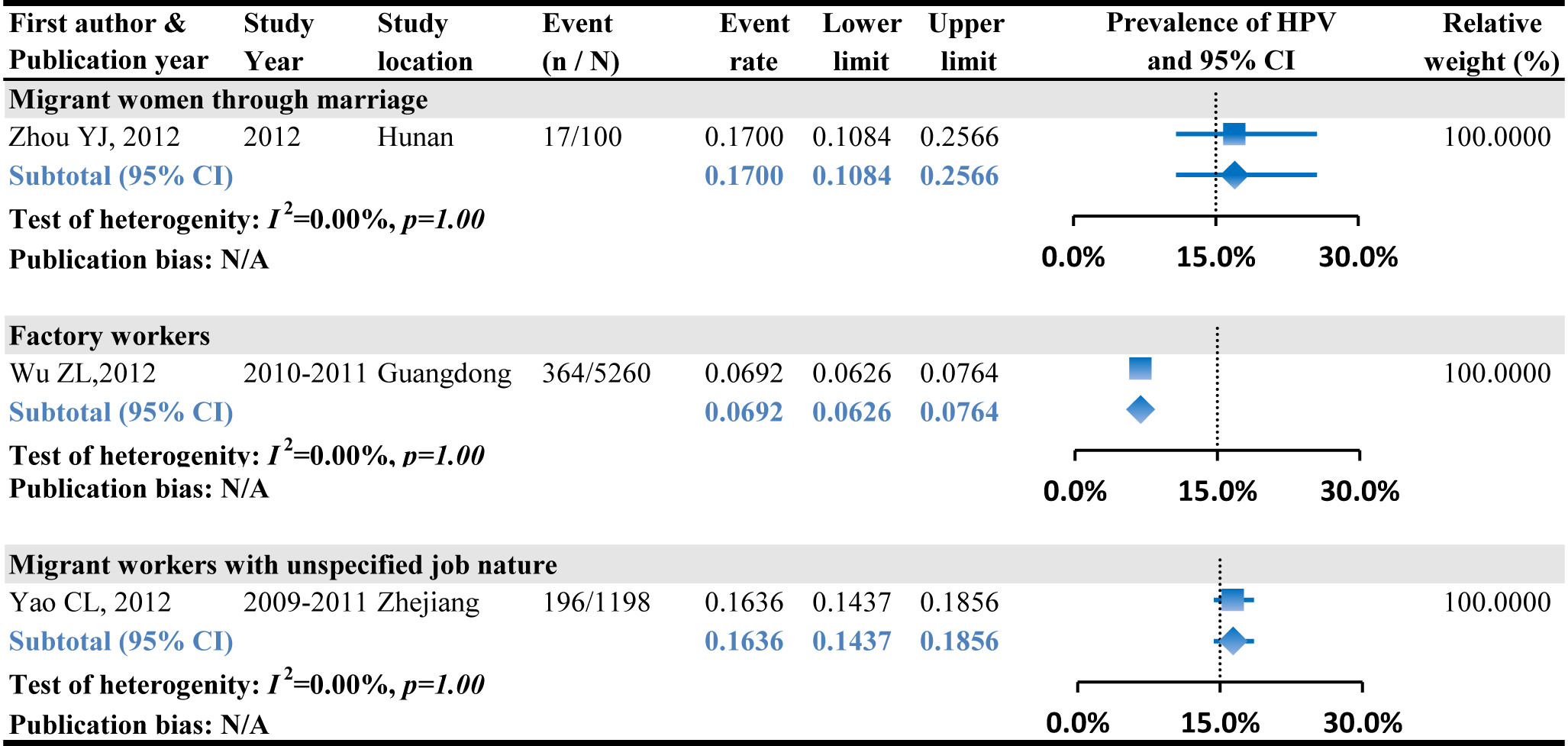
**

**Figure S3-6b Overall pooled estimate of HPV prevalence in rural-to-urban migrants in China
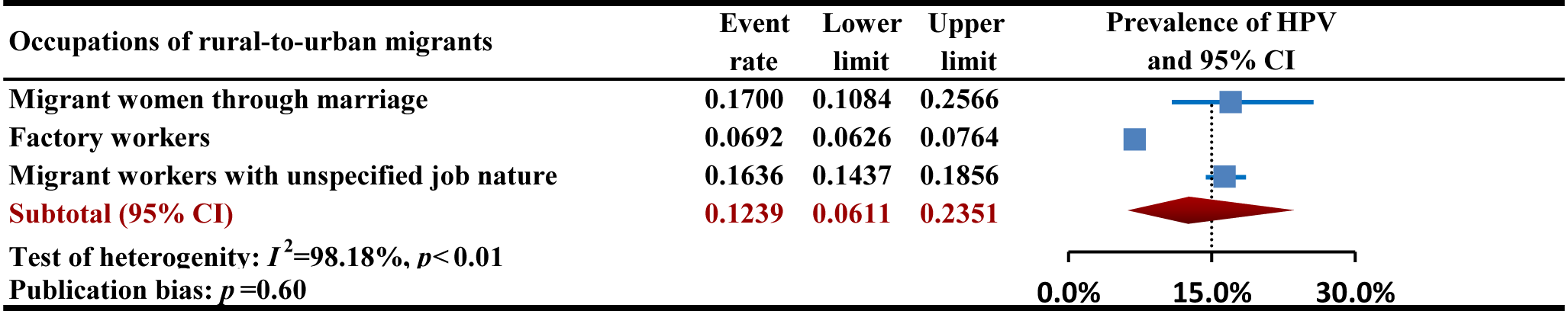
**

**Figure S3-6c Forest plots of meta-analysis of HPV prevalence in most at-risk populations with a migratory background in China**

**
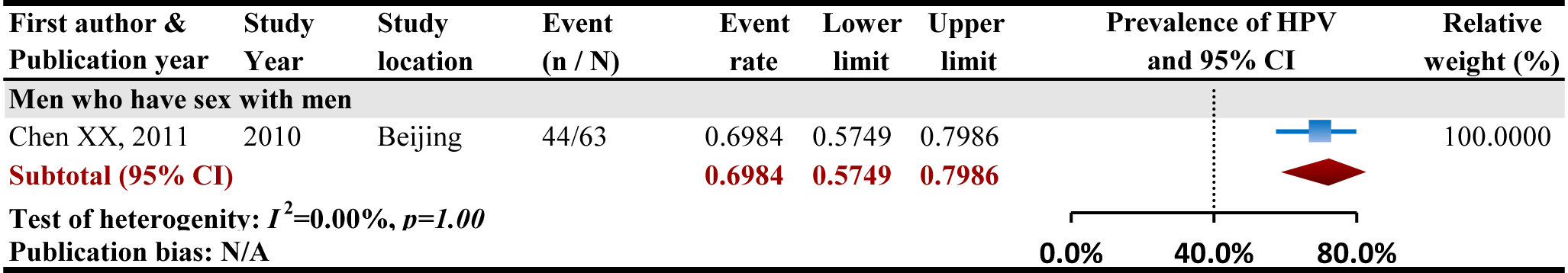
**

**Figure S3-7a Forest plots of meta-analysis of herpes simplex virus (HSV) prevalence in individual occupational subgroups of rural-to-urban migrants in China**

**
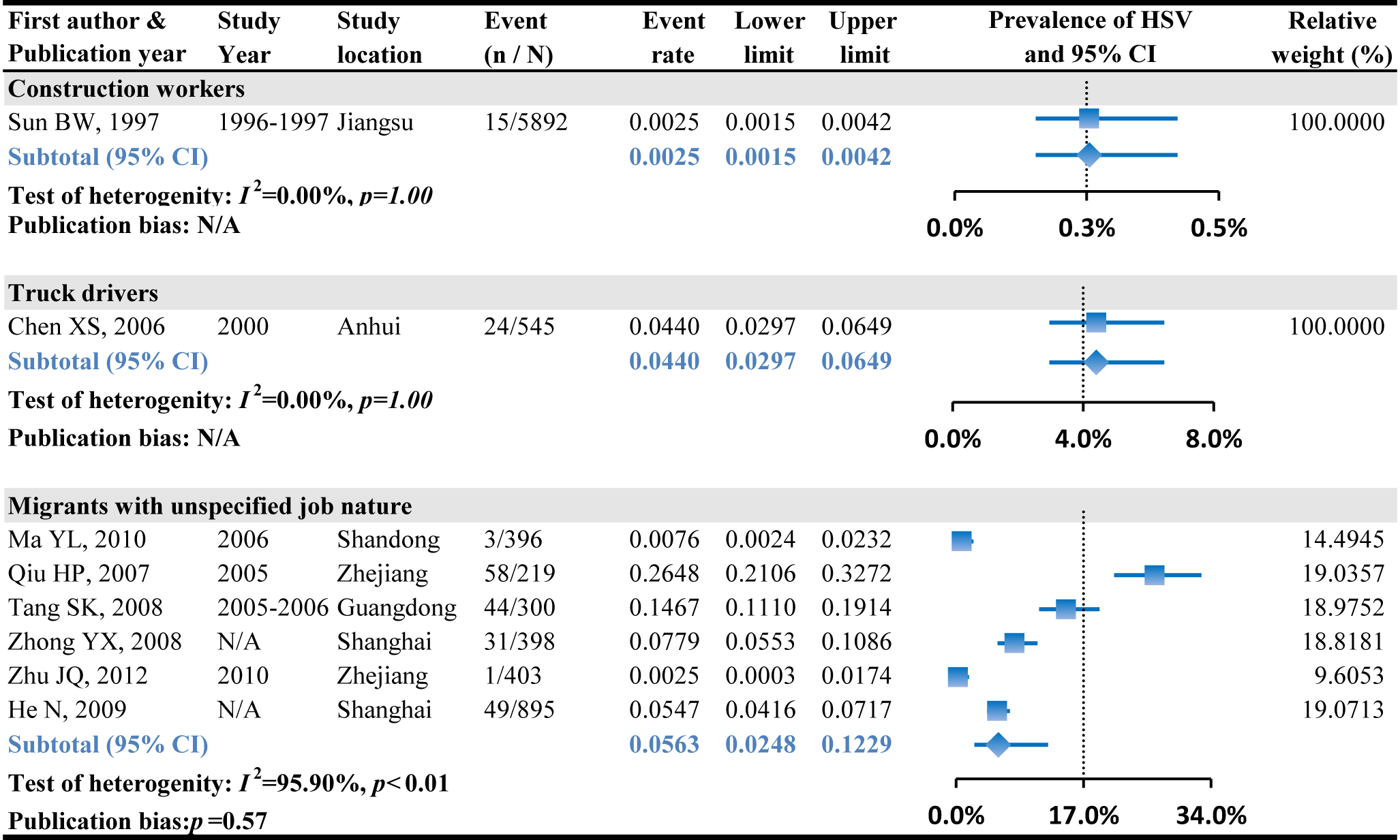
**

**Figure S3-7b Overall pooled estimate of HSV prevalence in rural-to-urban migrants in China
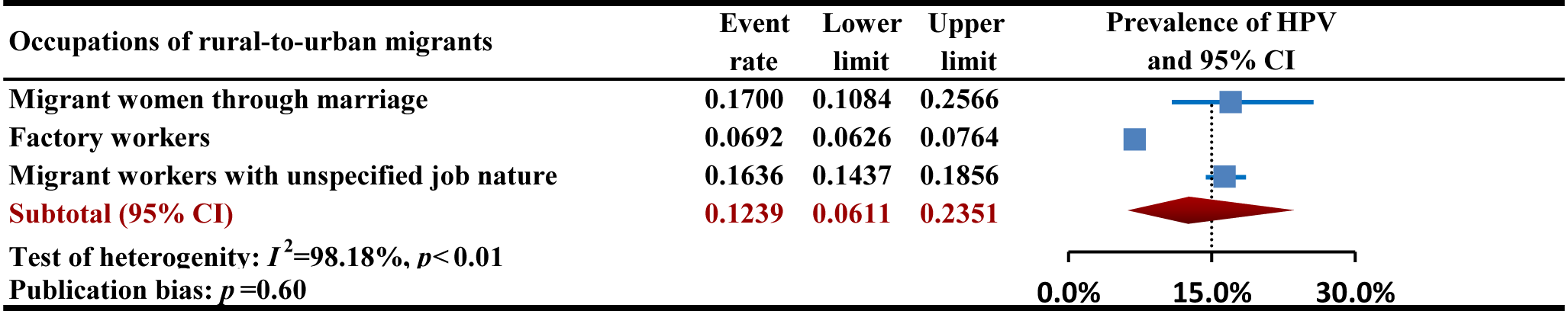
**

**Figure S3-7c Forest plots of meta-analysis of HSV prevalence in most at-risk populations with a migratory background in China**

**
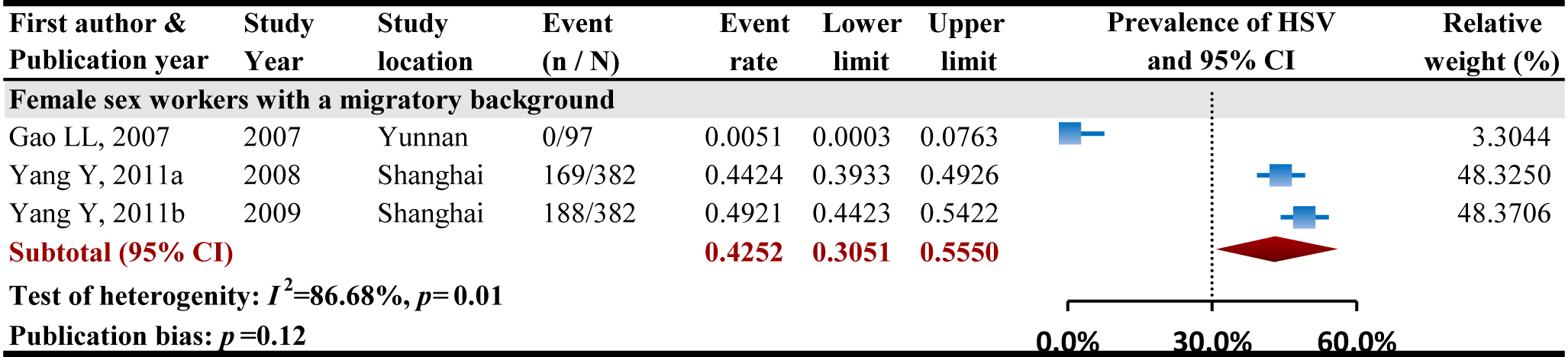
**

**Figure S3-8a Forest plots of meta-analysis of hepatitis B virus (HBV) prevalence in individual occupational subgroups of rural-to-urban migrants in China**

**
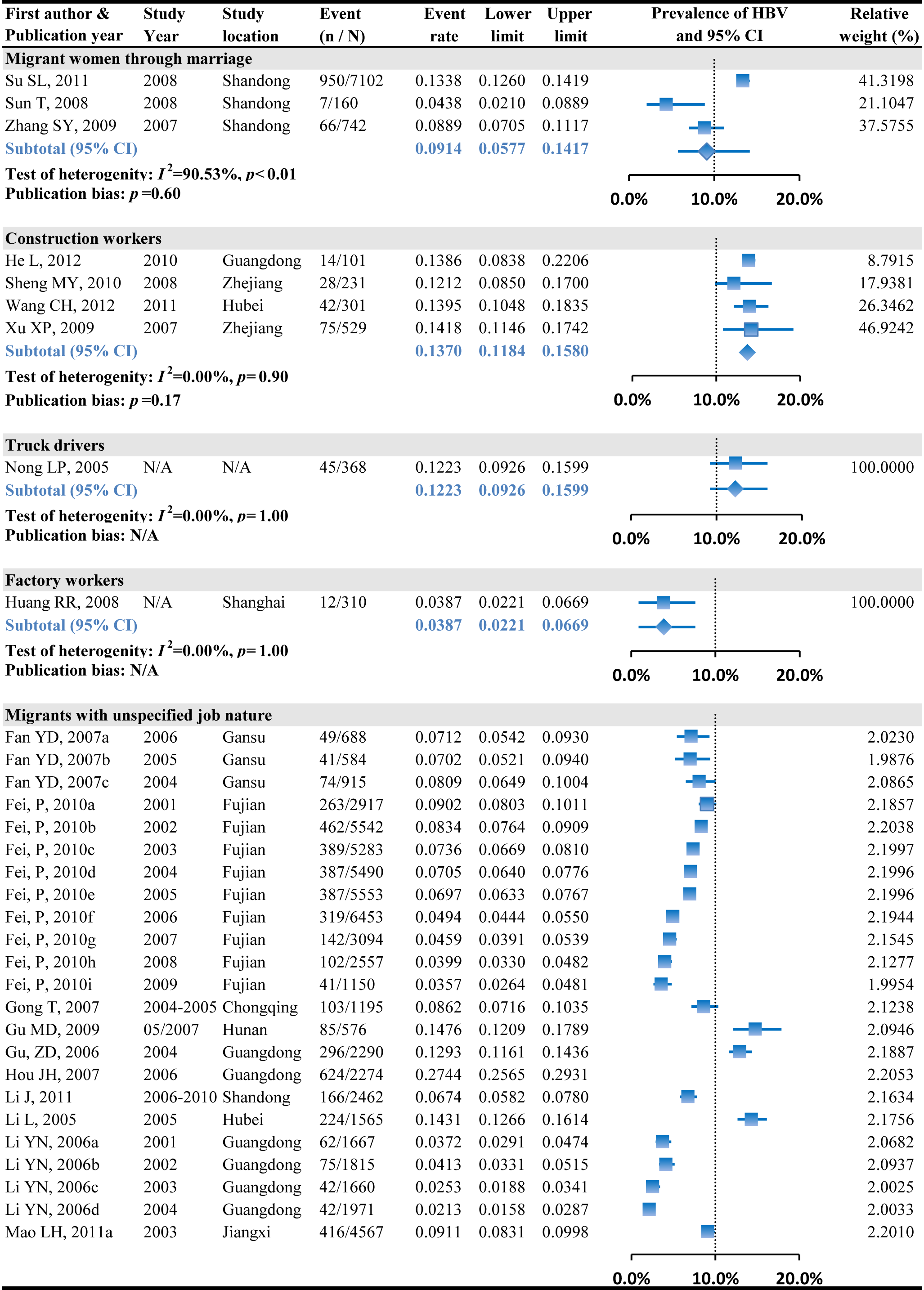
**

**Figure S3-8a F****orest plots of meta-analysis of HBV prevalence in individual occupational subgroups of rural-to-urban migrants in China (Cont’d)**

**
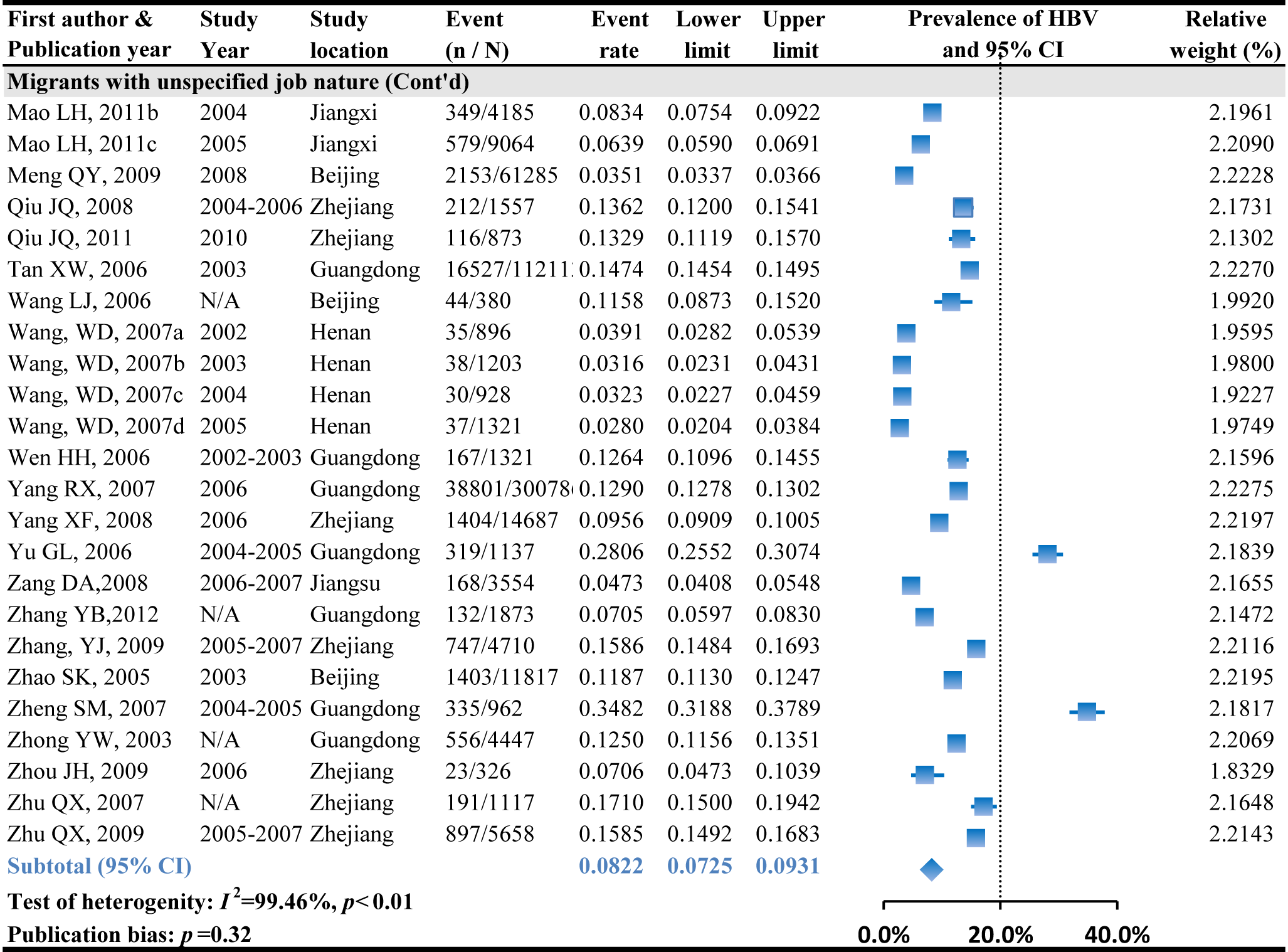
**

**Figure S3-8b Overall pooled estimate of HBV prevalence in rural-to-urban migrants in China
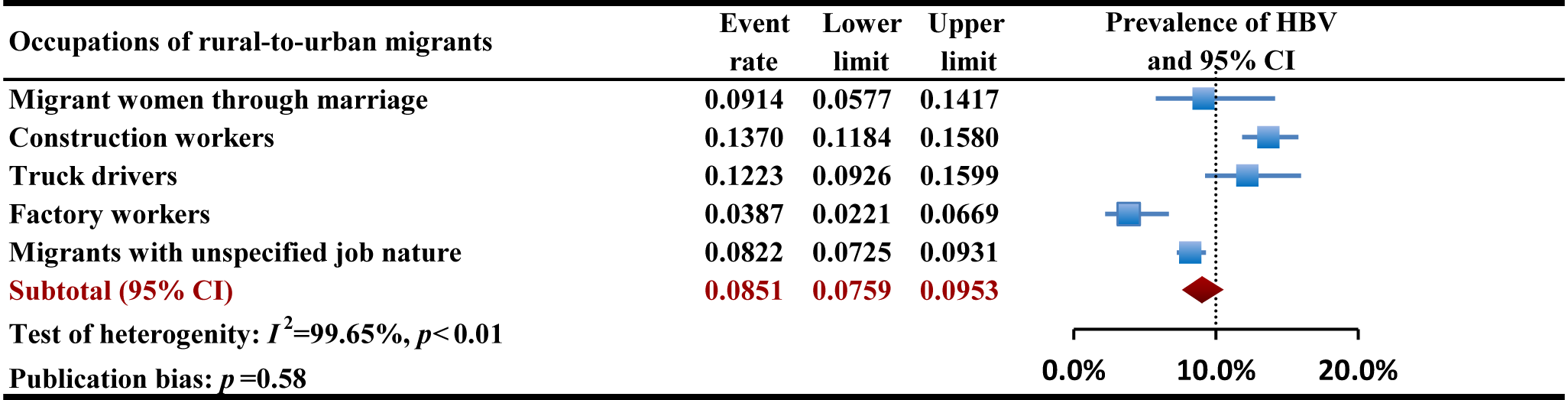
**

**Figure S3-8c Forest plots of meta-analysis of HBV prevalence in migrant pregnant women in China**

**
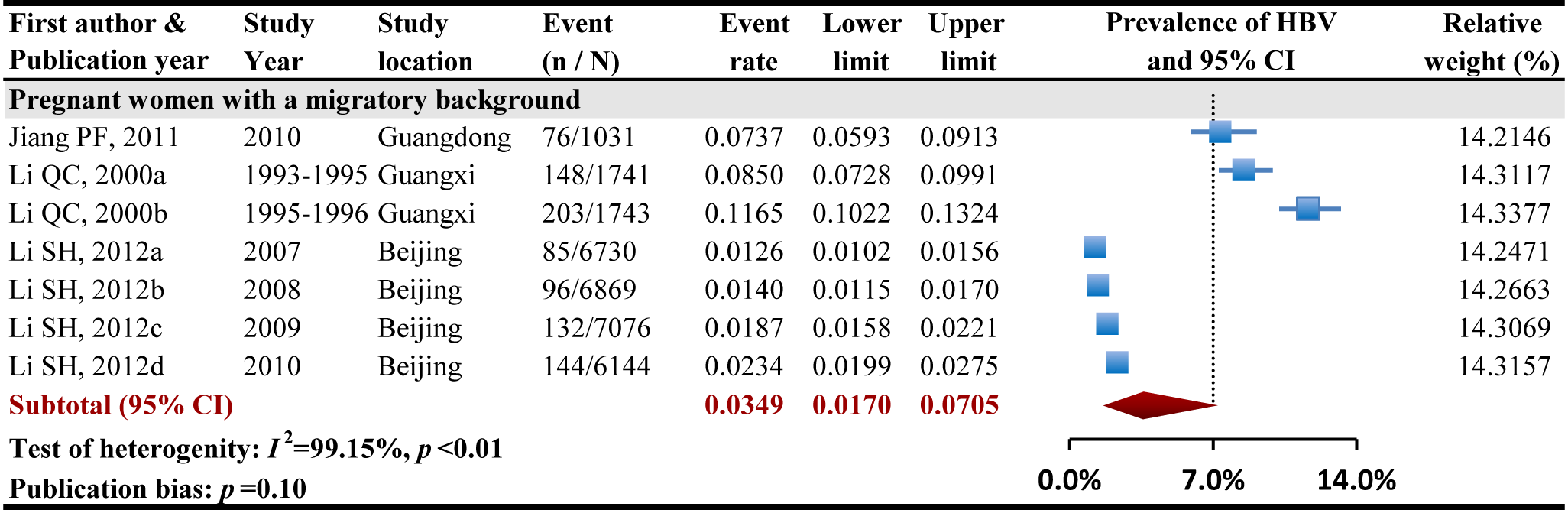
**

**Figure S3-8d Forest plots of meta-analysis of HBV prevalence in most at-risk populations with a migratory background in China**

**
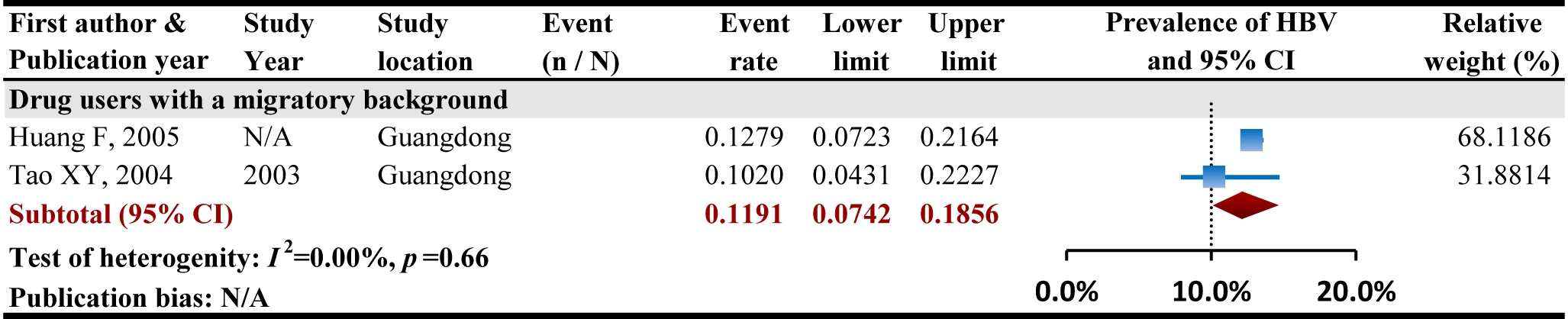
**

**Figure S3-9a Forest plots of meta-analysis of hepatitis C virus (HCV) prevalence in individual occupational subgroups of rural-to-urban migrants in China**

**
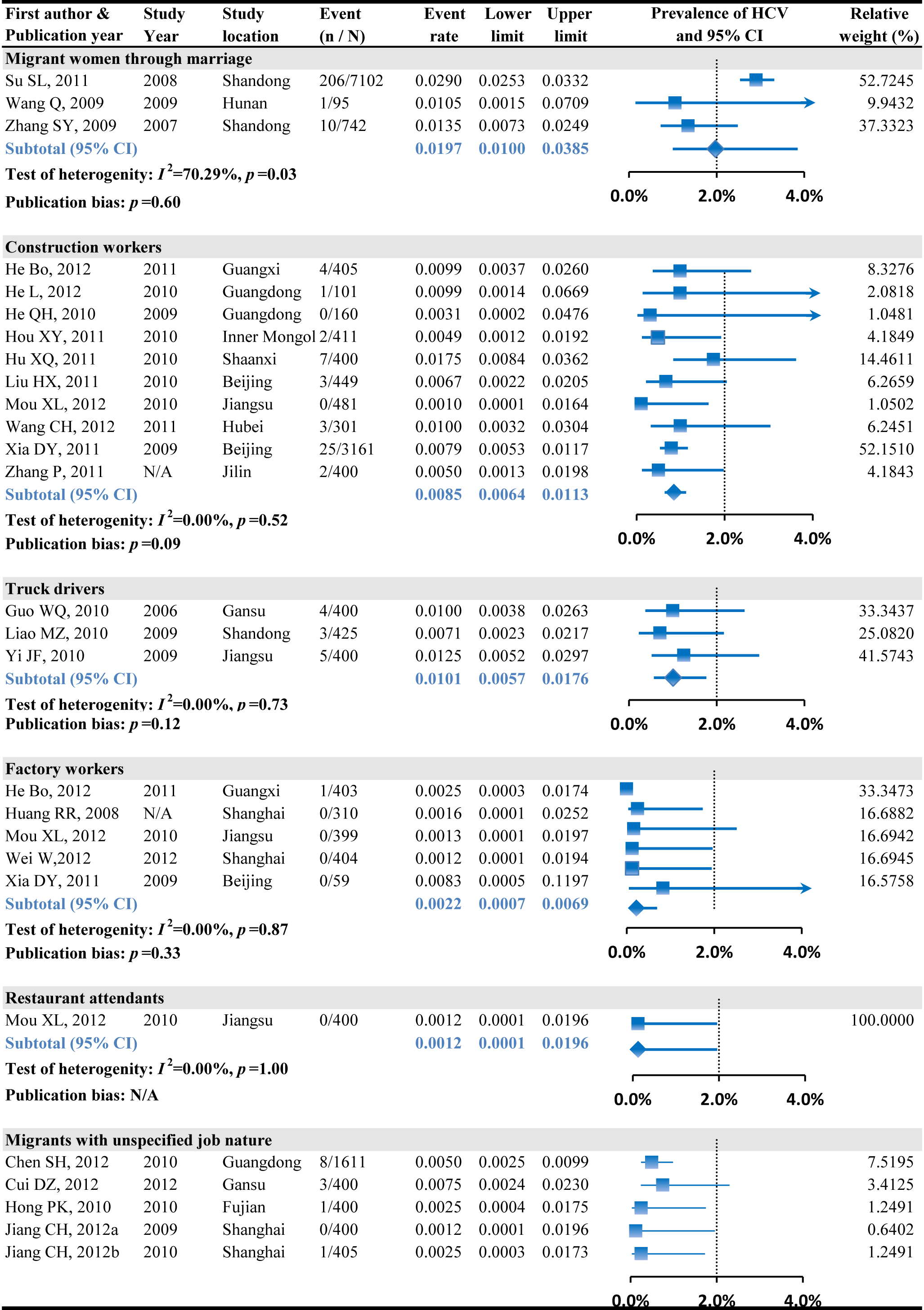
**

**Figure S3-9a Forest plots of meta-analysis of HCV prevalence in individual occupational subgroups of rural-to-urban migrants in China (Cont’d)
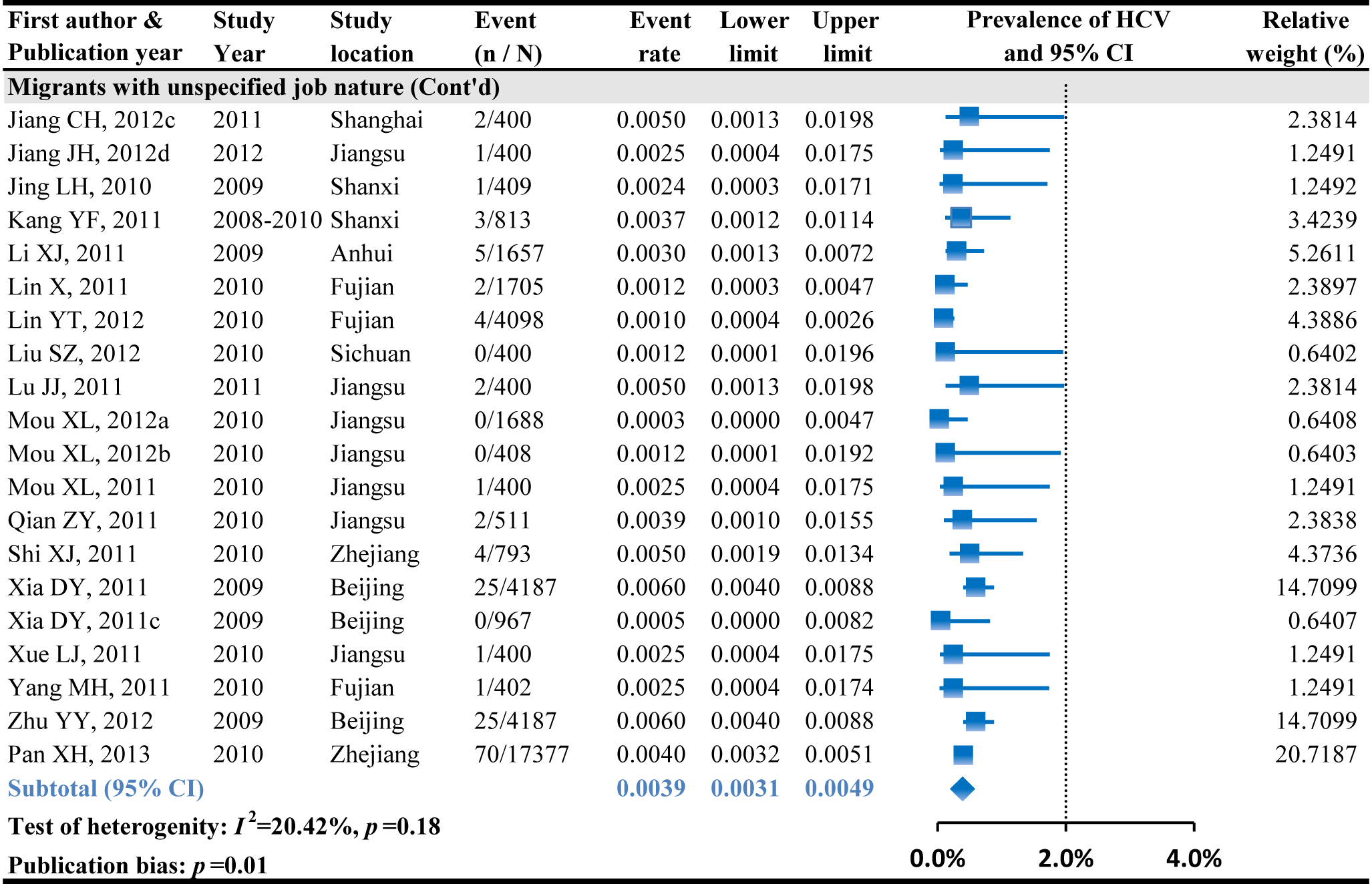
**

**Figure S3-9b Overall pooled estimate of HCV prevalence in rural-to-urban migrants in China
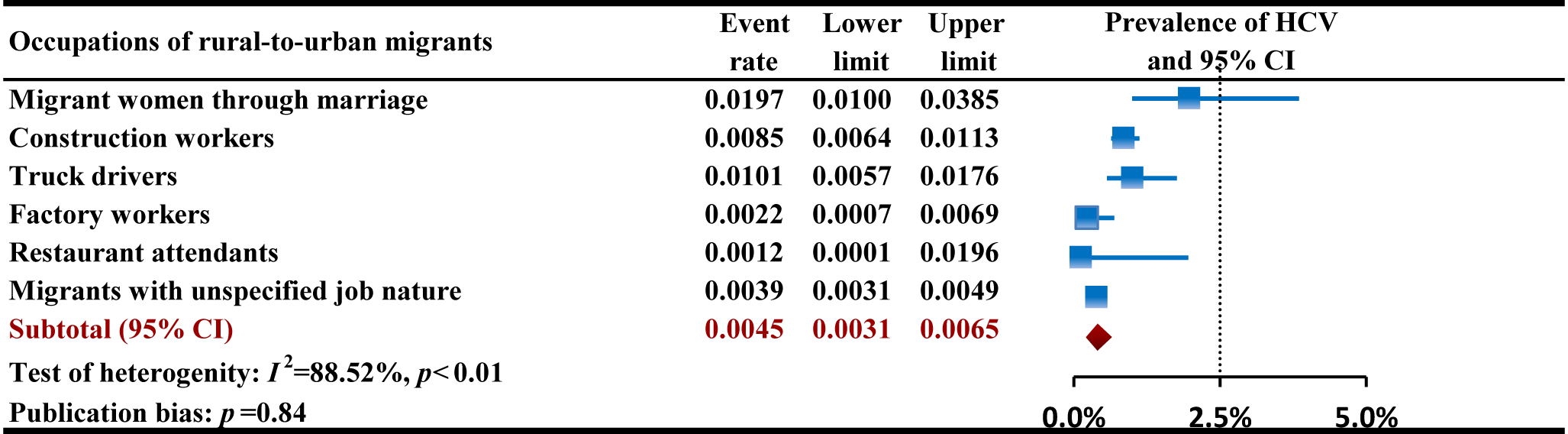
**

**Figure S3-9c Forest plots of meta-analysis of HCV prevalence in migrant pregnant women in China
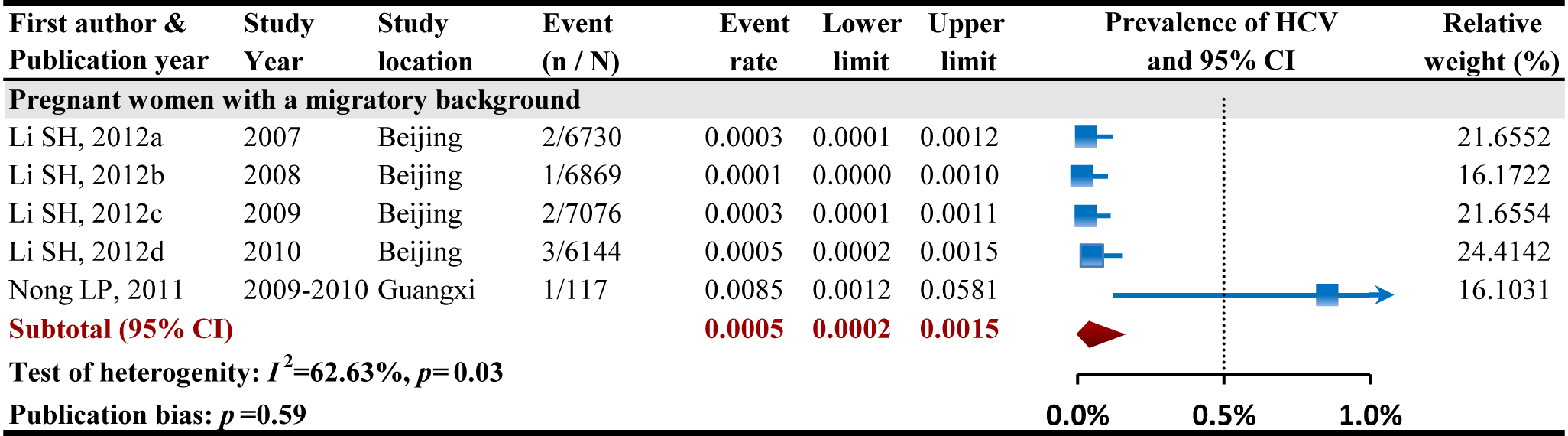
**

**Figure S3-9d Forest plots of meta-analysis of HCV prevalence in most at-risk populations with a migratory background in China**

**
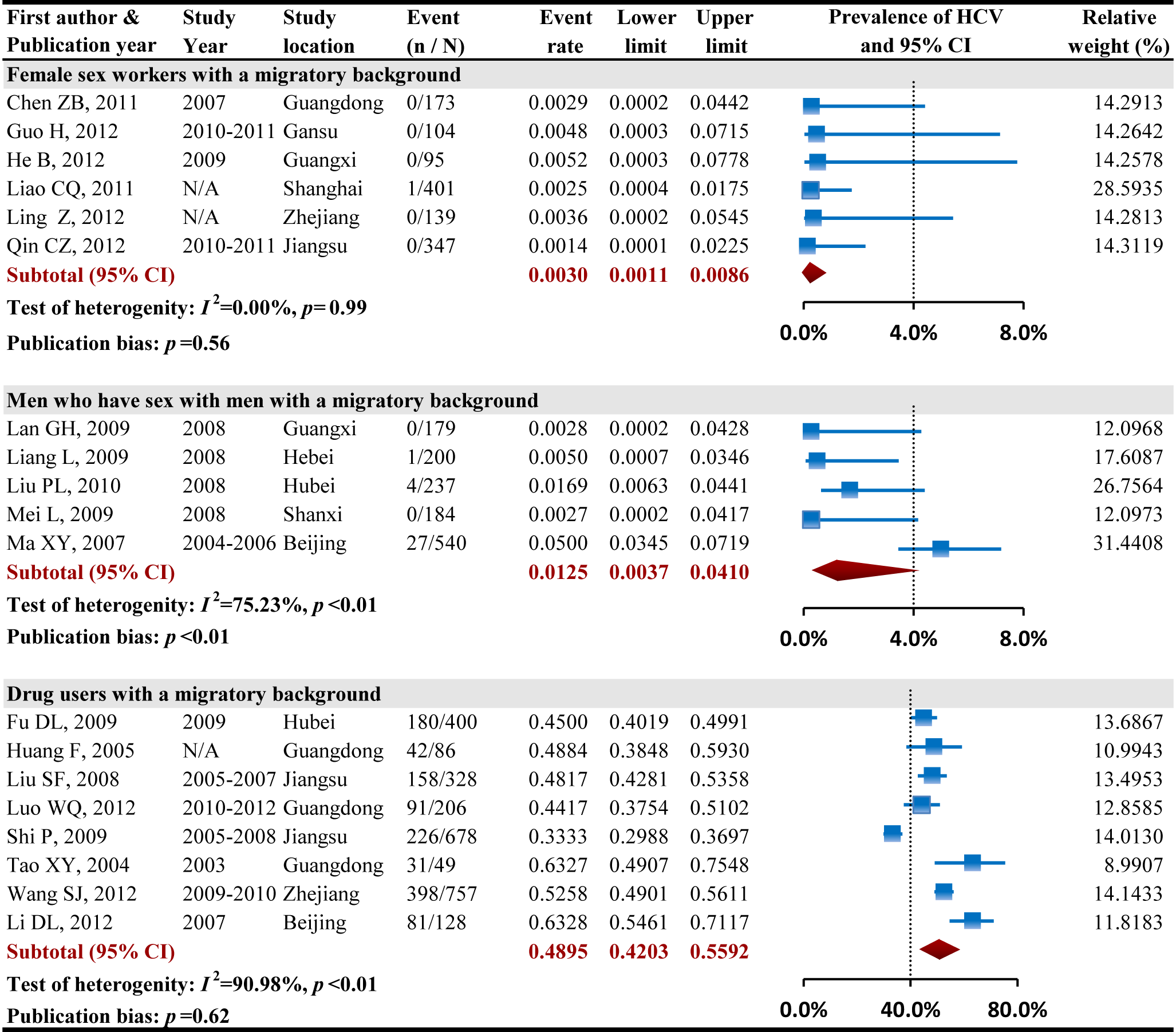
**
