# Supplementary figures and images for: Rural-to-urban migrants are at high risk of sexually transmitted and viral hepatitis infections in China: a systematic review and meta-analysis
Source: BMC Infect Dis. 2014 Sep 8;14:490. doi: 10.1186/1471-2334-14-490 (PMC4169821; doi:10.1186/1471-2334-14-490)

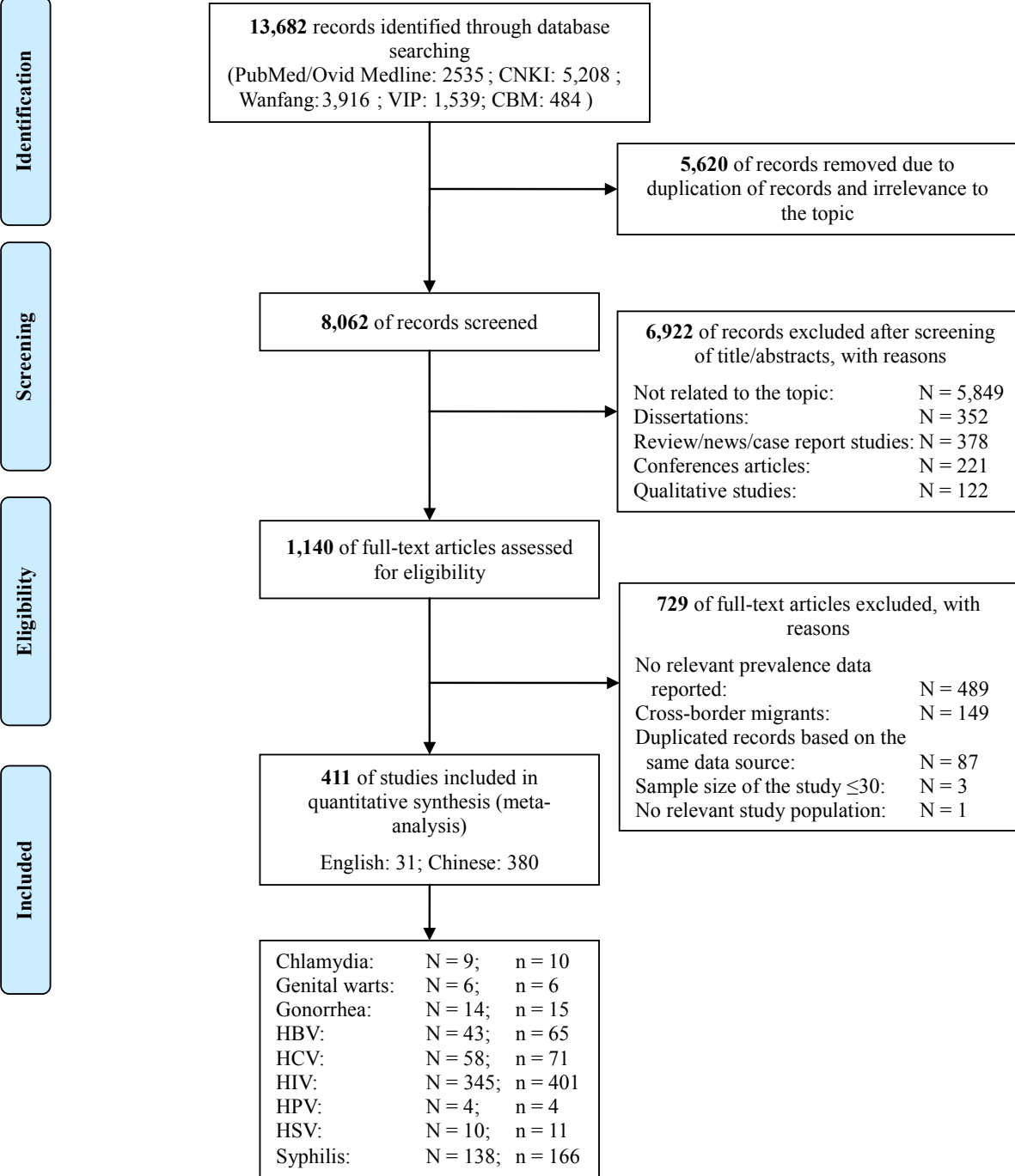

Supplement: Supplementary file 2 — Authors’ original file for figure 1 [file 12879_2014_3800_MOESM2_ESM.pdf]

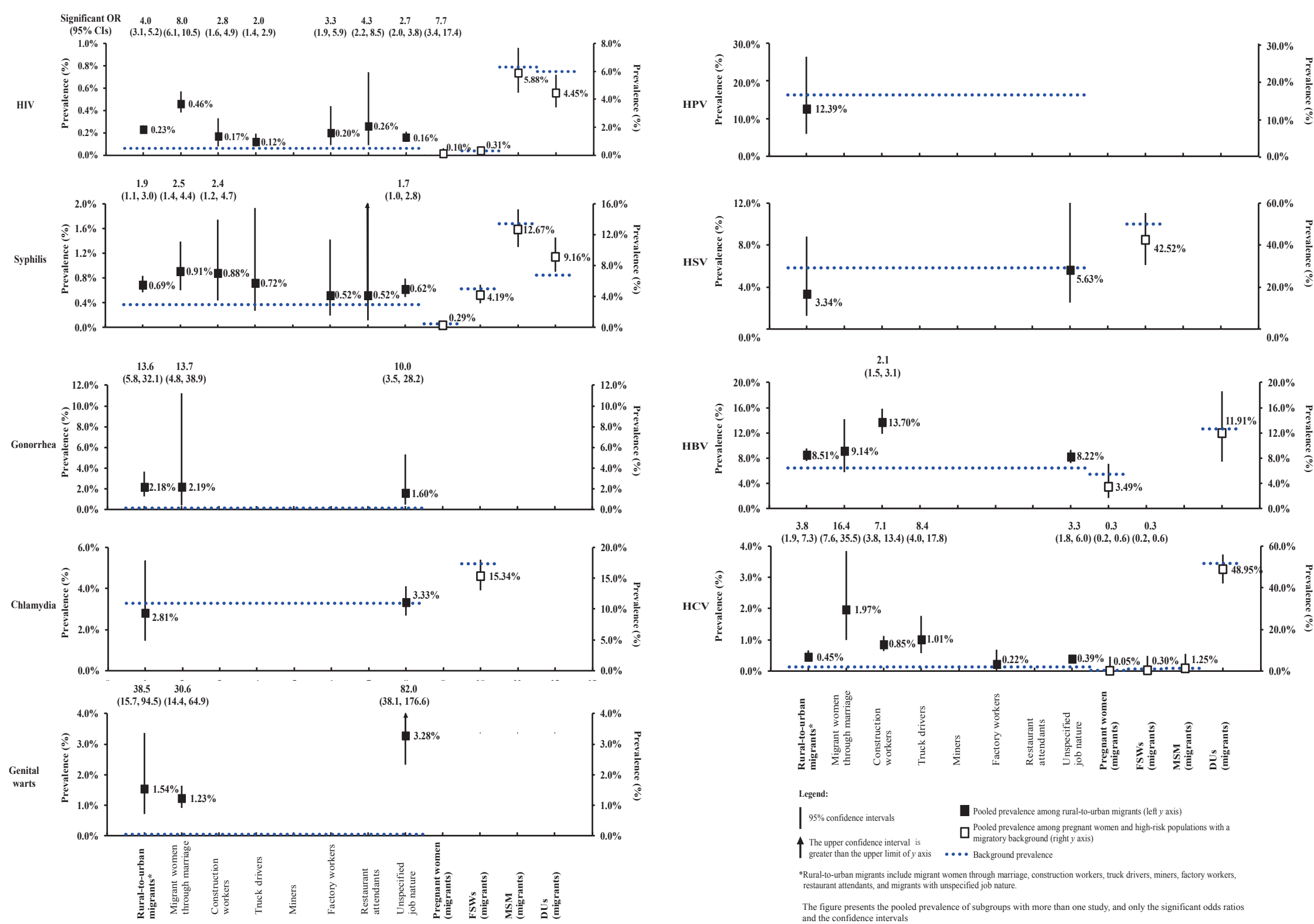

Supplement: Supplementary file 3 — Authors’ original file for figure 2 [file 12879_2014_3800_MOESM3_ESM.pdf]
